# Supplementary material for: Discovery of Novel Pimprinine and Streptochlorin Derivatives as Potential Antifungal Agents
Source: Mar Drugs. 2022 Nov 25;20(12):740. doi: 10.3390/md20120740 (PMC9787289; doi:10.3390/md20120740)

Discovery of novel pimprinine and streptochlorin derivatives as potential antifungal agents

Jing-Rui Liu[1], Jia-Mu Liu[1], Ya Gao[1], Zhan Shi[1], Ke-Rui Nie[1], Dale Guo[2], Fang Deng[2], Haifeng Zhang[3], Abdallah S. Ali[4], Ming-Zhi Zhang[1]*, Wei-Hua Zhang[1] and Yu-Cheng Gu[5]

[1] *Jiangsu Key Laboratory of Pesticide Science, College of Sciences, Nanjing Agricultural University, Nanjing 210095, China*

[2] *State Key Laboratory Breeding Base of Systematic Research Development and Utilization of Chinese Medicine Resources, School of Pharmacy, Chengdu University of Traditional Chinese Medicine, Chengdu, 611137, China*

[3] *Department of Plant Pathology, College of Plant Protection, Nanjing Agricultural University, and Key Laboratory of Integrated Management of Crop Diseases and Pests, Ministry of Education, Nanjing, China*

[4] *Department of Microbiology, Faculty of Agriculture, Cairo University, 12613, Giza, Egypt*

[5] *Syngenta Jealott's Hill International Research Centre, Bracknell, Berkshire RG42 6EY, United Kingdom*

Corresponding Authors:

Prof. Ming-Zhi Zhang, E-mail: mzzhang@njau.edu.cn, Nanjing Agricultural University, Nanjing 210095, P.R. China

1. Compound Data

Intermediate:

*1-(1H-indol-3-yl)ethan-1-one (**2**)*: Yellow crystal; Yield: 71%. m.p.: 188.6-191.8℃. ^{1}H NMR (400 MHz, Acetone-*d6*) δ 10.99 (s, 1H), 8.40-8.31 (m, 1H), 8.23 (d, *J* = 3.2 Hz, 1H), 7.54-7.49 (m, 1H), 7.29-7.17 (m, 2H), 2.49 (s, 3H).

target compounds:

*1.1 5-(1H-indol-3-yl)oxazole (**3a**)*: Yellow powder; Yield: 51%. m.p.:174.7-176.5 °C. ^{1}H NMR (400 MHz, DMSO-d_6) δ 11.62 (s, 1H), 8.36 (d, *J* = 2.0 Hz, 1H), 7.90 (s, 1H), 7.83 (d, *J* = 2.8 Hz, 1H), 7.54 – 7.47 (m, 2H), 7.23 (t, *J* = 7.6 Hz, 1H), 7.20 – 7.14 (m, 1H). ^{13}C NMR (100 MHz, DMSO-d_6) δ 149.7, 147.8, 136.5, 123.7, 123.6, 122.3, 120.3, 119.5, 118.8, 112.2, 103.7. HRMS (MALDI): m/z calcd for $C_{11}H_8N_2O$ ([M + H]$^+$) 185.0709, Found 185.0709.

*1.2 5-(1H-indol-3-yl)-2-methyloxazole (**3b**)*: Light yellow powder; Yield: 53%. m.p.: 202.7-204.8 °C. ^{1}H NMR (400 MHz, DMSO-d_6) δ 11.50 (s, 1H), 7.82 (d, *J* = 8.0 Hz, 1H), 7.71 (d, *J* = 2.8 Hz, 1H), 7.46 (d, *J* = 8.0 Hz, 1H), 7.27 (s, 1H), 7.22 – 7.10 (m, 2H), 2.47 (s, 3H). ^{13}C NMR (100 MHz, Acetone-d_6) δ 158.5, 147.8, 136.8, 124.2, 122.4, 122.3, 120.2, 119.6, 119.6, 111.9, 105.0, 12.9. HRMS (MALDI): m/z calcd for $C_{12}H_{10}N_2O$ ([M + H]$^+$) 199.0866, Found 199.0862.

*1.3 2-ethyl-5-(1H-indol-3-yl)oxazole (**3c**)*: Light yellow powder; Yield: 42%. m.p.: 157.4-158.6 °C. ^{1}H NMR (400 MHz, DMSO-d_6) δ 11.53 (s, 1H), 7.83 (d, *J* = 8.0 Hz, 1H), 7.73 (d, *J* = 2.8 Hz, 1H), 7.46 (d, *J* = 8.0 Hz, 1H), 7.29 (s, 1H), 7.16 (dt, *J* = 25.2, 7.2 Hz, 2H), 2.82 (q, *J* = 7.6 Hz, 2H), 1.30 (t, *J* = 7.6 Hz, 3H). ^{13}C NMR (100 MHz, Acetone-d_6) δ 162.9, 147.7, 136.8, 124.2, 122.5, 122.3, 120.2, 119.7, 119.4, 111.9, 105.1, 21.2, 10.8. HRMS (MALDI): m/z calcd for $C_{13}H_{12}N_2O$ ([M + H]$^+$) 213.1022, Found 213.1087.

*1.4 5-(1H-indol-3-yl)-2-isobutyloxazole (**3d**)*: Yellow powder; Yield: 43%. m.p.: 137.9-139.6 °C. ^{1}H NMR (400 MHz, DMSO-d_6) δ 11.52 (s, 1H), 7.81 (d, *J* = 8.0 Hz, 1H), 7.71 (d, *J* = 2.8 Hz, 1H), 7.45 (d, *J* = 8.0 Hz, 1H), 7.28 (s, 1H), 7.22 – 7.09 (m, 2H), 2.67 (d, *J* = 7.2 Hz, 2H), 2.24 – 2.04 (m, 1H), 0.97 (d, *J* = 6.8 Hz, 6H). ^{13}C NMR (100 MHz, Acetone-d_6) δ 161.4, 147.7, 136.8, 124.2, 122.5, 122.3, 120.2, 119.7, 119.4, 111.9, 105.1, 36.6, 27.4, 21.8. HRMS (MALDI): m/z calcd for $C_{15}H_{16}N_2O$ ([M + H]$^+$) 241.1335, Found 241.1331.

*1.5 2-cyclohexyl-5-(1H-indol-3-yl)oxazole (**3e**)*: Light pink powder; Yield: 53%. m.p.: 177.6-179.3 °C. ^{1}H NMR (400 MHz, Acetone-d_6) δ 10.67 (s, 1H), 7.90 (d, *J* = 8.0 Hz, 1H), 7.74 (d, *J* = 2.4 Hz, 1H), 7.51 (d, *J* = 8.0 Hz, 1H), 7.27 – 7.22 (m, 1H), 7.22 – 7.14 (m, 2H), 2.87 (dq, *J* = 11.2, 4.0 Hz, 1H), 2.17 – 2.09 (m, 2H), 1.91 – 1.79 (m, 2H), 1.78 – 1.67 (m, 2H), 1.66 – 1.47 (m, 2H), 1.45 – 1.32 (m, 2H). ^{13}C NMR (100 MHz, Acetone-d_6) δ 165.1, 147.4, 136.8, 124.2, 122.4, 122.3, 120.2, 119.7, 119.3, 111.9, 105.2, 37.2, 30.6, 25.7, 25.4. HRMS (MALDI): m/z calcd for $C_{17}H_{18}N_2O$ ([M + H]$^+$) 267.1492, Found 267.1487.

*1.6 5-(1H-indol-3-yl)-2-phenyloxazole (**3f**)*: Yellow solid; Yield: 47%. m.p.: 225.6-227.8 °C. ^{1}H NMR (400 MHz, Acetone-d_6) δ 10.80 (s, 1H), 8.19 – 8.12 (m, 2H), 8.04 – 7.99 (m, 1H), 7.95 (d, *J* = 2.4 Hz, 1H), 7.59 – 7.48 (m, 5H), 7.24 (qd, *J* = 7.2, 3.6 Hz, 2H). ^{13}C NMR (100 MHz,

Acetone-*d*$_6$) δ 158.8, 148.6, 136.9, 129.8, 128.9, 128.0, 125.7, 124.2, 123.3, 122.5, 121.1, 120.5, 119.7, 112.0, 104.8. HRMS (MALDI): m/z calcd for $C_{17}H_{12}N_2O$ ([M + H]$^+$) 261.1022, Found 261.1023.

1.7 5-(1H-indol-3-yl)-2-(2-(methylthio)ethyl)oxazole ***(3g)***: Yellow powder; Yield: 40%. m.p.: 141.2-143.2 °C. ^{1}H NMR (400 MHz, DMSO-*d*$_6$) δ 11.53 (s, 1H), 7.83 (d, *J* = 8.0 Hz, 1H), 7.73 (d, *J* = 2.4 Hz, 1H), 7.44 (d, *J* = 8.0 Hz, 1H), 7.30 (s, 1H), 7.15 (dt, *J* = 14.8, 7.2 Hz, 2H), 3.09 (t, *J* = 7.2 Hz, 2H), 2.90 (t, *J* = 7.2 Hz, 2H), 2.08 (s, 3H). ^{13}C NMR (100 MHz, Acetone-*d*$_6$) δ 160.4, 148.0, 136.8, 124.2, 122.6, 122.4, 120.3, 119.7, 119.5, 111.9, 105.0, 30.9, 28.3, 14.4. HRMS (MALDI): m/z calcd for $C_{14}H_{14}N_2SO$ ([M + H]$^+$) 259.0900, Found 259.0900.

1.8 4-chloro-5-(1H-indol-3-yl)oxazole ***(4a)***: White powder; Yield: 58%. m.p.:164.1-165.8 °C. ^{1}H NMR (400 MHz, DMSO-*d*$_6$) δ 11.80 (s, 1H), 8.50 (s, 1H), 7.94 – 7.87 (m, 2H), 7.51 (s, 1H), 7.27 – 7.20 (m, 1H), 7.20 – 7.14 (m, 1H). ^{13}C NMR (100 MHz, DMSO-*d*$_6$) δ 149.5 (d, *J* = 3.3 Hz), 142.8, 136.0, 124.8, 124.1, 122.6, 120.5, 120.1, 119.9, 112.3, 101.5. HRMS (MALDI): m/z calcd for $C_{11}H_7ClN_2O$ ([M + H]$^+$) 219.0320, Found 219.0320.

1.9 4-chloro-5-(1H-indol-3-yl)-2-methyloxazole ***(4b)***: White powder; Yield: 65%. m.p.: 183-185.5 °C. ^{1}H NMR (400 MHz, DMSO-*d*$_6$) δ 11.72 (s, 1H), 7.91 (d, *J* = 8.0 Hz, 1H), 7.84 (d, *J* = 2.8 Hz, 1H), 7.49 (d, *J* = 8.0 Hz, 1H), 7.18 (dt, *J* = 27.2, 7.2 Hz, 2H), 2.52 (s, 3H). ^{13}C NMR (100 MHz, Acetone-*d*$_6$) δ 158.2, 142.6, 136.3, 124.6, 123.7, 122.6, 120.7, 120.4, 111.9, 111.8, 102.9, 13.2. HRMS (MALDI): m/z calcd for $C_{12}H_9ClN_2O$ ([M + H]$^+$) 233.0476, Found 233.0478.

1.10 4-chloro-2-ethyl-5-(1H-indol-3-yl)oxazole ***(4c)***: Yellow powder; Yield: 57%. m.p.: 135.4-138.1 °C. ^{1}H NMR (400 MHz, Acetone-*d*$_6$) δ 10.83 (s, 1H), 8.02 (d, *J* = 8.0 Hz, 1H), 7.90 (s, 1H), 7.53 (d, *J* = 8.0 Hz, 1H), 7.28 – 7.15 (m, 2H), 2.89 (q, *J* = 7.6 Hz, 2H), 1.39 (t, *J* = 5.6 Hz, 3H). ^{13}C NMR (100 MHz, Acetone-*d*$_6$) δ 162.4, 142.5, 136.2, 124.6, 123.6, 122.6, 120.7, 120.4, 120.4, 111.9, 102.9, 21.4, 10.4. HRMS (MALDI): m/z calcd for $C_{13}H_{11}ClN_2O$ ([M + H]$^+$) 247.0633, Found 247.0634.

1.11 4-chloro-5-(1H-indol-3-yl)-2-isobutyloxazole ***(4d)***: Light yellow powder; Yield: 80%. m.p.: 106-107.7 °C. ^{1}H NMR (400 MHz, Acetone-*d*$_6$) δ 10.82 (s, 1H), 8.02 (d, *J* = 8.0 Hz, 1H), 8.02 (d, *J* = 8.0 Hz, 1H), 7.91 (s, 1H), 7.21 (dt, *J* = 24.0, 7.2 Hz, 2H), 2.75 (d, *J* = 7.2 Hz, 2H), 2.30 – 2.16 (m, 1H), 1.05 (d, *J* = 6.8 Hz, 6H). ^{13}C NMR (100 MHz, Acetone-*d*$_6$) δ 160.8, 142.6, 136.3, 124.6, 123.8, 122.6, 120.7, 120.5, 120.3, 111.9, 103.0, 36.7, 27.4, 21.7. HRMS (MALDI): m/z calcd for $C_{15}H_{15}ClN_2O$ ([M + H]$^+$) 275.0946, Found 275.0945.

1.12 4-chloro-2-cyclohexyl-5-(1H-indol-3-yl)oxazole ***(4e)***: Yellow powder; Yield: 62%. m.p.: 189.8-192.1 °C. ^{1}H NMR (400 MHz, Acetone-*d*$_6$) δ 10.82 (s, 1H), 8.03 (d, *J* = 8.0 Hz, 1H), 7.91 (d, *J* = 2.8 Hz, 1H), 7.54 (d, *J* = 8.0 Hz, 1H), 7.31 – 7.15 (m, 2H), 2.94 (tt, *J* = 11.2, 3.6 Hz, 1H), 2.17 (dd, *J* = 13.2, 3.2 Hz, 2H), 2.06 (dt, *J* = 4.4, 2.4 Hz, 2H), 1.90 – 1.81 (m, 2H), 1.74 – 1.66 (m, 2H), 1.54 – 1.42 (m, 2H). ^{13}C NMR (100 MHz, Acetone-*d*$_6$) δ 164.4, 142.2, 136.3, 124.6, 123.7, 122.6, 120.5, 120.4, 111.9, 103.0, 100.0, 37.3, 30.3, 25.6, 25.2. HRMS (MALDI): m/z calcd for $C_{17}H_{17}ClN_2O$ ([M + H]$^+$) 301.1102, Found 301.1104.

1.13 4-chloro-5-(1H-indol-3-yl)-2-phenyloxazole ***(4f)***: Light yellow powder; Yield: 57%. m.p.: 191.9-194 ºC. ^{1}H NMR (400 MHz, Acetone-d_6) δ 10.94 (s, 1H), 8.15 (d, J = 7.6 Hz, 2H), 8.05 (s, 1H), 7.68 – 7.43 (m, 5H), 7.33 – 7.23 (m, 2H). ^{13}C NMR (100 MHz, Acetone-d_6) δ 157.5, 143.5, 136.2, 130.5, 129.1, 127.0, 125.9, 125.7, 124.3, 122.8, 122.4, 120.8, 120.4, 112.0, 102.8. HRMS (MALDI): m/z calcd for $C_{17}H_{11}ClN_2O$ ([M + H]$^+$) 295.0633, Found 295.0640.

1.14 4-bromo-5-(1H-indol-3-yl)oxazole ***(5a)***: Brown powder; Yield: 62%. m.p.: 153.8-155.2 ºC. ^{1}H NMR (400 MHz, DMSO-d_6) δ 11.81 (s, 1H), 8.52 (s, 1H), 8.00 (d, J = 2.8 Hz, 1H), 7.94 (d, J = 8.0 Hz, 1H), 7.54 (d, J = 8.0 Hz, 1H), 7.27 – 7.23 (m, 1H), 7.21 – 7.16 (m, 1H). ^{13}C NMR (100 MHz, DMSO-d_6) δ 150.5, 145.2, 136.0, 124.9, 124.3, 122.6, 120.6, 120.0, 112.4, 107.2, 101.7. HRMS (MALDI): m/z calcd for $C_{11}H_7BrN_2O$ ([M + H]$^+$) 262.9815, Found 262.9815.

1.15 4-bromo-5-(1H-indol-3-yl)-2-methyloxazole ***(5b)***: Yellow powder; Yield: 76%. m.p.: 202.7-204.8 ºC. ^{1}H NMR (400 MHz, DMSO-d_6) δ 11.71 (s, 1H), 7.91 (t, J = 5.6 Hz, 2H), 7.50 (d, J = 8.0 Hz, 1H), 7.22 (t, J = 7.6 Hz, 1H), 7.15 (t, J = 7.6 Hz, 1H), 2.54 (s, 3H). ^{13}C NMR (100 MHz, Acetone-d_6) δ 158.5, 147.8, 136.8, 124.1, 122.4, 122.3, 120.2, 119.6, 119.6, 111.9, 105.0, 12.9. HRMS (MALDI): m/z calcd for $C_{12}H_9BrN_2O$ ([M + H]$^+$) 276.9971, Found 276.9968.

1.16 4-bromo-2-ethyl-5-(1H-indol-3-yl)oxazole ***(5c)***: Yellow powder; Yield: 60%. m.p.: 137.7-139.4 ºC. ^{1}H NMR (400 MHz, Acetone-d_6) δ 10.82 (s, 1H), 8.03 (d, J = 8.0 Hz, 1H), 8.00 (d, J = 2.8 Hz, 1H), 7.53 (d, J = 8.0 Hz, 1H), 7.27 – 7.15 (m, 2H), 2.94 – 2.87 (q, 2H), 1.38 (t, J = 7.6 Hz, 3H). ^{13}C NMR (100 MHz, Acetone-d_6) δ 163.3, 145.0, 136.3, 124.8, 124.0, 122.9, 122.6, 120.5, 111.9, 107.4, 103.1, 21.4, 10.5. HRMS (MALDI): m/z calcd for $C_{13}H_{11}BrN_2O$ ([M + H]$^+$) 291.0128, Found 291.0132.

1.17 4-bromo-5-(1H-indol-3-yl)-2-isobutyloxazole ***(5d)***: Yellow powder; Yield: 74%. m.p.: 105.2-106.6 ºC. ^{1}H NMR (400 MHz, Acetone-d_6) δ 10.84 (s, 1H), 8.03 (d, J = 8.0 Hz, 1H), 7.91 (s, 1H), 7.54 (d, J = 8.0 Hz, 1H), 7.27 – 7.15 (m, 2H), 2.76 (d, J = 7.2 Hz, 2H), 2.30 – 2.17 (m, 1H), 1.06 (d, J = 4.4 Hz, 6H). ^{13}C NMR (100 MHz, Acetone-d_6) δ 160.8, 142.6, 136.3, 124.6, 123.8, 123.6, 122.6, 120.5, 120.3, 111.9, 103.0, 36.7, 27.4, 21.7. HRMS (MALDI): m/z calcd for $C_{15}H_{15}BrN_2O$ ([M + H]$^+$) 319.0441, Found 319.0448.

1.18 4-bromo-2-cyclohexyl-5-(1H-indol-3-yl)oxazole ***(5e)***: Yellow powder; Yield: 76%. m.p.: 173.8-176.6 ºC. ^{1}H NMR (400 MHz, Acetone-d_6) δ 10.80 (s, 1H), 8.03 (d, J = 8.0 Hz, 1H), 8.00 (s, 1H), 7.53 (d, J = 8.0 Hz, 1H), 7.21 (dt, J = 14.8, 7.2 Hz, 2H), 3.03 – 2.89 (m, 1H), 2.16 (d, J = 11.2 Hz, 2H), 1.92 – 1.79 (m, 2H), 1.70 (dd, J = 18.8, 10.0 Hz, 2H), 1.66 – 1.49 (m, 2H), 1.36 (m, J = 27.2, 14.4, 6.4 Hz, 2H). ^{13}C NMR (100 MHz, Acetone-d_6) δ 165.3, 144.6, 136.2, 124.8, 123.9, 123.8, 122.6, 120.5, 111.9, 107.4, 103.2, 37.2, 30.3, 25.6, 25.3. HRMS (MALDI): m/z calcd for $C_{17}H_{17}BrN_2O$ ([M + H]$^+$) 345.0597, Found 345.0602.

1.19 4-bromo-5-(1H-indol-3-yl)-2-phenyloxazole ***(5f)***: Yellow powder; Yield: 70%. m.p.: 166.0-169.9 ºC. ^{1}H NMR (400 MHz, Acetone-d_6) δ 8.22 (dd, J = 6.0, 2.4 Hz, 1H), 8.16 (d, J = 1.6 Hz, 1H), 8.15 (d, J = 3.2 Hz, 2H), 7.83 – 7.35 (m, 5H), 7.28 – 7.26 (m, 1H). ^{13}C NMR (100 MHz,

Acetone-d_6) δ 158.6, 145.9, 136.2, 130.5, 129.1, 126.9, 125.7, 124.7, 124.5, 122.8, 120.8, 120.5, 112.0, 109.1, 102.9. HRMS (MALDI): m/z calcd for $C_{17}H_{11}BrN_2O$ ($[M + H]^+$) 339.0128, Found 339.0139.

1.20 4-bromo-5-(1H-indol-3-yl)-2-(2-(methylthio)ethyl)oxazole ***(5g)***: Light yellow powder; Yield: 53%. m.p.: 135.8-137.7 °C. ^{1}H NMR (400 MHz, Acetone-d_6) δ 10.84 (s, 1H), 8.06 (d, J = 8.0 Hz, 1H), 8.02 (s, 1H), 7.53 (t, J = 12.8 Hz, 1H), 7.22 (dt, J = 15.2, 7.2 Hz, 2H), 3.22 (t, J = 7.2 Hz, 2H), 3.02 (t, J = 7.2 Hz, 2H), 2.18 (s, 3H). ^{13}C NMR (100 MHz, Acetone-d_6) δ 160.9, 145.3, 136.2, 124.8, 124.1, 124.0, 122.6, 120.5, 111.9, 107.4, 103.0, 30.6, 28.3, 14.3. HRMS (MALDI): m/z calcd for $C_{14}H_{13}BrN_2SO$ ($[M + H]^+$) 337.0005, Found 337.0002.

1.21 5-(5-fluoro-1H-indol-3-yl)-2-methyloxazole ***(8a)***: Brown solid; Yield: 35%. m.p.: 218.2 – 219.2 °C. ^{1}H NMR (400 MHz, Acetone-d_6) *δ* 10.73 (s, 1H), 7.87 (dd, J = 8.8, 5.2 Hz, 1H), 7.73 (d, J = 2.4 Hz, 1H), 7.26 (dd, J = 10.0, 2.4 Hz, 1H), 7.23 (s, 1H), 7.00 (td, J = 9.2, 2.4 Hz, 1H), 2.48 (s, 3H). ^{13}C NMR (100 MHz, DMSO-d_6) *δ* 159.6 (d, J = 235.7 Hz), 158.9, 147.4, 136.8 (d, J = 12.9 Hz), 123.9 (d, J = 3.3 Hz), 121.0 (d, J = 9.9 Hz), 120.9, 120.0, 108.9 (d, J = 24.2 Hz), 104.6, 98.5 (d, J = 25.8 Hz), 14.0. HRMS (ESI): m/z calcd for $C_{12}H_9FN_2O$ ($[M + H]^+$) 217.0772, Found 217.0772.

1.22 5-(5-chloro-1H-indol-3-yl)-2-methyloxazole ***(8b)***: Yellow solid; Yield: 50%. m.p.: 213.1 – 214.0 °C. ^{1}H NMR (400 MHz, Acetone-d_6) *δ* 10.81 (s, 1H), 7.88 (d, J = 8.8 Hz, 1H), 7.76 (d, J = 2.8 Hz, 1H), 7.57 (d, J = 2.0 Hz, 1H), 7.24 (s, 1H), 7.18 (dd, J = 8.4, 2.0 Hz, 1H), 2.48 (s, 3H). ^{13}C NMR (100 MHz, DMSO-d_6) *δ* 159.0, 147.2, 137.2, 127.3, 124.4, 122.8, 121.3, 120.7, 120.1, 112.1, 104.7, 14.0. HRMS (ESI): m/z calcd for $C_{12}H_9ClN_2O$ ($[M + H]^+$) 233.0476, Found 233.0472.

1.23 5-(5-bromo-1H-indol-3-yl)-2-methyloxazole ***(8c)***: Yellow solid; Yield: 43%. m.p.: 219.3 – 220.1 °C. ^{1}H NMR (400 MHz, Acetone-d_6) *δ* 10.84 (s, 1H), 8.03 (d, J = 2.0 Hz, 1H), 7.77 (d, J = 2.8 Hz, 1H), 7.48 (d, J = 8.8 Hz, 1H), 7.33 (dd, J = 8.8, 2.0 Hz, 1H), 7.25 (s, 1H), 2.48 (s, 3H). ^{13}C NMR (100 MHz, DMSO-d_6) δ 158.6, 146.7, 135.2, 125.3, 124.6 (d, J = 25.6 Hz), 121.6, 119.8, 114.1, 112.8, 103.8, 30.7, 13.6. HRMS (ESI): m/z calcd for $C_{12}H_9BrN_2O$ ($[M + H]^+$) 276.9971, Found 276.9971.

1.24 2-methyl-5-(4-methyl-1H-indol-3-yl)oxazole ***(8d)***: Yellow solid; Yield: 32%. m.p.: 166.1 – 167.2 °C. ^{1}H NMR (400 MHz, Acetone-d_6) δ 10.75 (s, 1H), 7.53 (d, J = 2.8 Hz, 1H), 7.37 (d, J = 8.4 Hz, 1H), 7.11 (t, J = 7.6 Hz, 1H), 7.00 (s, 1H), 6.90 (d, J = 7.2 Hz, 1H), 2.49 (s, 3H), 2.47 (s, 3H). ^{13}C NMR (100 MHz, Acetone-d_6) δ 160.2, 147.4, 136.9, 130.0, 125.9, 125.3, 123.9, 122.3, 121.5, 109.7, 104.0, 19.5, 13.1. HRMS (ESI): m/z calcd for $C_{13}H_{12}N_2O$ ($[M + H]^+$) 213.1022, Found 213.1024.

1.25 5-(6-fluoro-1H-indol-3-yl)-2-methyloxazole ***(8e)***: Yellow solid; Yield: 61%. m.p.: 234.6–235.3 °C. ^{1}H NMR (400 MHz, DMSO-d_6) δ 11.59 (s, 1H), 7.83 (dd, J = 8.8, 5.6 Hz, 1H), 7.73 (d, J = 2.8 Hz, 1H), 7.32 (s, 1H), 7.25 (dd, J = 10.0, 2.4 Hz, 1H), 7.03–6.96 (m, 1H), 2.47 (s, 3H). ^{13}C NMR (100 MHz, DMSO-d_6) δ 159.3 (d, J = 235.7 Hz), 158.6, 147.1, 136.4 (d, J = 12.7 Hz), 123.6 (d, J = 2.9 Hz), 120.7 (d, J = 10.1 Hz), 120.5, 119.6, 108.6 (d, J = 24.4 Hz), 104.2, 98.2

(d, J = 25.6 Hz), 13.7. HRMS (ESI): m/z calcd for $C_{12}H_9FN_2O$ ($[M + H]^+$) 217.0772, Found 217.0770.

*1.26 5-(6-chloro-1H-indol-3-yl)-2-methyloxazole (**8f**)*: Yellow solid; Yield: 66%. m.p.: 206.7 – 208.1 °C. ^{1}H NMR (400 MHz, DMSO-d_6) δ 11.65 (s, 1H), 7.84 (d, J = 8.4 Hz, 1H), 7.77 (d, J = 2.8 Hz, 1H), 7.51 (d, J = 2.0 Hz, 1H), 7.31 (s, 1H), 7.19 – 7.11 (m, 1H), 2.47 (s, 3H). ^{13}C NMR (100 MHz, DMSO-d_6) δ 158.6, 146.9, 136.9, 126.9, 124.1, 122.4, 121.0, 120.4, 119.8, 111.8, 104.3, 13.7. HRMS (ESI): m/z calcd for $C_{12}H_9ClN_2O$ ($[M + H]^+$) 233.0476, Found 233.0475.

*1.27 2-methyl-5-(5-methyl-1H-indol-3-yl) oxazole (**8g**)*: Yellow solid; Yield: 39%. m.p.: 167.5–168.2 °C. ^{1}H NMR (400 MHz, DMSO-d_6) δ 11.42 (s, 1H), 7.68 (d, J = 2.8 Hz, 1H), 7.63 (s, 1H), 7.36 (d, J = 8.4 Hz, 1H), 7.29 (s, 1H), 7.02 (dd, J = 8.4, 1.2 Hz, 1H), 2.48 (s, 3H), 2.44 (s, 3H). ^{13}C NMR (100 MHz, DMSO-d_6) δ 158.2, 147.6, 134.8, 128.8, 123.9, 123.8, 123.0, 119.2, 119.2, 111.8, 103.5, 21.4, 13.7. HRMS (ESI): m/z calcd for $C_{13}H_{12}N_2O$ ($[M + H]^+$) 213.1022, Found 213.1015.

*1.28 5-(6-fluoro-1H-indol-3-yl)-2-ethyloxazole (**8h**)*: Yellow solid; Yield: 59%. m.p.: 232.8–234.1 °C. ^{1}H NMR (400 MHz, DMSO-d_6) δ 11.60 (s, 1H), 7.83 (dt, J = 8.4, 4.0 Hz, 1H), 7.74 (d, J = 2.8 Hz, 1H), 7.32 (s, 1H), 7.25 (dd, J = 10.0, 2.4 Hz, 1H), 7.00 (ddd, J = 9.6, 8.8, 2.4 Hz, 1H), 2.82 (q, J = 7.6 Hz, 2H), 1.30 (t, J = 8.8, 6.4 Hz, 3H). ^{13}C NMR (100 MHz, DMSO-d_6) δ 162.8, 159.3 (d, J = 235.7 Hz), 146.9, 136.5 (d, J = 12.8 Hz), 123.7 (d, J = 3.5 Hz), 120.7 (d, J = 10.2 Hz), 120.5, 119.5, 108.6 (d, J = 24.5 Hz), 104.3, 98.2 (d, J = 25.7 Hz), 21.1, 11.3. HRMS (ESI): m/z calcd for $C_{13}H_{11}FN_2O$ ($[M + H]^+$) 231.0928, Found 231.0926.

*1.29 5-(6-chloro-1H-indol-3-yl)-2-ethyloxazole (**8i**)*: Yellow solid; Yield: 58%. m.p.: 190.1–191.2 °C. ^{1}H NMR (400 MHz, DMSO-d_6) δ 11.65 (s, 1H), 7.84 (d, J = 8.4 Hz, 1H), 7.78 (d, J = 2.8 Hz, 1H), 7.50 (dd, J = 2.8, 1.2 Hz, 1H), 7.31 (s, 1H), 7.17 – 7.10 (m, 1H), 2.82 (q, J = 7.6 Hz, 2H), 1.32 – 1.28 (t, 3H). ^{13}C NMR (100 MHz, DMSO-d_6) δ 162.8, 146.7, 136.8, 126.9, 124.1, 122.4, 121.0, 120.4, 119.6, 111.8, 104.3, 21.1, 11.3. HRMS (ESI): m/z calcd for $C_{13}H_{11}ClN_2O$ ($[M + H]^+$) 247.0633, Found 247.0632.

*1.30 5-(6-bromo-1H-indol-3-yl)-2-ethyloxazole (**8j**)*: Yellow solid; Yield: 36%. m.p.: 191.2–192.8 °C. ^{1}H NMR (400 MHz, DMSO-d_6) δ 11.66 (s, 1H), 7.78 (dd, J = 10.8, 5.6 Hz, 2H), 7.65 (d, J = 1.6 Hz, 1H), 7.31 (s, 1H), 7.26 (dd, J = 8.4, 2.0 Hz, 1H), 2.82 (q, J = 7.6 Hz, 2H), 1.30 (t, J = 7.6 Hz, 3H). ^{13}C NMR (100 MHz, DMSO-d_6) δ 162.9, 146.7, 137.3, 124.0, 123.0, 122.7, 121.3, 119.6, 114.9, 114.7, 104.3, 21.1, 11.3. HRMS (ESI): m/z calcd for $C_{13}H_{11}BrN_2O$ ($[M + H]^+$) 291.0128, Found 291.0119.

*1.31 2-ethyl-5-(5-methyl-1H-indol-3-yl)oxazole (**8k**)*: Yellow solid; Yield: 45%. m.p.: 168.4 – 169.9 °C. ^{1}H NMR (400 MHz, DMSO-d_6) δ 11.40 (s, 1H), 7.66 (d, J = 2.8 Hz, 1H), 7.60 (dd, J = 1.6, 0.8 Hz, 1H), 7.36 – 7.30 (m, 1H), 7.25 (d, J = 9.6 Hz, 1H), 7.05 – 6.98 (m, 1H), 2.81 (q, J = 7.6 Hz, 2H), 2.43 (s, 3H), 1.30 (t, J = 7.6 Hz, 3H). ^{13}C NMR (100 MHz, DMSO-d_6) δ 162.5, 147.5, 134.8, 128.8, 123.9, 123.8, 123.0, 119.2, 119.0, 111.8, 103.6, 21.4, 21.1, 11.3. HRMS (ESI): m/z calcd for $C_{14}H_{14}N_2O$ ($[M + H]^+$) 227.1179, Found 227.1173.

*1.32 4-chloro-5-(5-fluoro-1H-indol-3-yl)-2-methyloxazole **(9a)***: White solid; Yield: 30%. m.p.: 193.3 – 194.1 °C. ^{1}H NMR (400 MHz, Acetone-d_6) δ 10.89 (s, 1H), 8.00 (dd, J = 8.8, 5.6 Hz, 1H), 7.94 – 7.86 (m, 1H), 7.28 (dd, J = 10.0, 2.4 Hz, 1H), 7.01 (ddd, J = 9.6, 8.8, 2.4 Hz, 1H), 2.54 (s, 3H). ^{13}C NMR (100 MHz, Acetone-d_6) δ 160.1 (d, J = 236.8 Hz), 158.3, 142.2, 136.3 (d, J = 12.8 Hz), 124.2 (d, J = 3.3 Hz), 121.5 (d, J = 10.2 Hz), 121.3, 121.0, 108.9 (d, J = 24.7 Hz), 103.1, 97.9 (d, J = 26.2 Hz), 13.1. HRMS (ESI): m/z calcd for $C_{12}H_8ClFN_2O$ ([M + H]$^+$) 251.0382, Found 251.0388.

*1.33 4-chloro-5-(5-chloro-1H-indol-3-yl)-2-methyloxazole **(9b)***: White solid; Yield: 35%. m.p.: 178.6-179.7 – 194.1 °C. ^{1}H NMR (400 MHz, Acetone-d_6) δ 10.95 (s, 1H), 7.99 (d, J = 8.8 Hz, 1H), 7.92 (d, J = 2.8 Hz, 1H), 7.63 – 7.54 (m, 1H), 7.23 – 7.15 (m, 1H), 2.54 (s, 3H). ^{13}C NMR (100 MHz, Acetone-d_6) δ 158.4, 142.0, 136.7, 128.0, 124.6, 124.4, 123.2, 121.6, 120.8, 111.7, 103.2, 13.2. HRMS (ESI): m/z calcd for $C_{12}H_8Cl_2N_2O$ ([M + H]$^+$) 267.0086, Found 267.0086.

*1.34 5-(5-bromo-1H-indol-3-yl)-4-chloro-2-methyloxazole **(9c)***: White solid; Yield: 42%. m.p.: 221.2 – 222.6 °C. ^{1}H NMR (400 MHz, Acetone-d_6) δ 11.02 (s, 1H), 8.16 (d, J = 2.0 Hz, 1H), 7.95 (d, J = 2.8 Hz, 1H), 7.52 (d, J = 8.8 Hz, 1H), 7.37 (dd, J = 8.8, 2.0 Hz, 1H), 2.56 (s, 3H). ^{13}C NMR (100 MHz, DMSO-d_6) δ 159.0, 141.9, 135.1, 126.1, 125.9, 125.4, 122.4, 120.7, 114.7, 113.3, 101.8, 14.3. HRMS (ESI): m/z calcd for $C_{12}H_8BrClN_2O$ ([M + H]$^+$) 310.9581, Found 310.9592.

*1.35 4-chloro-5-(6-fluoro-1H-indol-3-yl)-2-methyloxazole **(9d)***: White solid; Yield: 39%. m.p.: 203.1 – 204.9 °C. ^{1}H NMR (400 MHz, DMSO-d_6) δ 11.79 (s, 1H), 7.89 (dd, *J* = 19.6, 11.6 Hz, 2H), 7.29 (d, *J* = 9.6 Hz, 1H), 7.03 (t, *J* = 8.8 Hz, 1H), 2.52 (s, 3H). ^{13}C NMR (100 MHz, DMSO-d_6) δ 160.3, 158.5, 158.4, 141.9, 136.0 (d, *J* = 12.8 Hz), 124.8 (d, *J* = 3.4 Hz), 121.2 (d, *J* = 10.4 Hz), 120.6 (d, *J* = 83.7 Hz), 108.9 (d, *J* = 24.6 Hz), 101.9, 98.2 (d, *J* = 25.9 Hz), 13.9. HRMS (ESI): m/z calcd for $C_{12}H_8ClFN_2O$ ([M + H]$^+$) 251.0382, Found 251.0377.

*1.36 4-chloro-2-ethyl-5-(6-fluoro-1H-indol-3-yl)oxazole **(9e)***: White solid; Yield: 52%. m.p.: 189.2 – 191.2 °C. ^{1}H NMR (400 MHz, DMSO-d_6) δ 11.85 (s, 1H), 7.91 – 7.88 (m, 2H), 7.55 (dd, *J* = 2.0, 0.4 Hz, 1H), 7.18 (dd, *J* = 8.8, 2.0 Hz, 1H), 2.90 – 2.84 (q, 2H), 1.33 – 1.28 (t, 3H). ^{13}C NMR (100 MHz, DMSO-d_6) δ 162.5, 141.6, 136.4, 127.2, 125.2, 122.9, 121.4, 120.7, 120.4, 111.9, 102.1, 21.2, 10.8. HRMS (ESI): m/z calcd for $C_{13}H_{10}ClFN_2O$ ([M + H]$^+$) 265.0538, Found 265.0534.

*1.37 4-chloro-5-(6-chloro-1H-indol-3-yl)-2-methyloxazole **(9f)***: White solid; Yield: 33%. m.p.: 185.9 – 186.7 °C. ^{1}H NMR (400 MHz, DMSO-d_6) δ 11.86 (s, 1H), 7.90 (d, *J* = 8.4 Hz, 2H), 7.56 (s, 1H), 7.18 (d, *J* = 8.0 Hz, 1H), 2.53 (s, 3H). ^{13}C NMR (100 MHz, DMSO-d_6) δ 158.6, 141.7, 136.4, 127.2, 125.2, 122.8, 121.4, 120.7, 120.4, 111.9, 102.0, 13.9. HRMS (ESI): m/z calcd for $C_{12}H_8Cl_2N_2O$ ([M + H]$^+$) 267.0086, Found 267.0081.

*1.38 4-chloro-5-(6-chloro-1H-indol-3-yl)-2-ethyloxazole **(9g)***: White solid; Yield: 74%. m.p.: 195.5 – 196.9 °C. ^{1}H NMR (400 MHz, DMSO-d_6) δ 11.85 (s, 1H), 7.92 – 7.86 (m, 2H), 7.55 (dd, *J* = 2.0, 0.4 Hz, 1H), 7.23 – 7.15 (m, 1H), 2.87 (q, *J* = 7.6 Hz, 2H), 1.33 – 1.28 (t, 3H). ^{13}C NMR

(100 MHz, DMSO-*d*$_6$) δ 162.5, 141.6, 136.4, 127.2, 125.2, 122.9, 121.4, 120.7, 120.4, 111.9, 102.1, 21.2, 10.8. HRMS (ESI): m/z calcd for $C_{13}H_{10}Cl_2N_2O$ ([M + H]$^+$) 281.0243, Found 281.0244.

1.39 5-(6-bromo-1H-indol-3-yl)-4-chloro-2-ethyloxazole ***(9h)***: White solid; Yield: 60%. m.p.: 203.4–204.7 ºC. ^{1}H NMR (400 MHz, DMSO-*d*$_6$) δ 11.86 (s, 1H), 7.89 (d, *J* = 1.6 Hz, 1H), 7.84 (d, *J* = 8.8 Hz, 1H), 7.69 (d, *J* = 1.6 Hz, 1H), 7.29 (d, *J* = 8.4 Hz, 1H), 2.87 (q, *J* = 14.0, 7.2 Hz, 2H), 1.30 (t, *J* = 7.6 Hz, 3H). ^{13}C NMR (100 MHz, DMSO-*d*$_6$) δ 162.6, 141.5, 136.8, 125.1, 123.3, 123.1, 121.7, 120.4, 115.2, 114.9, 102.1, 21.2, 10.8. HRMS (ESI): m/z calcd for $C_{13}H_{10}BrClN_2O$ ([M + H]$^+$) 324.9738, Found 324.9736.

1.40 4-chloro-2-ethyl-5-(5-methyl-1H-indol-3-yl) oxazole ***(9i)***: White solid; Yield: 41%. m.p.: 191.5 – 192.9 ºC. ^{1}H NMR (400 MHz, DMSO-*d*$_6$) δ 11.61 (s, 1H), 7.78 (d, *J* = 2.8 Hz, 1H), 7.67 (d, *J* = 0.8 Hz, 1H), 7.38 (d, *J* = 8.4 Hz, 1H), 7.04 (dd, *J* = 8.4, 1.6 Hz, 1H), 2.87 (q, *J* = 7.6 Hz, 2H), 2.42 (s, 3H), 1.31 (t, 3H). ^{13}C NMR (100 MHz, DMSO-*d*$_6$) δ 162.2, 142.3, 134.3, 129.0, 124.4, 124.2, 124.1, 119.8, 119.5, 111.9, 101.3, 21.5, 21.2, 10.9. HRMS (ESI): m/z calcd for $C_{14}H_{13}ClN_2O$ ([M + H]$^+$) 261.0789, Found 261.0787.

1.41 4-bromo-5-(5-fluoro-1H-indol-3-yl)-2-methyloxazole ***(10a)***: White solid; Yield: 41%. m.p.: 178.6 – 179.8 ºC. ^{1}H NMR (400 MHz, Acetone-*d*$_6$) *δ* 10.89 (s, 1H), 8.04 – 7.98 (m, 2H), 7.29 (dd, J = 9.6, 2.4 Hz, 1H), 7.01 (td, J = 9.2, 2.4 Hz, 1H), 2.55 (s, 3H). ^{13}C NMR (100 MHz, Acetone-*d*$_6$) *δ* 160.1 (d, *J* = 236.8 Hz), 159.2, 144.6, 136.3 (d, *J* = 12.8 Hz), 124.5 (d, *J* = 3.3 Hz), 121.6 (d, *J* = 10.1 Hz), 121.5, 108.9 (d, *J* = 24.6 Hz), 107.7, 103.3, 97.9 (d, *J* = 26.2 Hz), 13.1. HRMS (ESI): m/z calcd for $C_{12}H_8BrFN_2O$ ([M + H]$^+$) 294.9877, Found 294.9880.

1.42 4-bromo-5-(5-chloro-1H-indol-3-yl)-2-methyloxazole ***(10b)***: White solid; Yield: 43%. m.p.: 178.6 – 179.7 ºC. ^{1}H NMR (400 MHz, Acetone-*d*$_6$) *δ* 10.94 (s, 1H), 8.04 – 7.98 (m, 2H), 7.59 (d, J = 2.0 Hz, 1H), 7.19 (dd, J = 8.4, 2.0 Hz, 1H), 2.55 (s, 3H). ^{13}C NMR (100 MHz, Acetone-*d*$_6$) *δ* 159.4, 144.5, 136.5, 128.0, 124.9, 124.7, 121.7, 120.8, 111.7, 107.8, 103.3, 13.1. HRMS (ESI): m/z calcd for $C_{12}H_8BrClN_2O$ ([M + H]$^+$) 310.9581, Found 310.9586.

1.43 4-bromo-5-(5-bromo-1H-indol-3-yl)-2-methyloxazole ***(10c)***: Yellow solid; Yield: 56%. m.p.: 183.5 – 185.2 ºC. ^{1}H NMR (400 MHz, Acetone-*d*$_6$) *δ* 11.02 (s, 1H), 8.16 (d, J = 2.0 Hz, 1H), 8.05 (d, J = 2.8 Hz, 1H), 7.52 (d, J = 8.8 Hz, 1H), 7.37 (dd, J = 8.8, 2.0 Hz, 1H), 2.58 (s, 3H). ^{13}C NMR (100 MHz, Acetone-*d*$_6$) δ 160.1, 145.2, 137.3 (d, *J* = 15.6 Hz), 128.7, 125.5 (d, *J* = 16.4 Hz), 124.1, 122.4, 121.5, 112.4 (d, *J* = 5.2 Hz), 108.5, 104.0 (d, *J* = 4.8 Hz), 13.8. HRMS (ESI): m/z calcd for $C_{12}H_8Br_2N_2O$ ([M + H]$^+$) 354.9076, Found 354.9079.

1.44 4-bromo-2-methyl-5-(4-methyl-1H-indol-3-yl)oxazole ***(10d)***: White solid; Yield: 49%. m.p.: 151.0–151.9 ºC. ^{1}H NMR (400 MHz, Acetone-*d*$_6$) *δ* 10.81 (s, 1H), 7.64 (d, J = 2.8 Hz, 1H), 7.38 (d, J = 8.4 Hz, 1H), 7.11 (t, J = 7.6 Hz, 1H), 6.91 (d, J = 7.2 Hz, 1H), 2.51 (s, 3H), 2.37 (s, 3H). ^{13}C NMR (100 MHz, Acetone-*d*$_6$) *δ* 160.5, 144.2, 136.6, 129.7, 127.3, 125.6, 122.5, 121.6, 113.5, 109.8, 101.3, 18.9, 13.3. HRMS (ESI): m/z calcd for $C_{13}H_{11}BrN_2O$ ([M + H]$^+$) 291.0128, Found 291.0123.

1.45 4-bromo-5-(6-fluoro-1H-indol-3-yl)-2-methyloxazole ***(10e)***: White solid; Yield: 66%. m.p.: 198.8–199.4 °C. ^{1}H NMR (400 MHz, DMSO-*d$_6$*) δ 11.77 (s, 1H), 7.93 – 7.87 (m, 2H), 7.29 (dd, *J* = 10.0, 2.4 Hz, 1H), 7.06 – 6.99 (m, 1H), 2.53 (s, 3H). ^{13}C NMR (100 MHz, DMSO-*d$_6$*) δ 159.4, 159.4 (d, *J* = 236.2 Hz), 144.3, 136.0 (d, *J* = 12.9 Hz), 124.9 (d, *J* = 3.4 Hz), 121.3 (d, *J* = 10.3 Hz), 121.1, 108.9 (d, *J* = 24.5 Hz), 107.4, 102.2, 98.2 (d, *J* = 25.8 Hz), 13.8. HRMS (ESI): m/z calcd for $C_{12}H_8BrFN_2O$ ([M + H]$^+$) 294.9877, Found 294.9877.

1.46 4-bromo-2-ethyl-5-(6-fluoro-1H-indol-3-yl) oxazole ***(10f)***: White solid; Yield: 37%. m.p.: 163.1–163.9 °C. ^{1}H NMR (400 MHz, DMSO-*d$_6$*) δ 11.84 (s, 1H), 7.95 (d, *J* = 2.8 Hz, 1H), 7.89 (d, *J* = 8.8 Hz, 1H), 7.55 (d, *J* = 1.6 Hz, 1H), 7.18 (dd, *J* = 8.8, 2.0 Hz, 1H), 2.88 (q, *J* = 7.6 Hz, 2H), 1.30 (t, *J* = 7.6 Hz, 3H). ^{13}C NMR (100 MHz, DMSO-*d$_6$*) δ 163.3, 159.4 (d, *J* = 236.1 Hz), 144.2, 136.0 (d, *J* = 12.8 Hz), 124.9 (d, *J* = 3.4 Hz), 121.3 (d, *J* = 10.3 Hz), 121.1, 109.0 (d, *J* = 24.6 Hz), 107.4, 102.2, 98.3 (d, *J* = 25.7 Hz), 21.2, 10.9. HRMS (ESI): m/z calcd for $C_{13}H_{10}BrFN_2O$ ([M + H]$^+$) 309.0033, Found 309.0027.

1.47 4-bromo-5-(6-chloro-1H-indol-3-yl)-2-methyloxazole ***(10g)***: White solid; Yield: 68%. m.p.: 186.1–187.5 °C. ^{1}H NMR (400 MHz, DMSO-*d$_6$*) δ 11.84 (s, 1H), 7.93 (dd, *J* = 24.4, 6.0 Hz, 2H), 7.55 (d, *J* = 2.4 Hz, 1H), 7.18 (dd, *J* = 8.8, 2.4 Hz, 1H), 2.53 (s, 3H). ^{13}C NMR (100 MHz, DMSO-*d$_6$*) δ 159.5, 144.1, 136.4, 127.2, 125.3, 123.0, 121.5, 120.7, 111.9, 107.6, 102.3, 13.8. HRMS (ESI): m/z calcd for $C_{12}H_8BrClN_2O$ ([M + H]$^+$) 310.9581, Found 310.9579.

1.48 4-bromo-5-(6-chloro-1H-indol-3-yl)-2-ethyloxazole ***(10h)***: White solid; Yield: 54%. m.p.: 176.2 – 177.4 °C. ^{1}H NMR (400 MHz, DMSO-*d$_6$*) δ 11.84 (s, 1H), 7.96 (d, *J* = 2.8 Hz, 1H), 7.89 (d, *J* = 8.8 Hz, 1H), 7.55 (d, *J* = 1.6 Hz, 1H), 7.18 (dd, *J* = 8.8, 2.0 Hz, 1H), 2.88 (q, *J* = 7.6 Hz, 2H), 1.31 (t, *J* = 7.6 Hz, 3H). ^{13}C NMR (100 MHz, DMSO-*d$_6$*) δ 163.5, 144.0, 136.4, 127.2, 125.3, 123.0, 121.5, 120.7, 111.9, 107.6, 102.3, 21.2, 10.9. HRMS (ESI): m/z calcd for $C_{13}H_{10}BrClN_2O$ ([M + H]$^+$) 324.9738, Found 324.9740.

1.49 4-bromo-5-(6-bromo-1H-indol-3-yl)-2-ethyloxazole ***(10i)***: White solid; Yield: 70%. m.p.: 172.5 – 174.4 °C. ^{1}H NMR (400 MHz, DMSO-*d$_6$*) δ 11.85 (s, 1H), 7.96 (d, *J* = 3.2 Hz, 1H), 7.85 (d, *J* = 9.2 Hz, 1H), 7.71 (d, *J* = 2.0 Hz, 1H), 7.30 (dd, *J* = 10.4, 2.0 Hz, 1H), 2.88 (q, *J* = 16.8, 8.0 Hz, 2H), 1.31 (t, *J* = 8.0 Hz, 3H). ^{13}C NMR (100 MHz, DMSO-*d$_6$*) δ 163.4, 144.0, 136.8, 125.2, 123.3, 123.3, 121.8, 115.2, 114.9, 107.6, 102.3, 21.2, 10.9. HRMS (ESI): m/z calcd for $C_{13}H_{10}Br_2N_2O$ ([M + H]$^+$) 368.9233, Found 368.9234.

2. ^{1}H-NMR

Compound 2

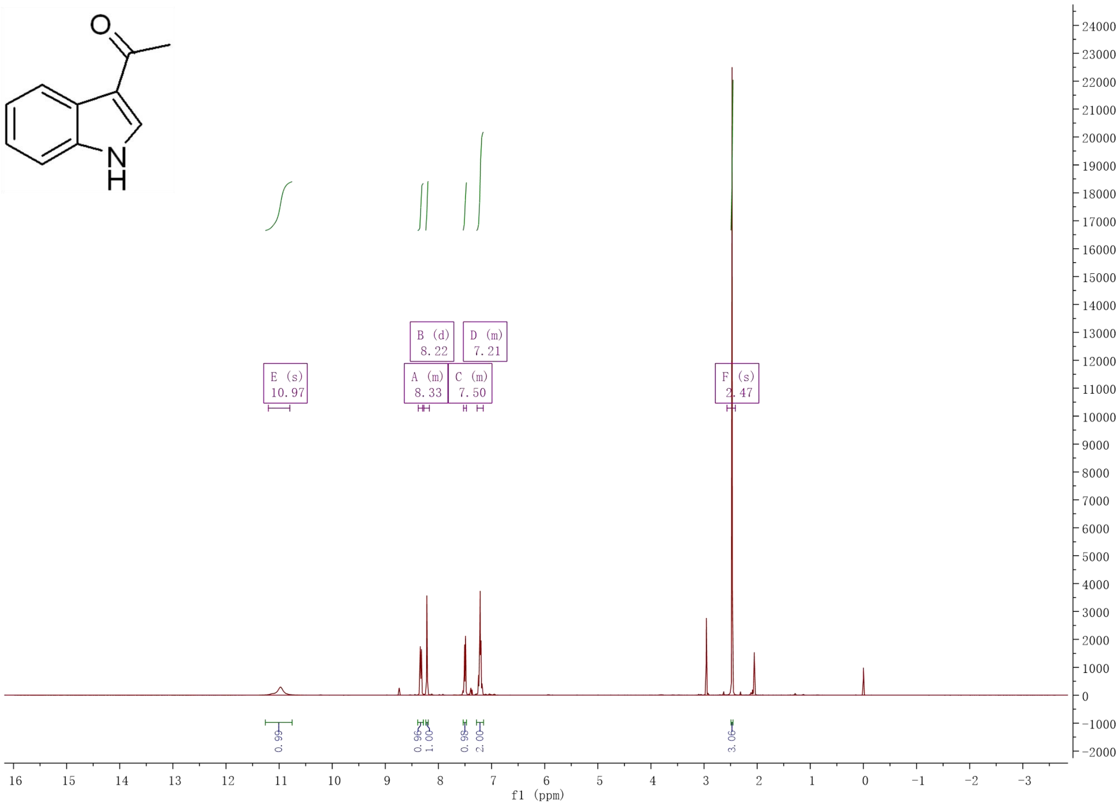

Compound 3a

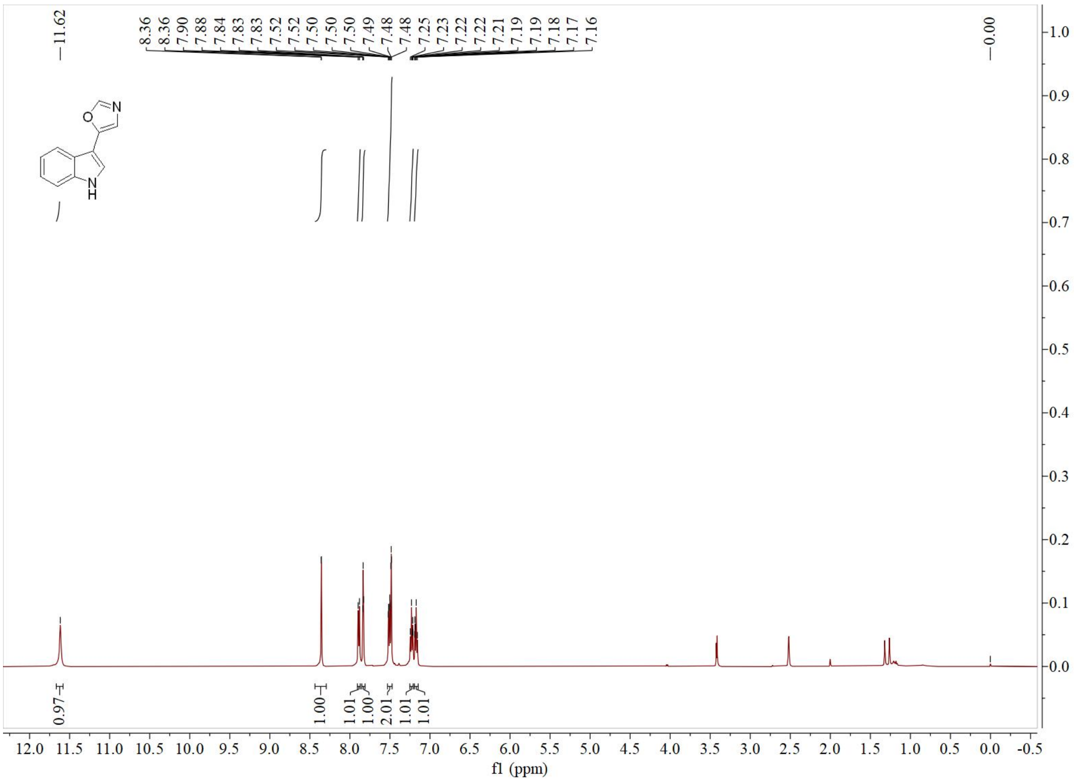

Compound 3b

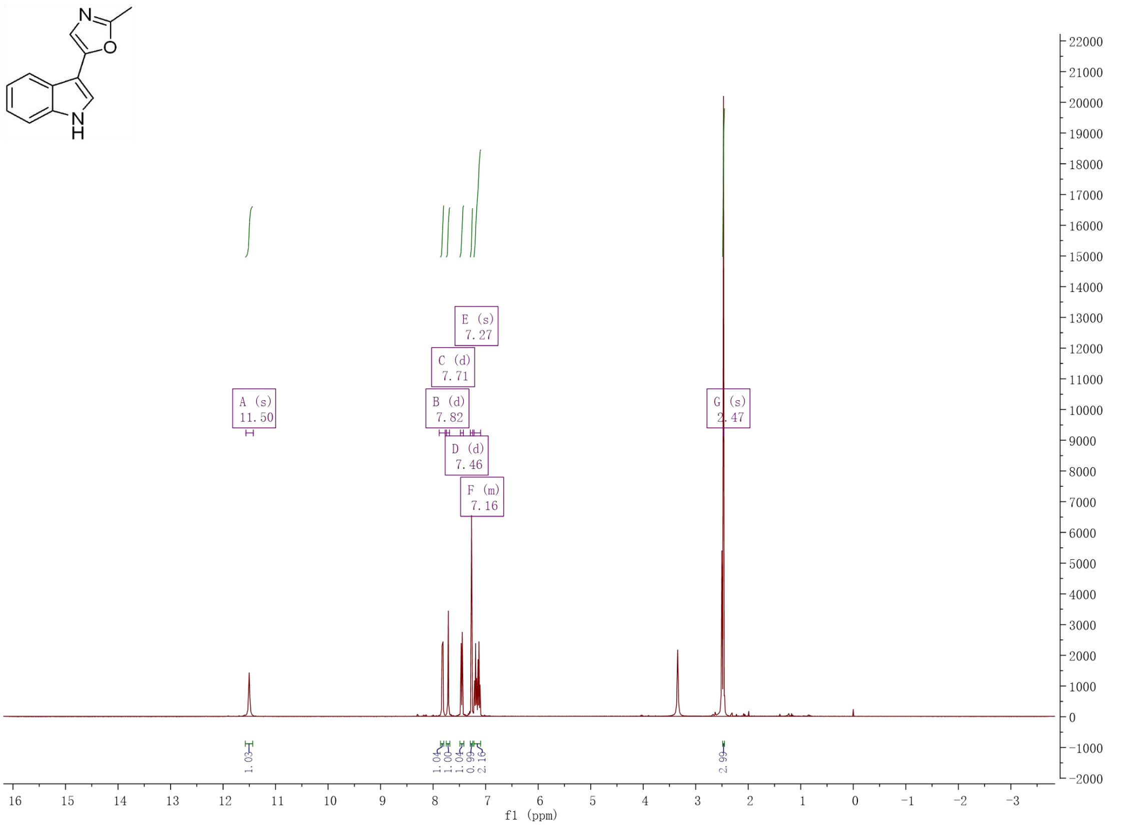

Compound 3c

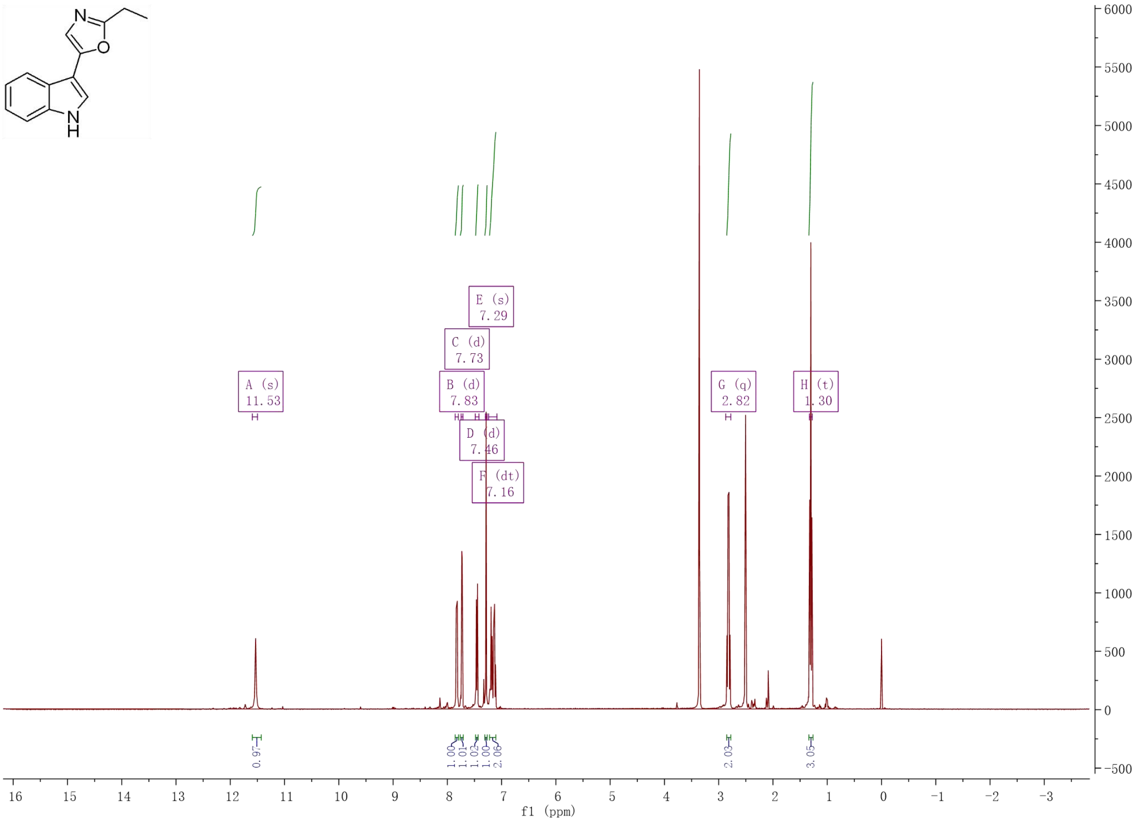

Compound 3d

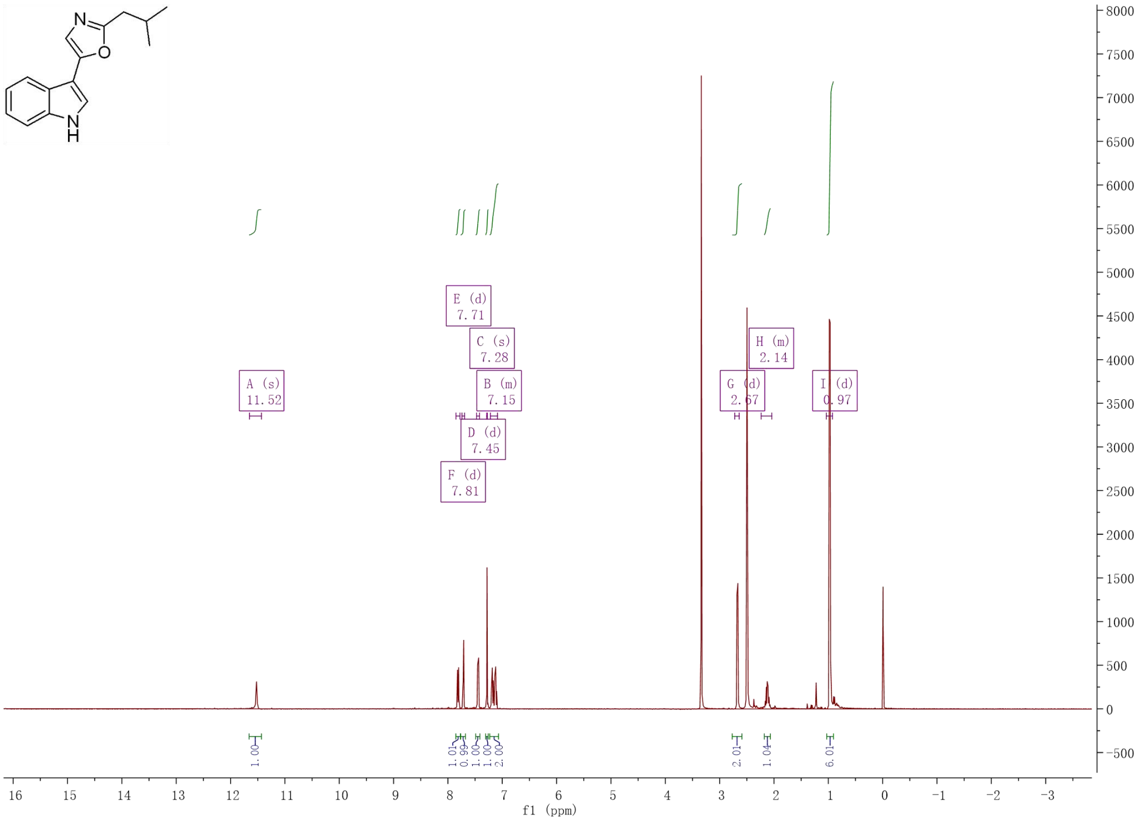

Compound 3e

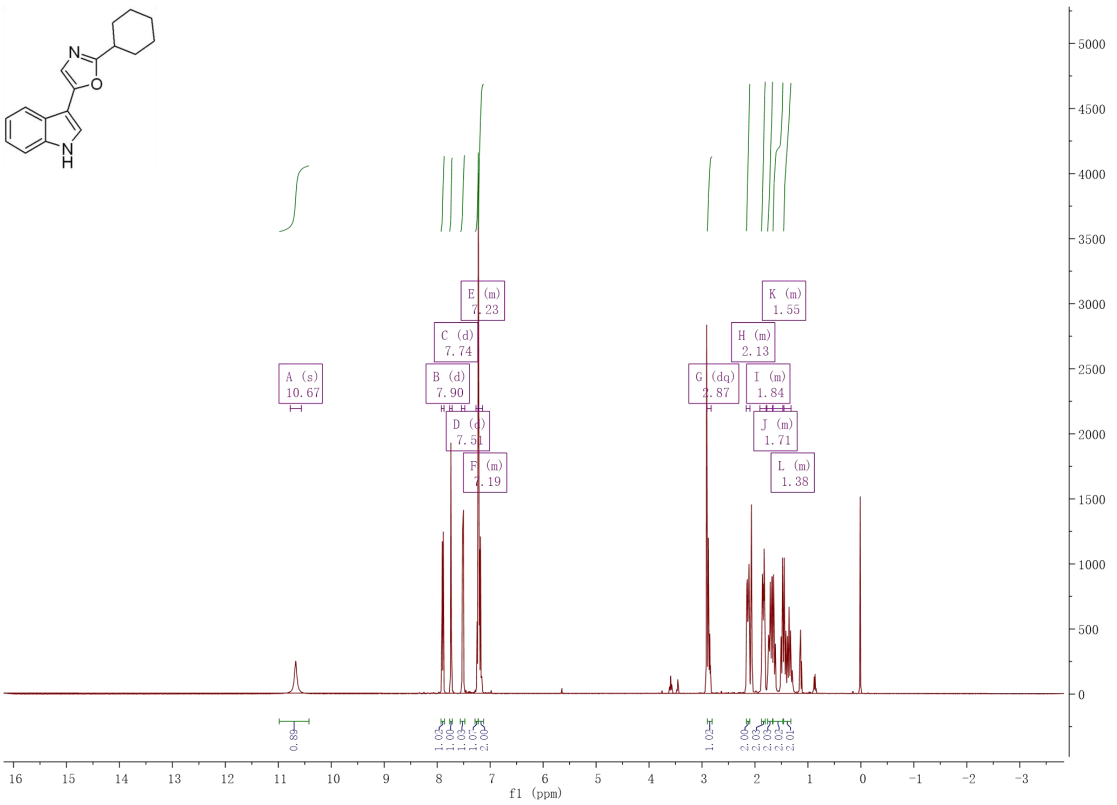

Compound 3f

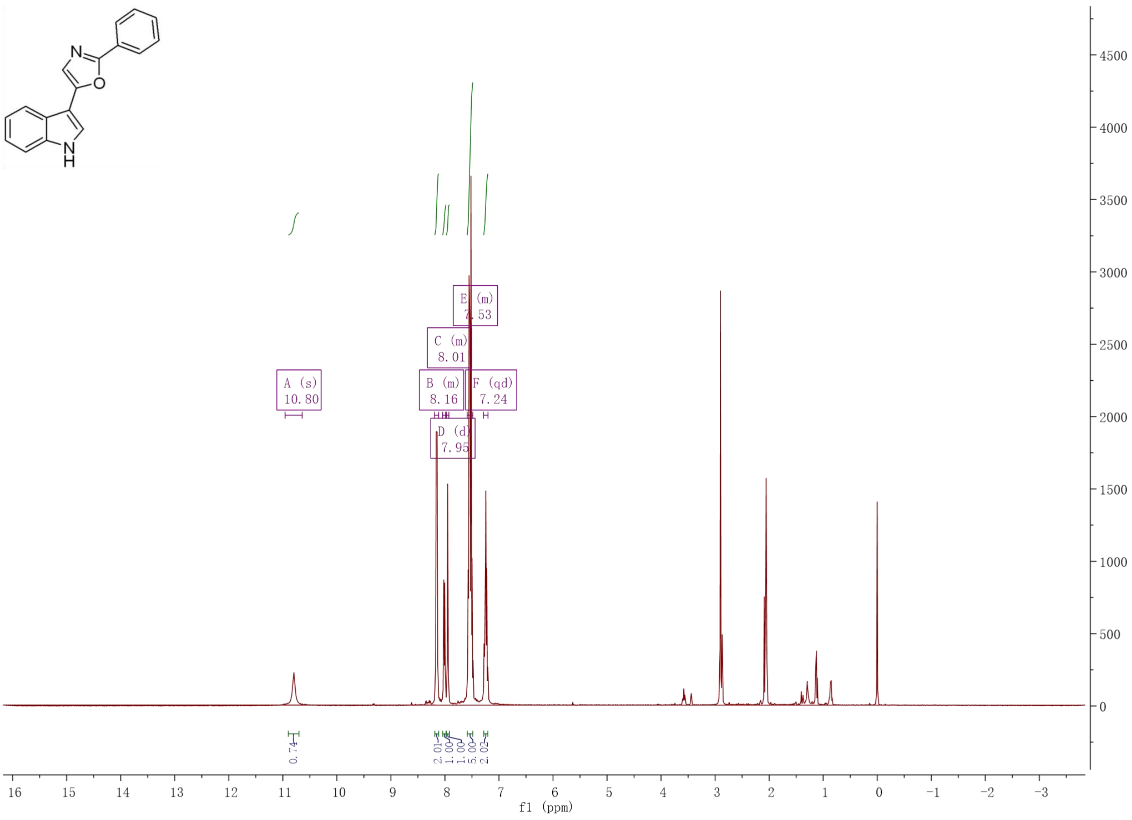

Compound 3g

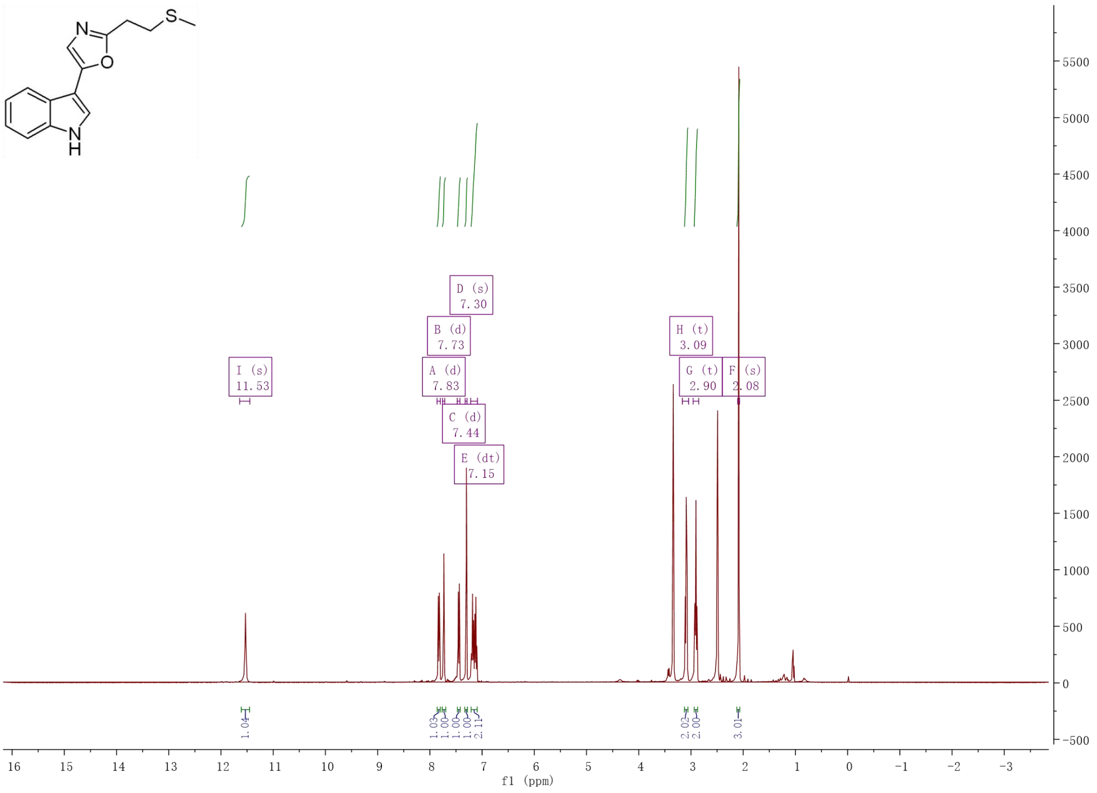

Compound 4a

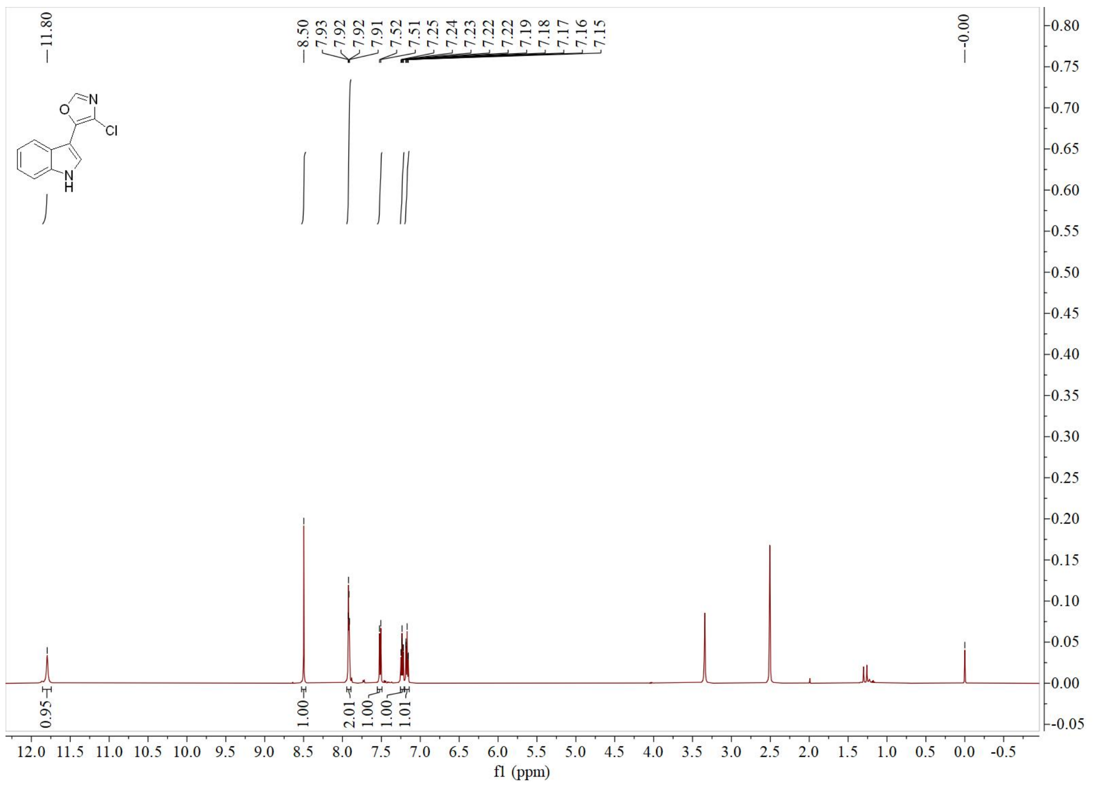

Compound 4b

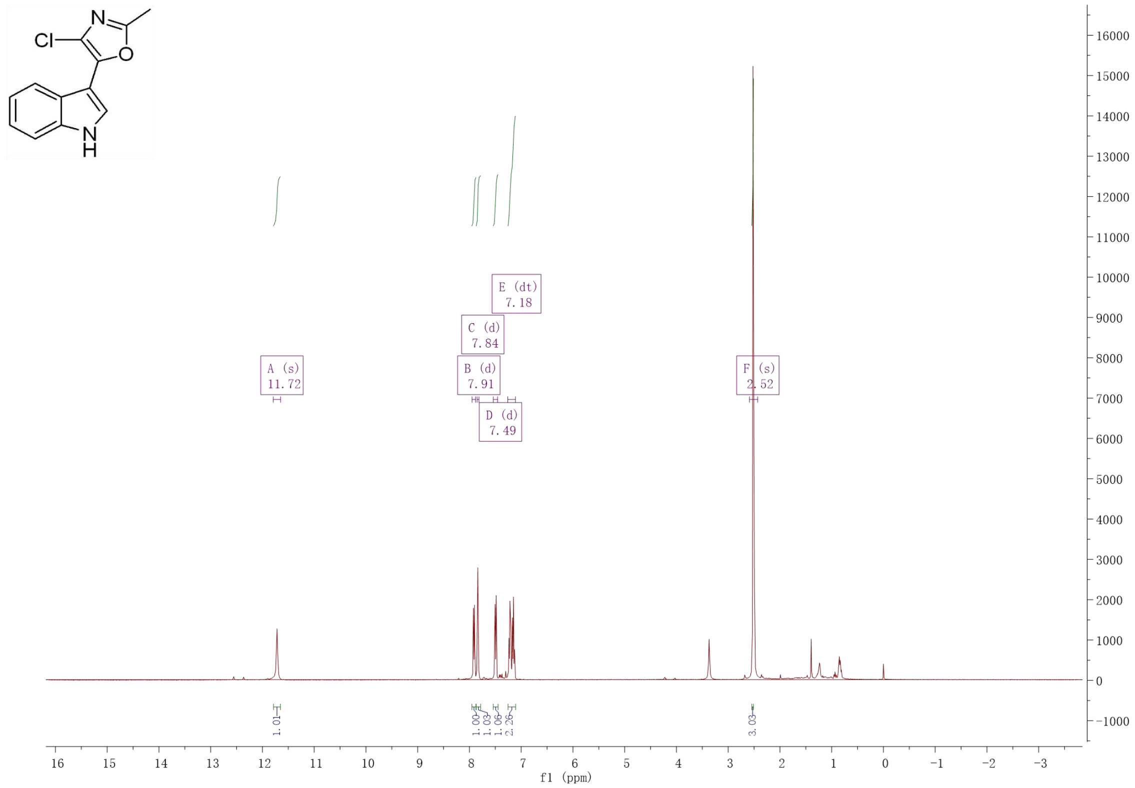

Compound 4c

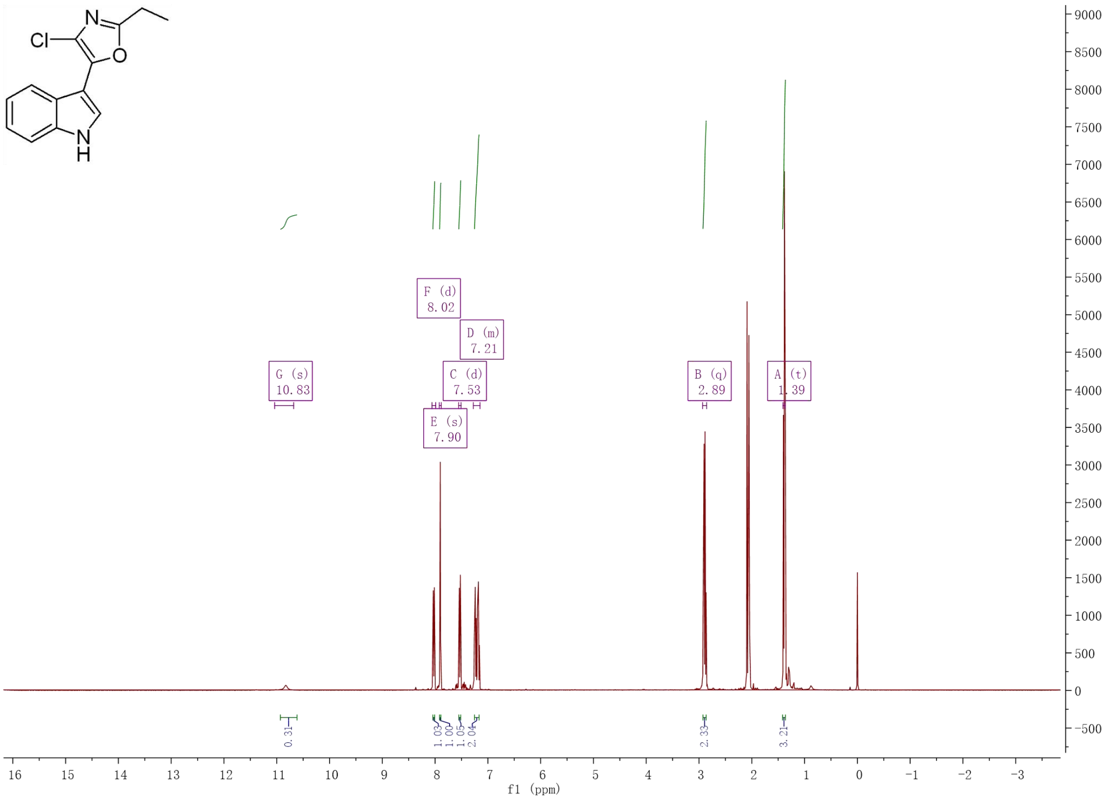

Compound 4d

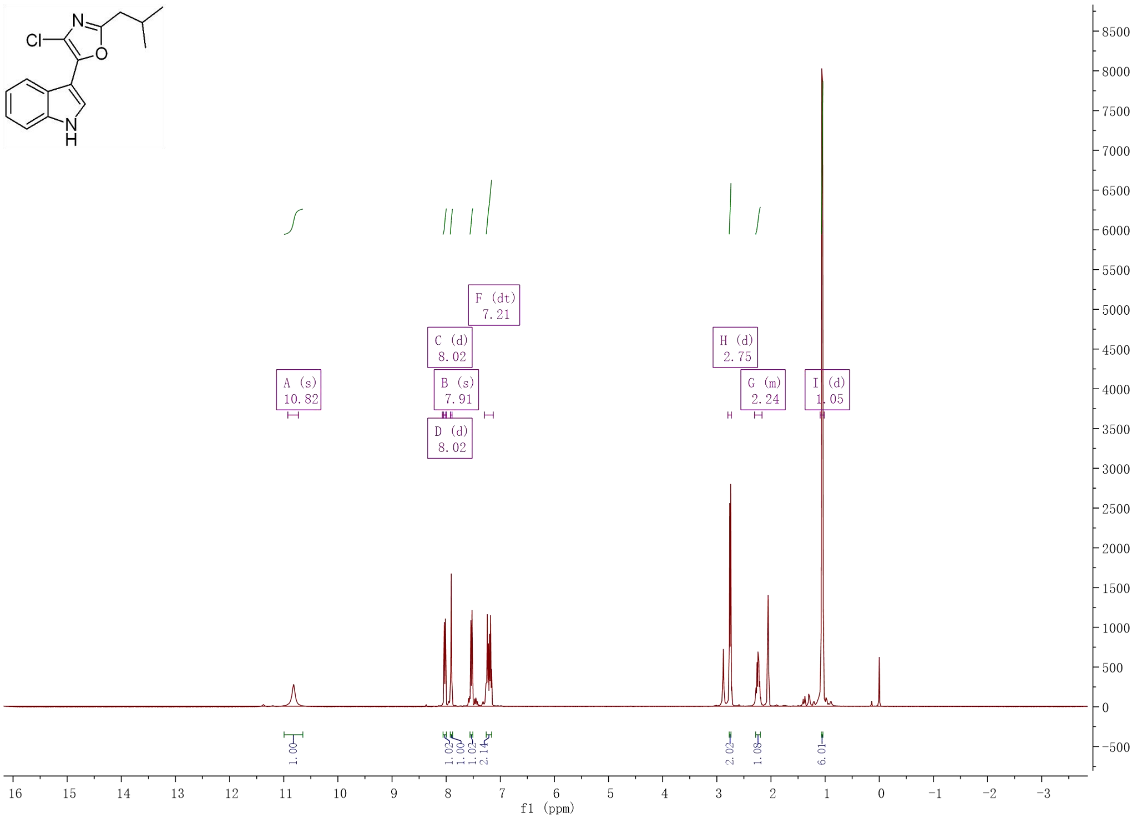

Compound 4e

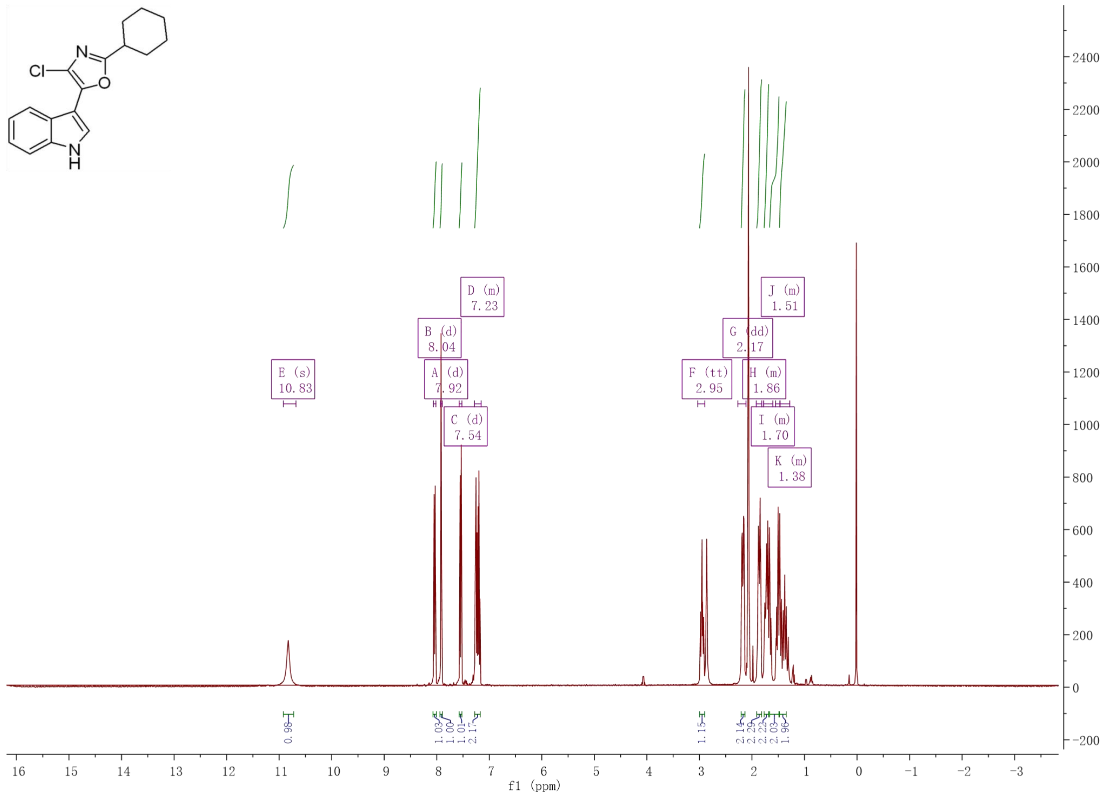

Compound 4f

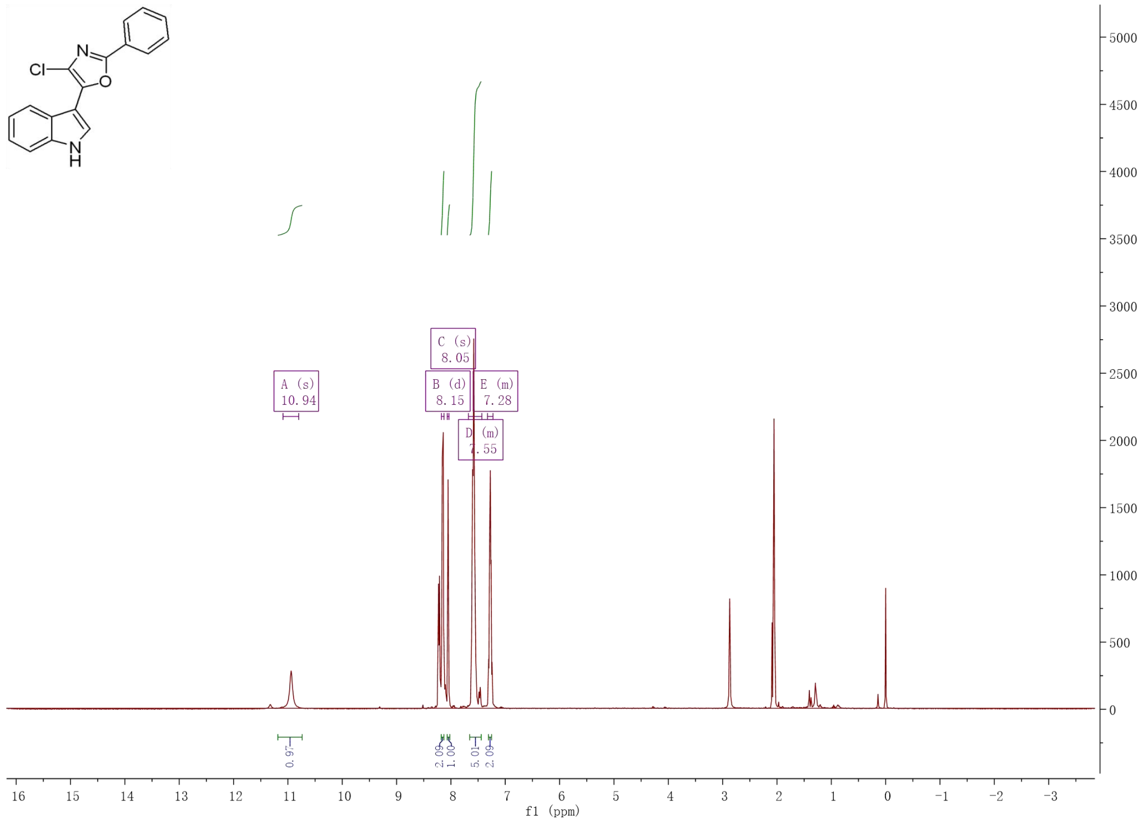

Compound 5a

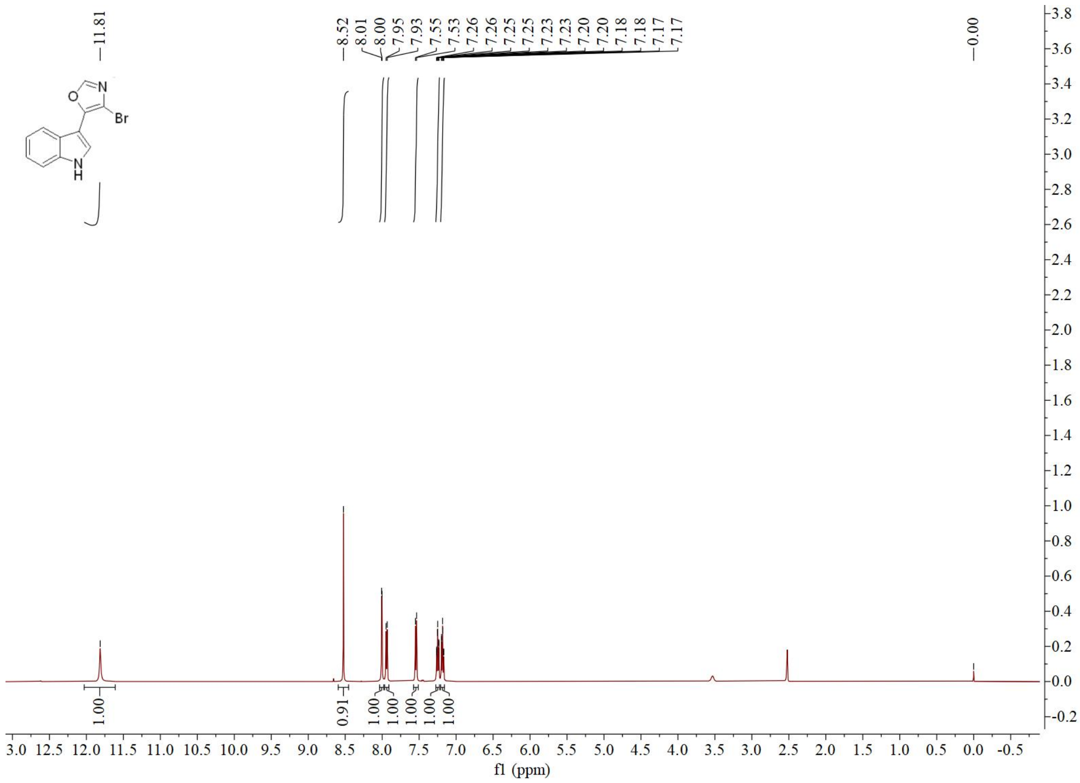

Compound 5b

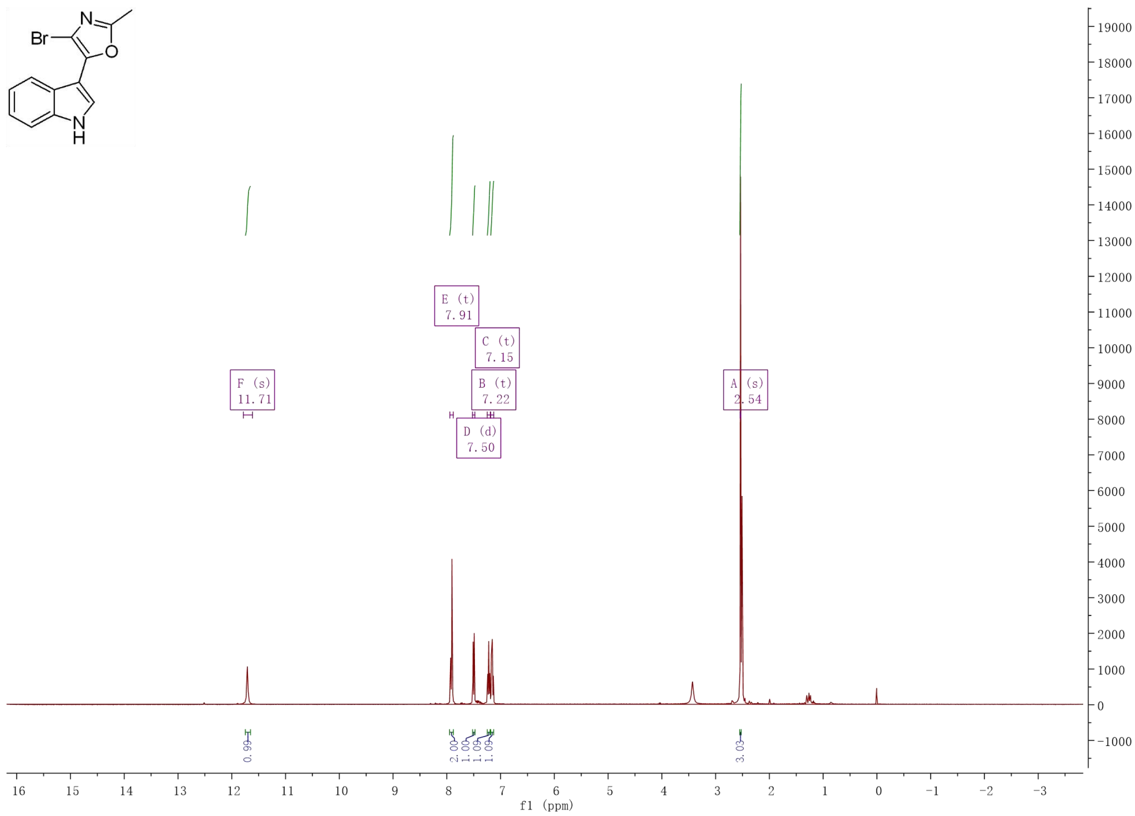

Compound 5c

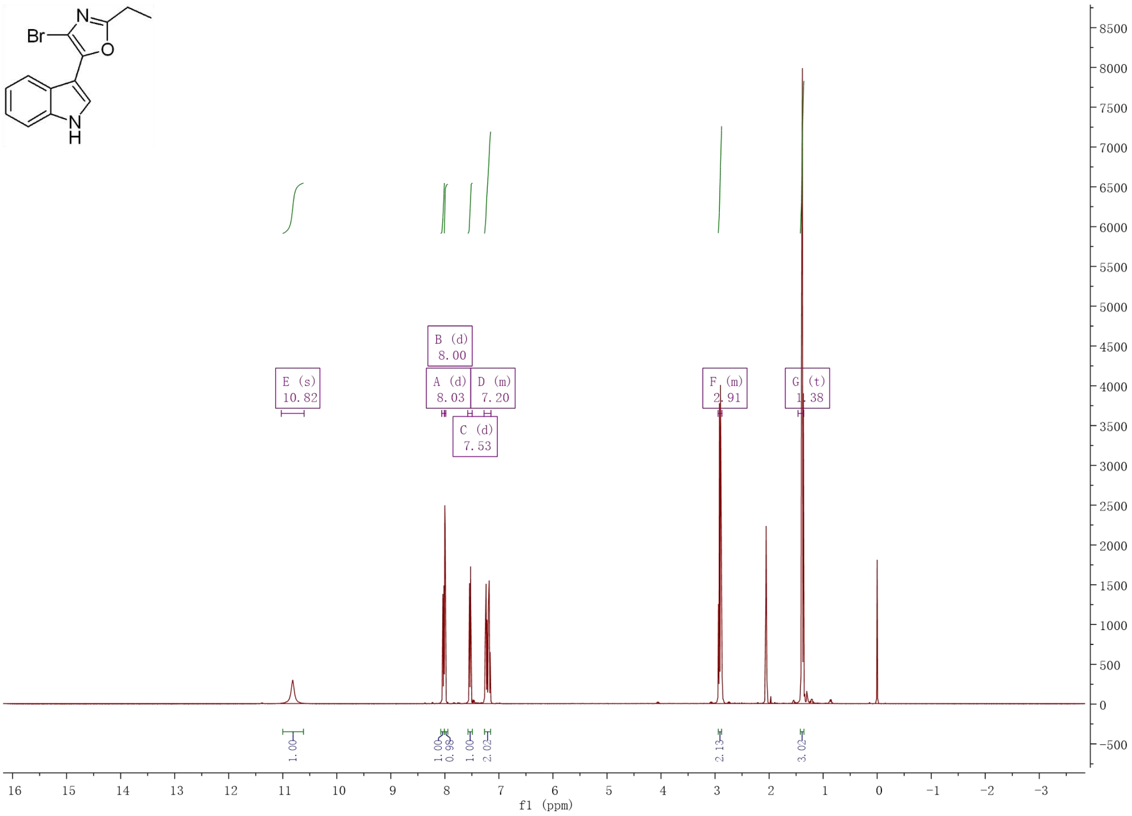

Compound 5d

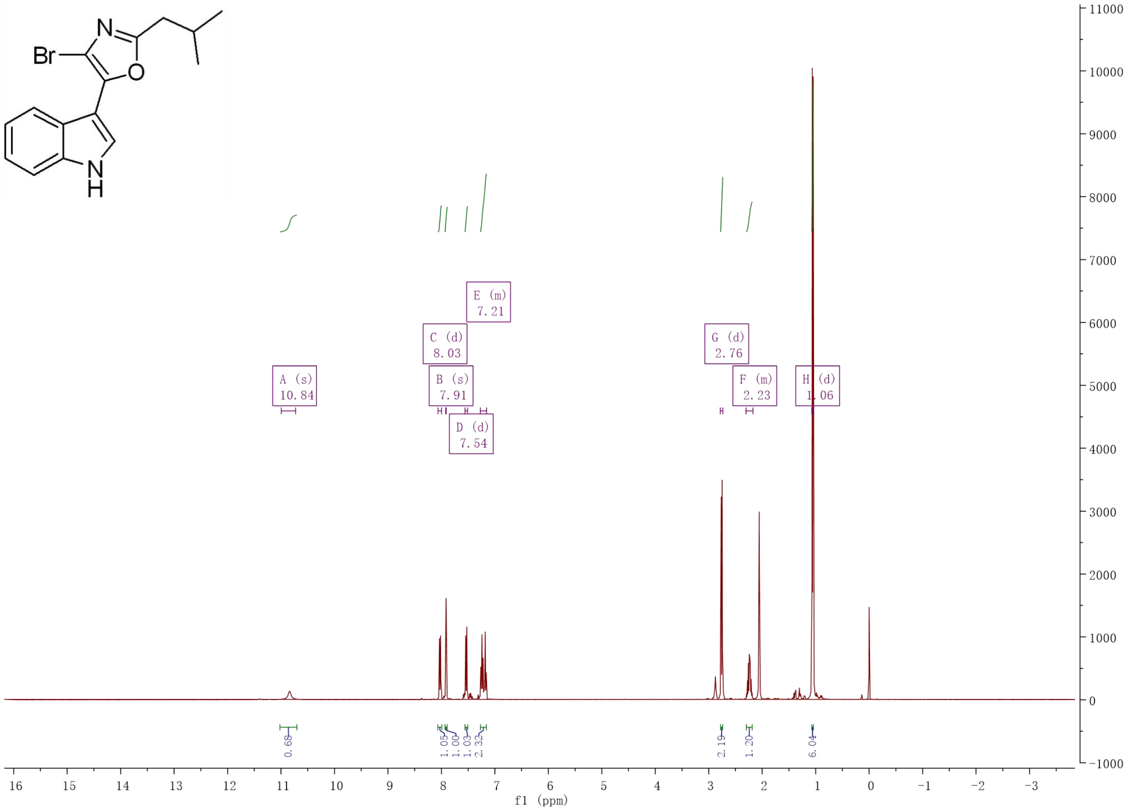

Compound 5e

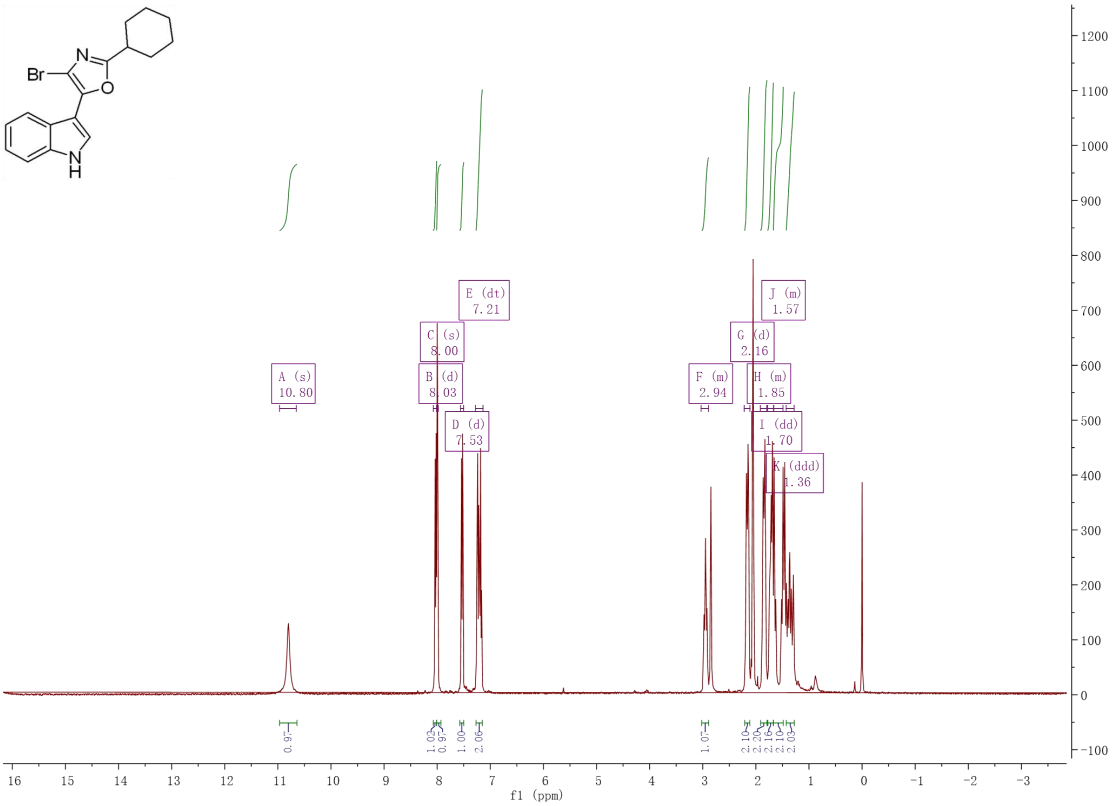

Compound 5f

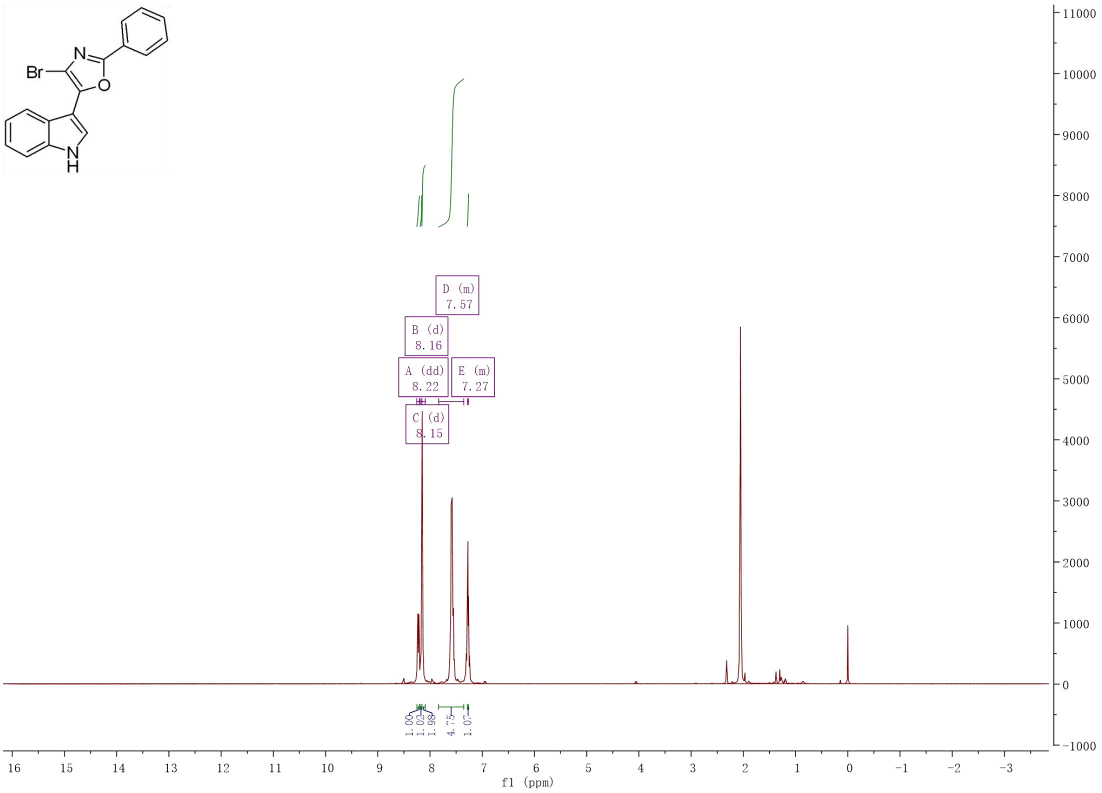

Compound 5g

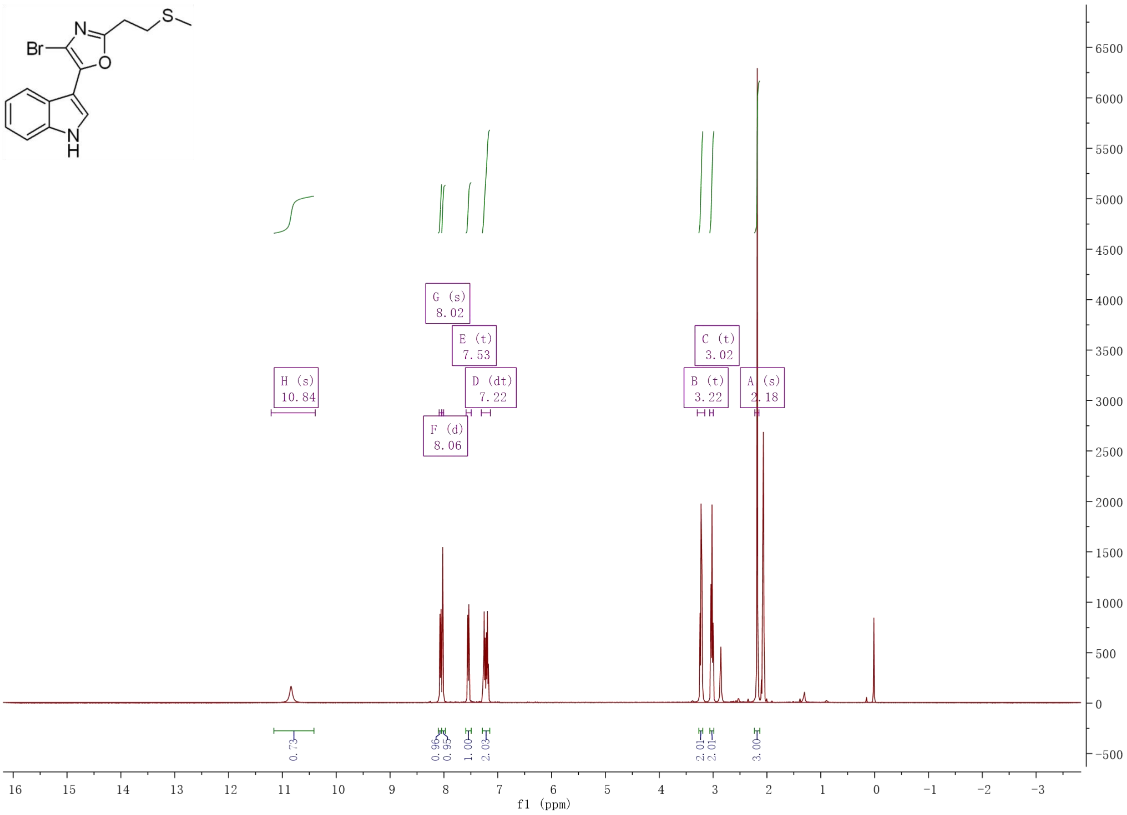

Compound 8a

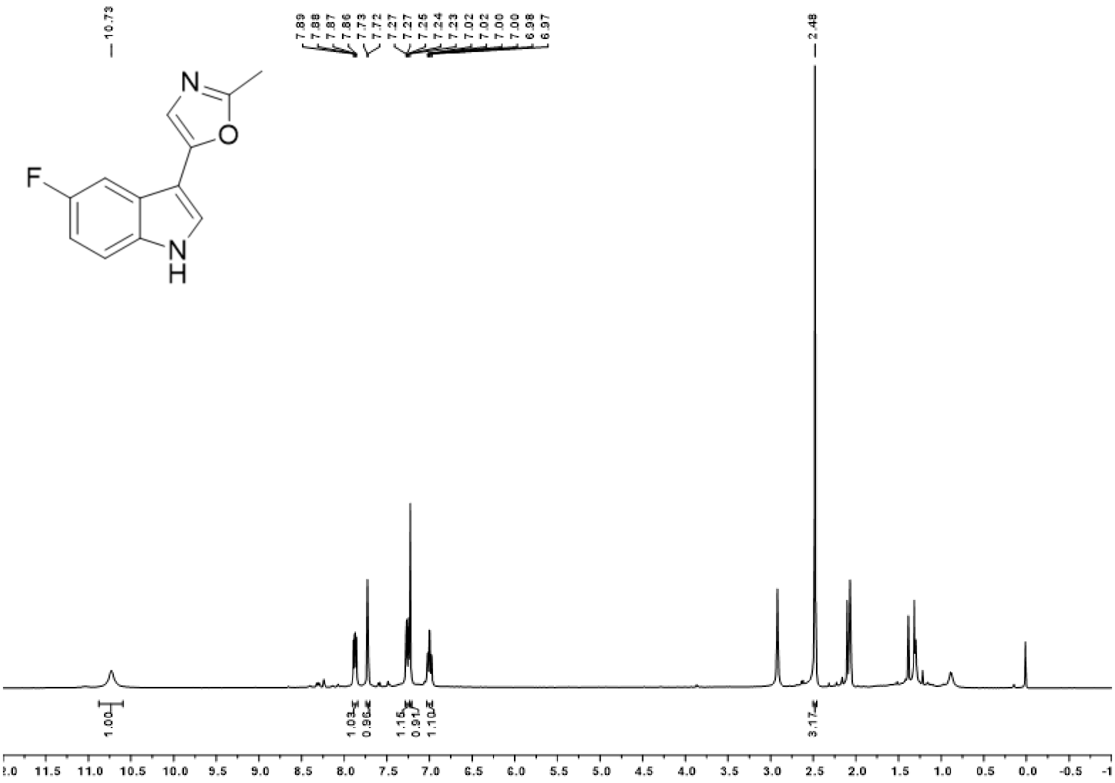

Compound 8b

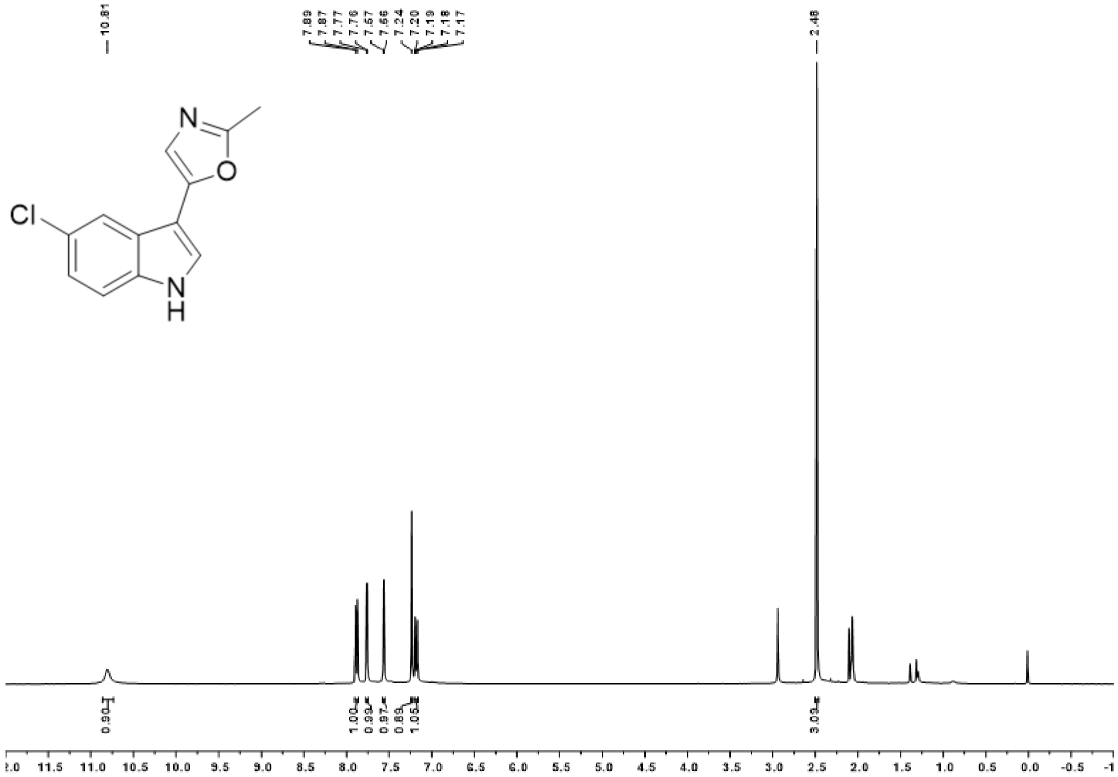

Compound 8c

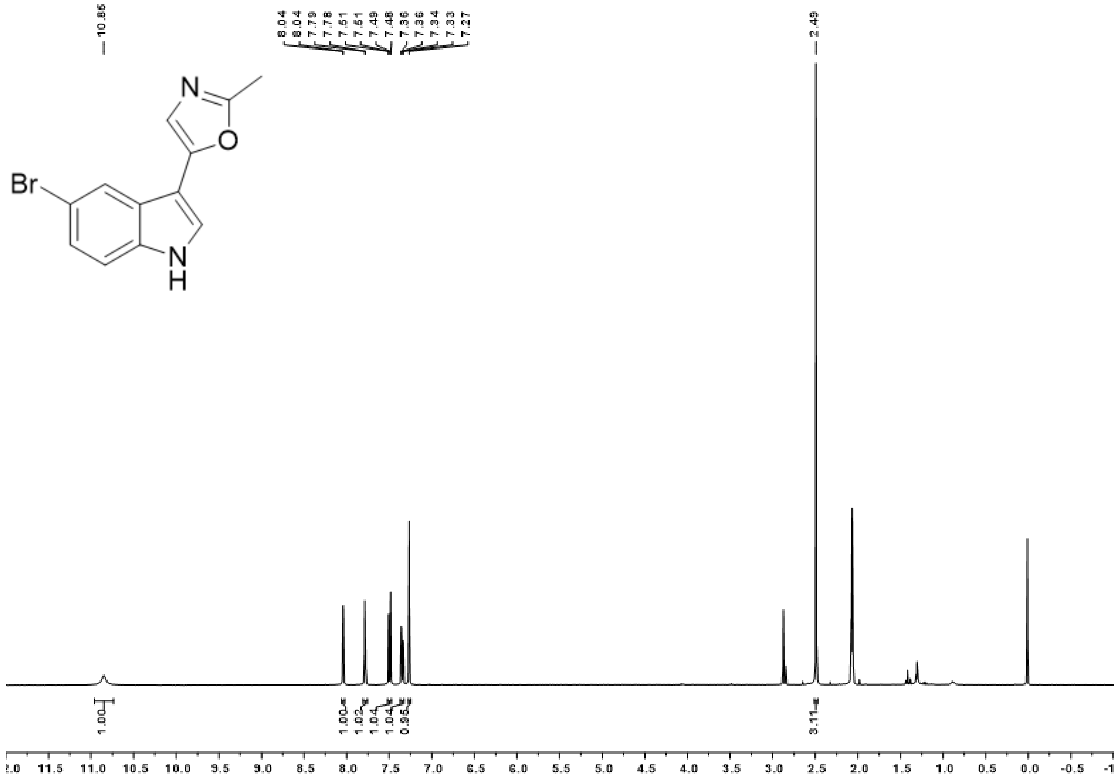

Compound 8d

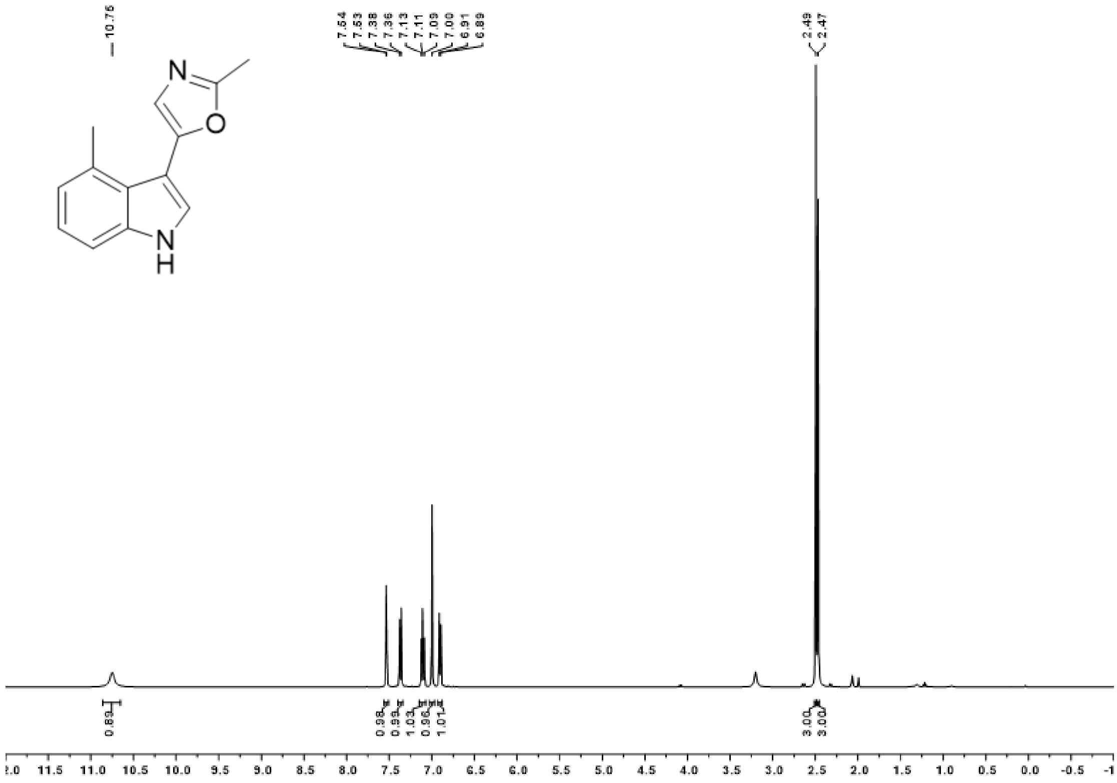

Compound 8e

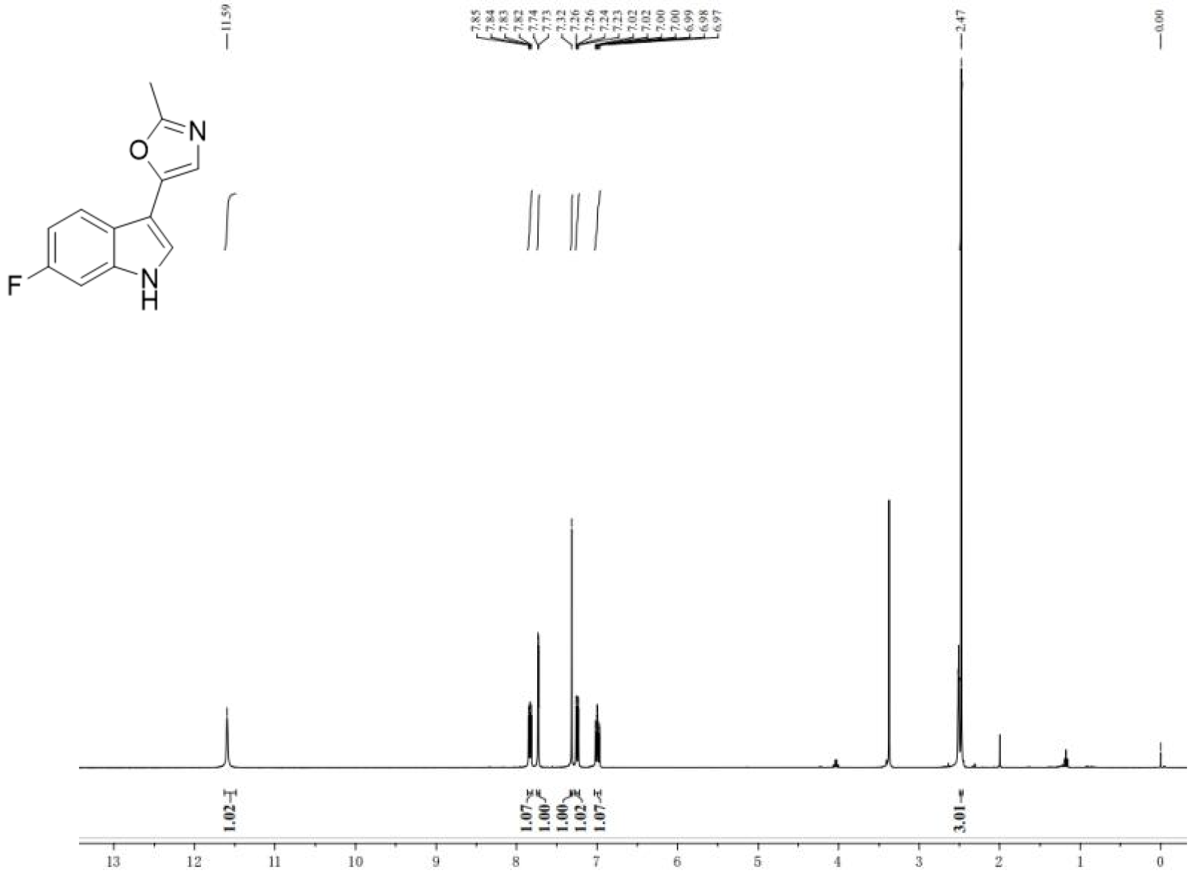

Compound 8f

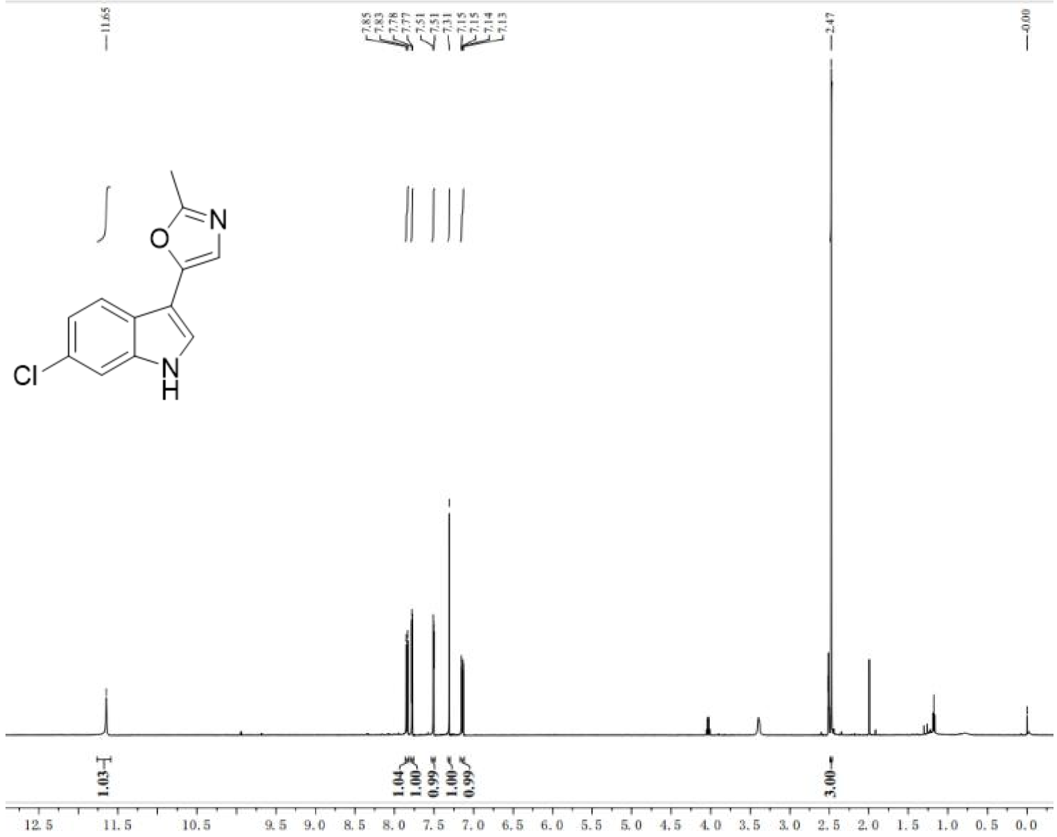

Compound 8g

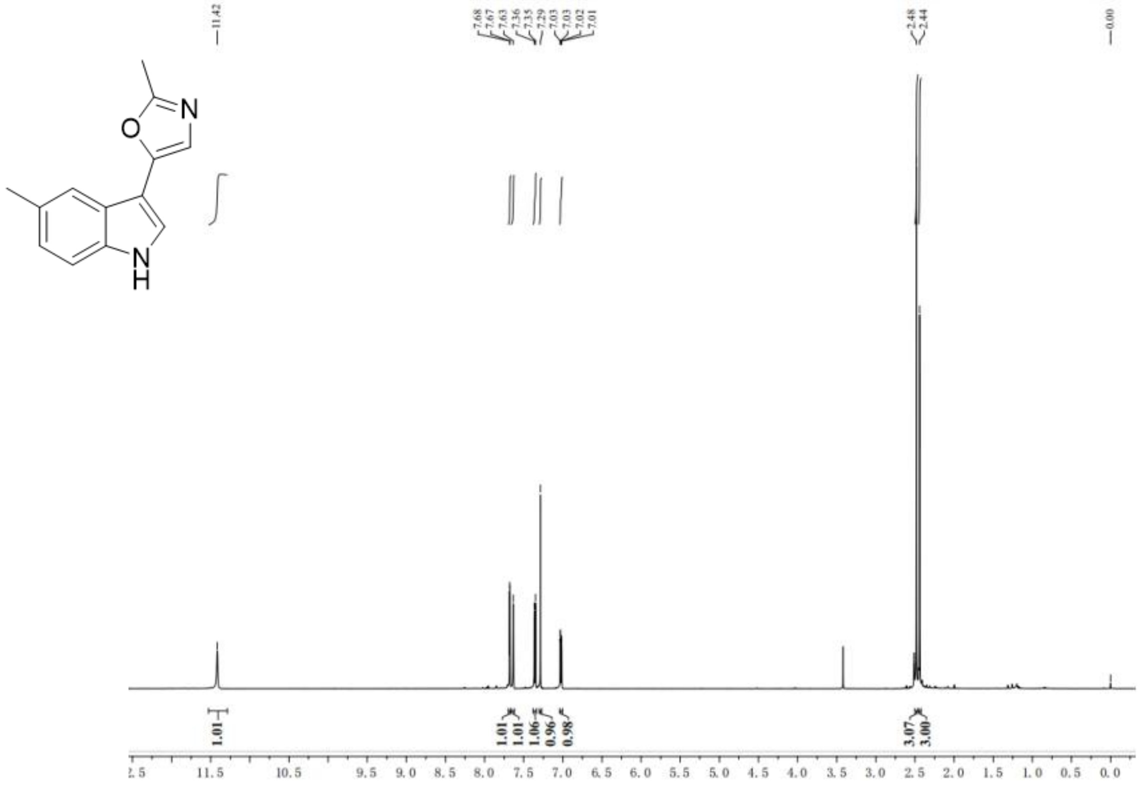

Compound 8h

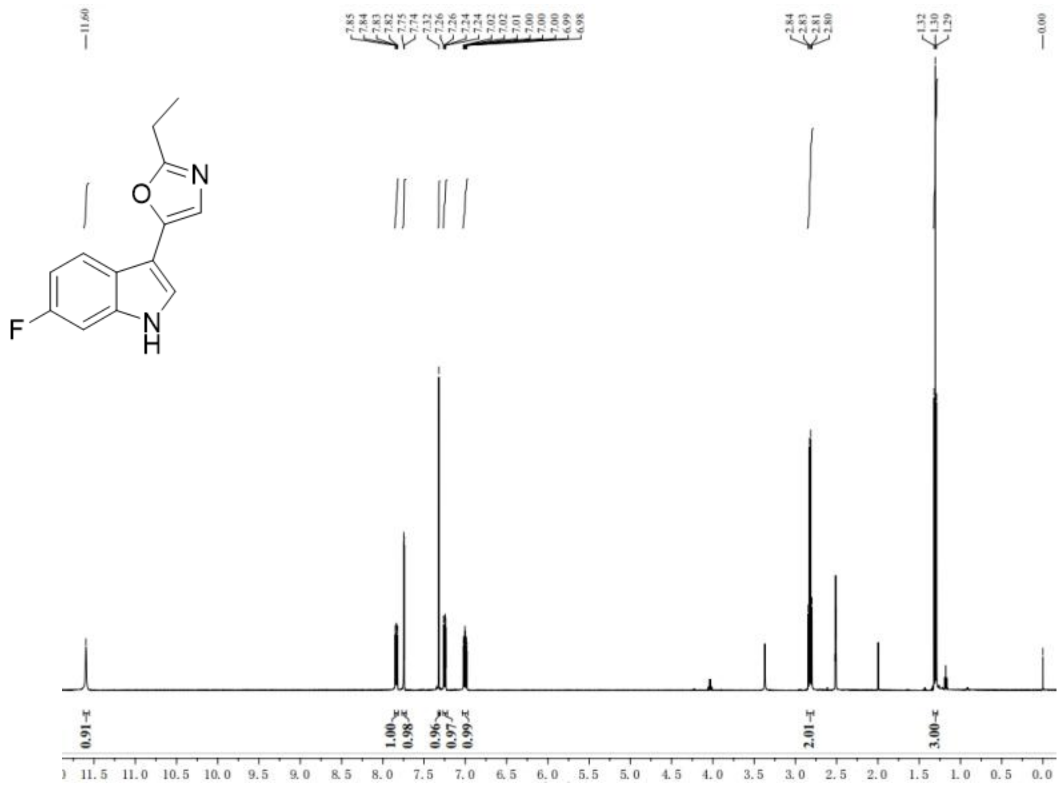

Compound 8i

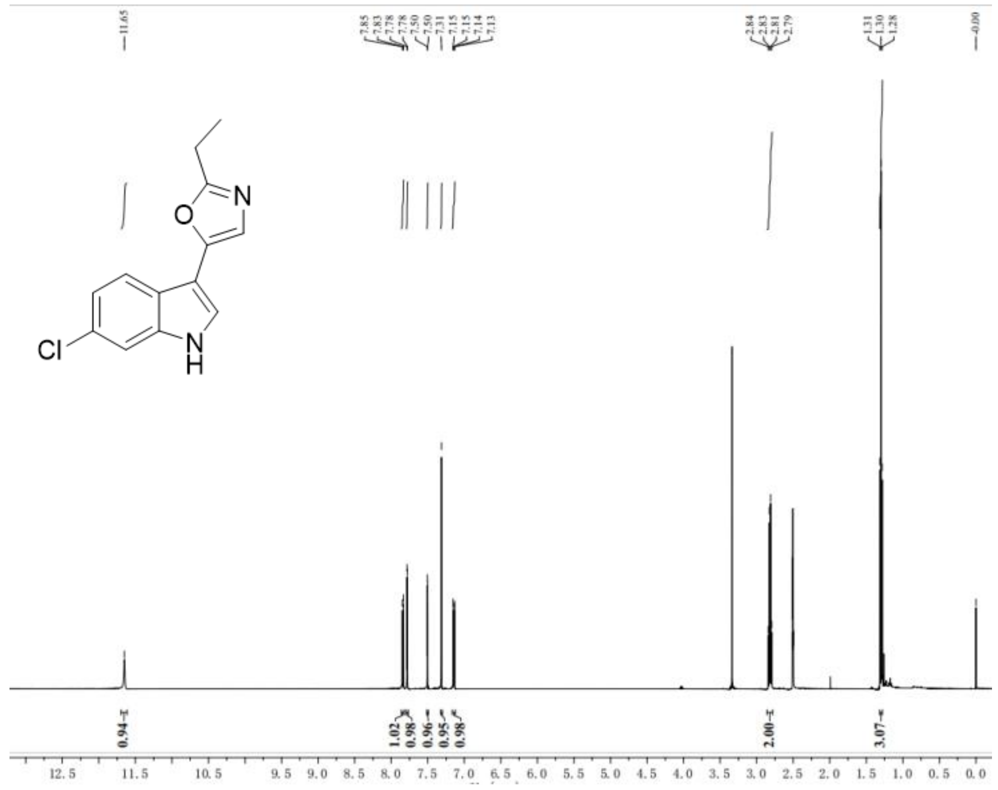

Compound 8j

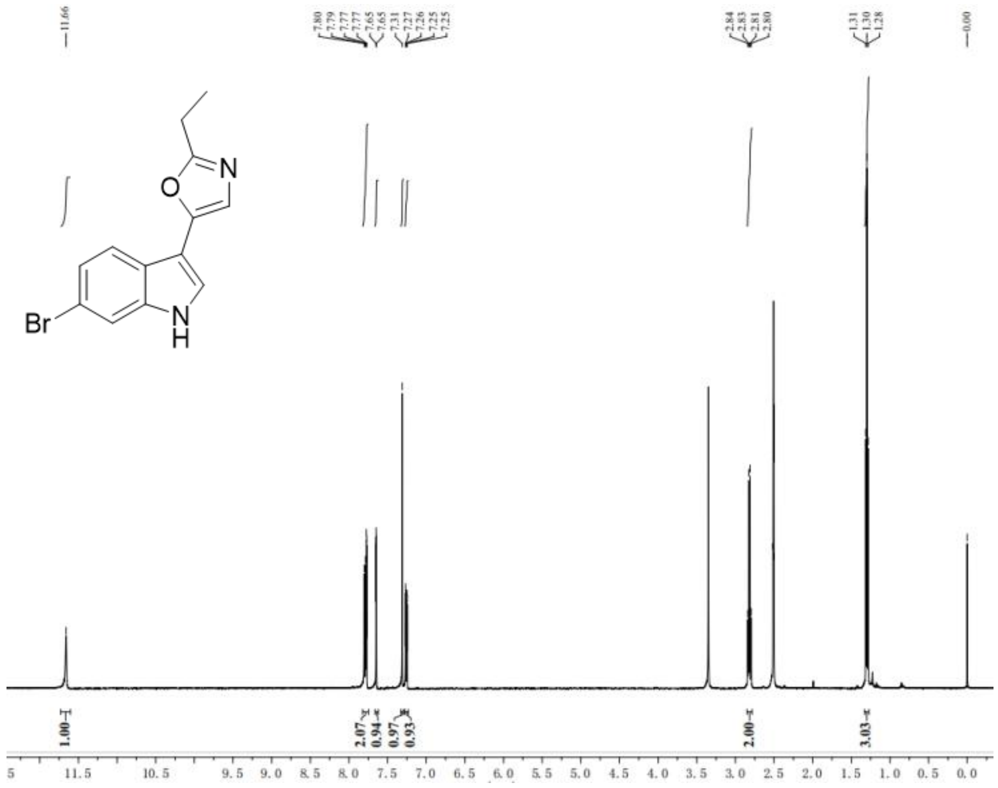

Compound 8k

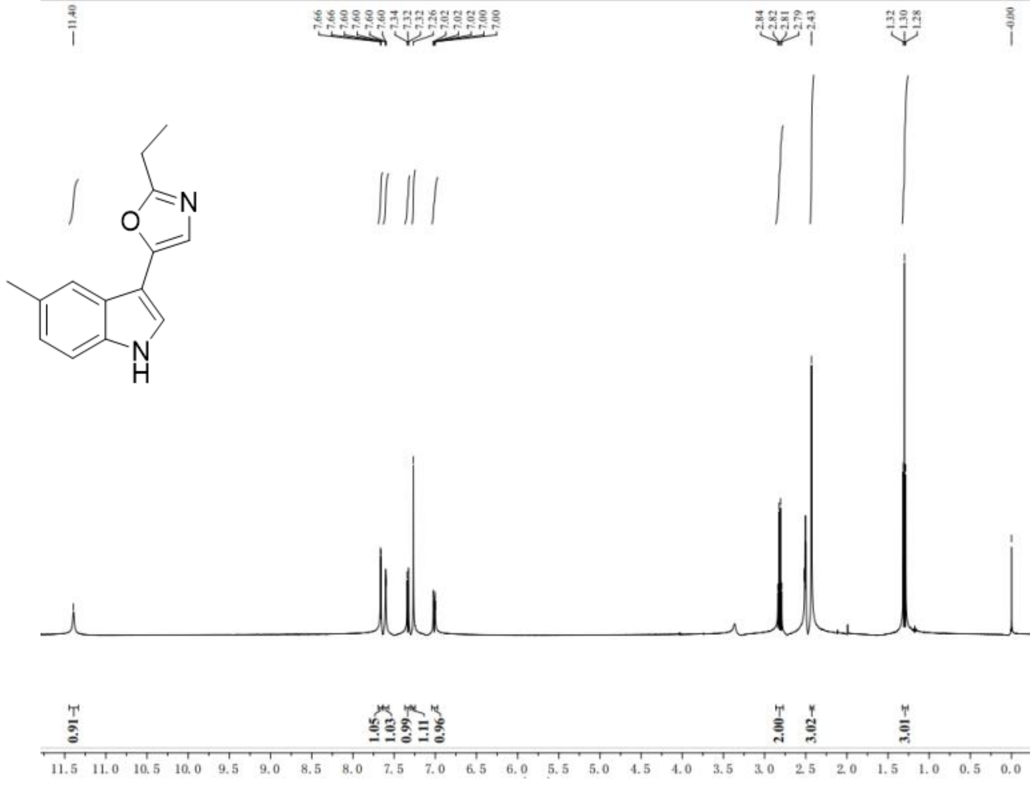

Compound 9a

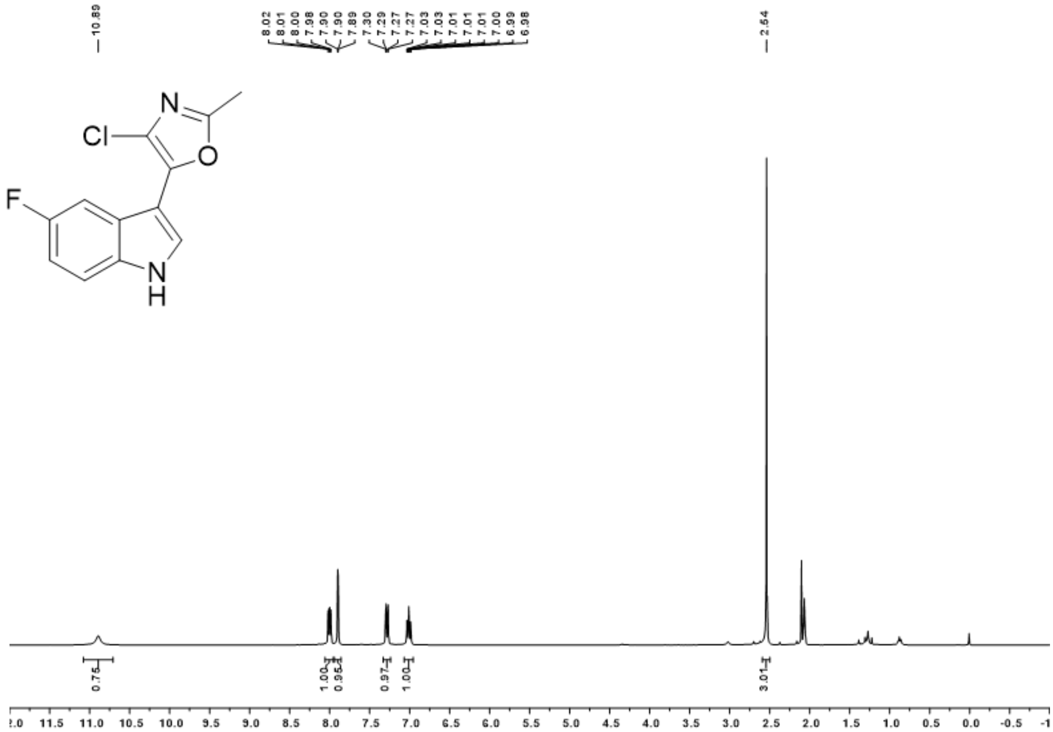

Compound 9b

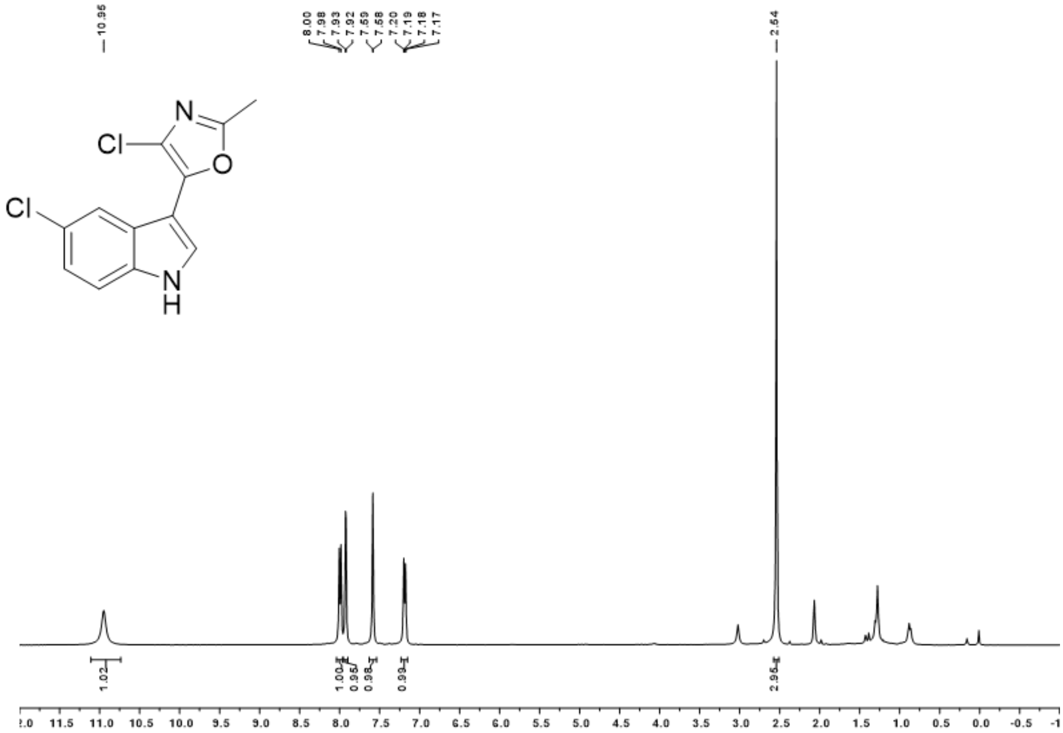

Compound 9c

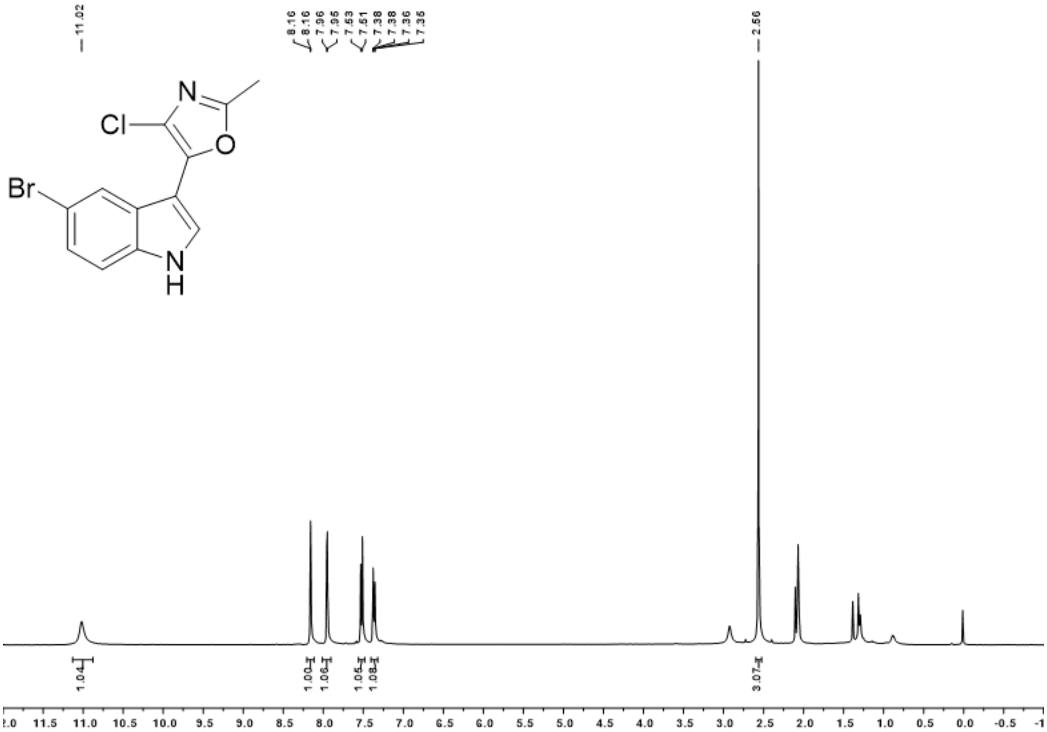

Compound 9d

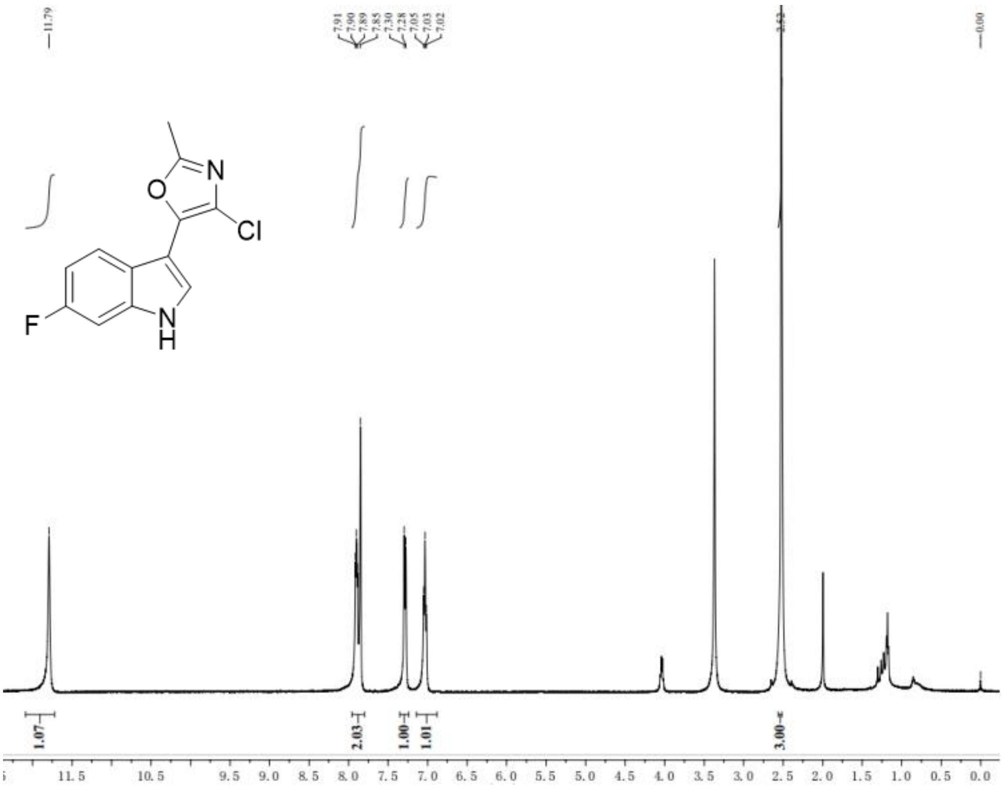

Compound 9e

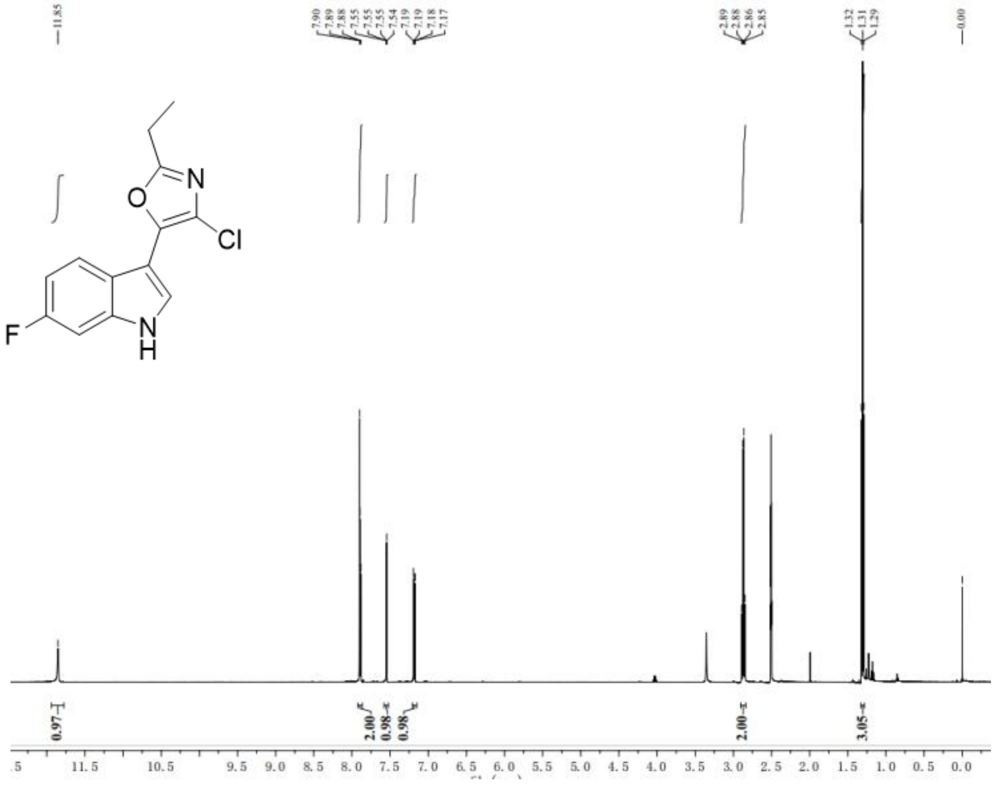

Compound 9f

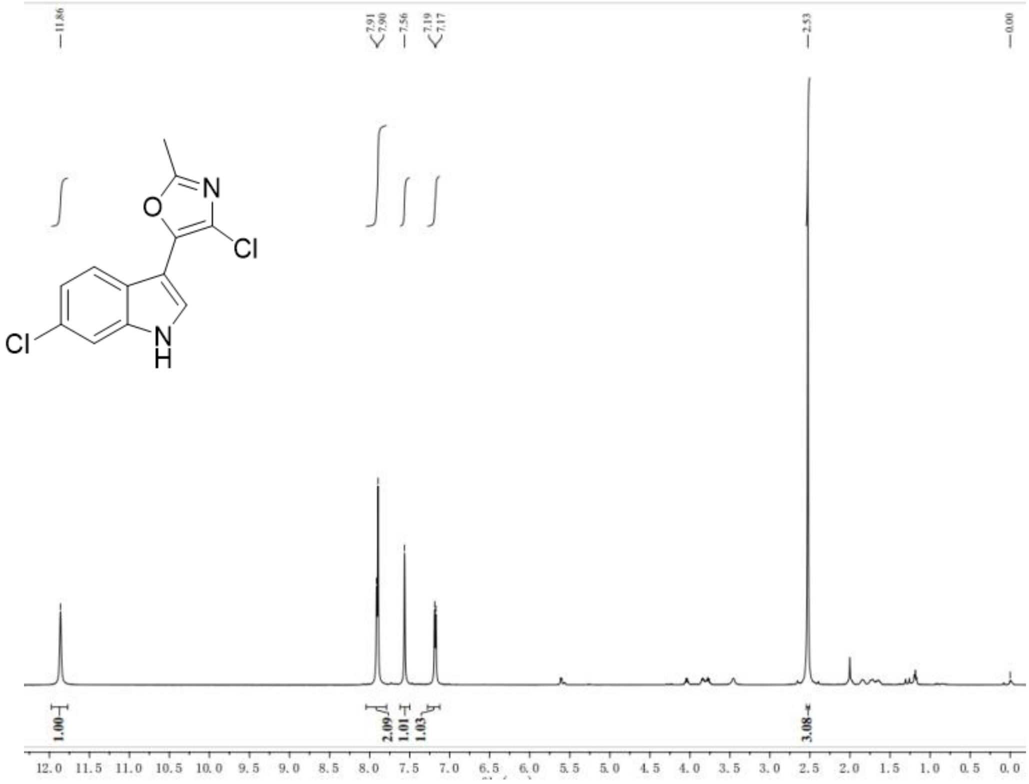

Compound 9g

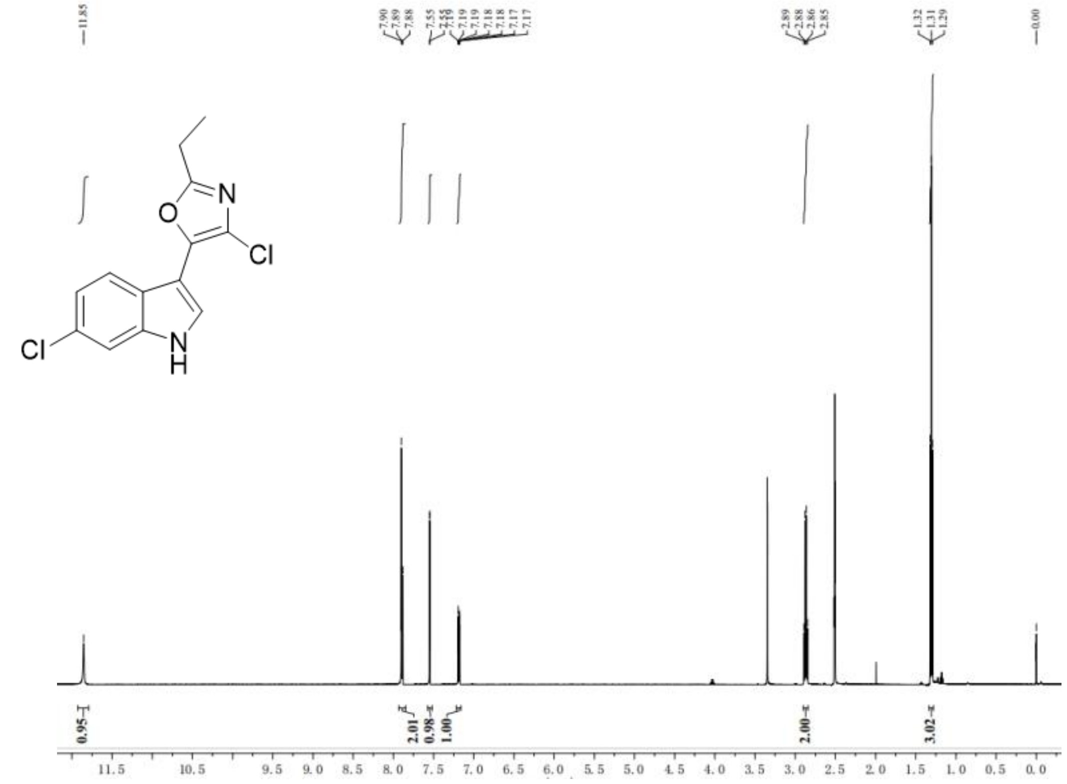

Compound 9h

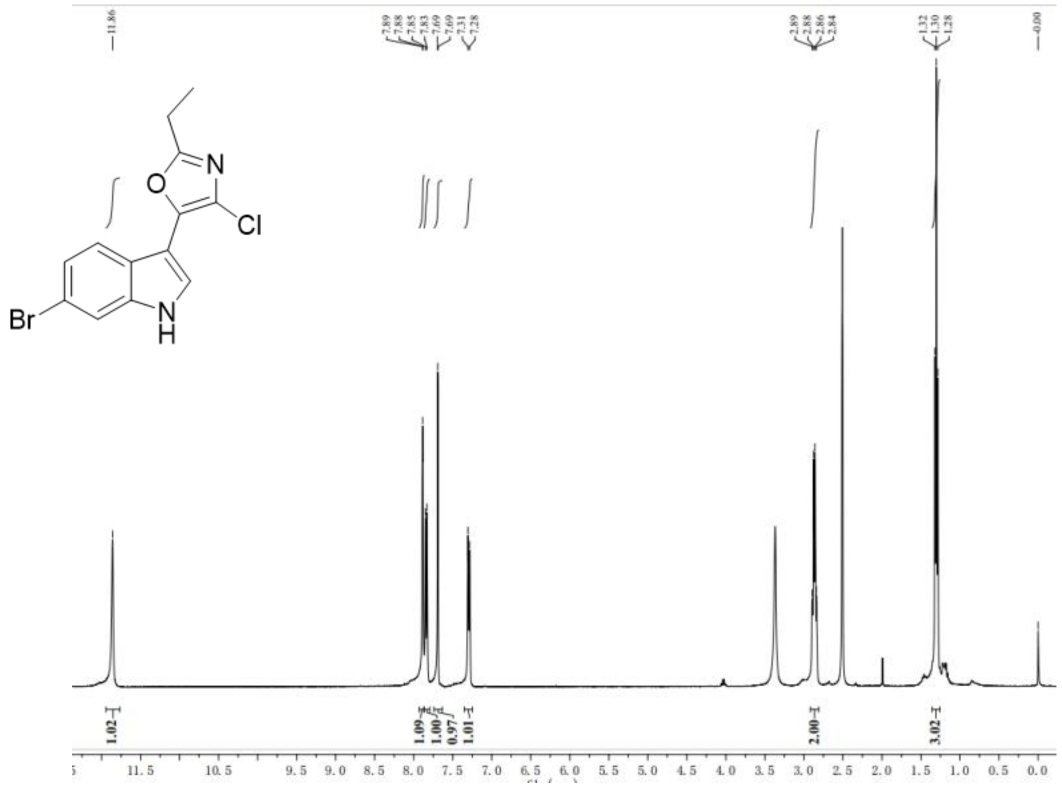

Compound 9i

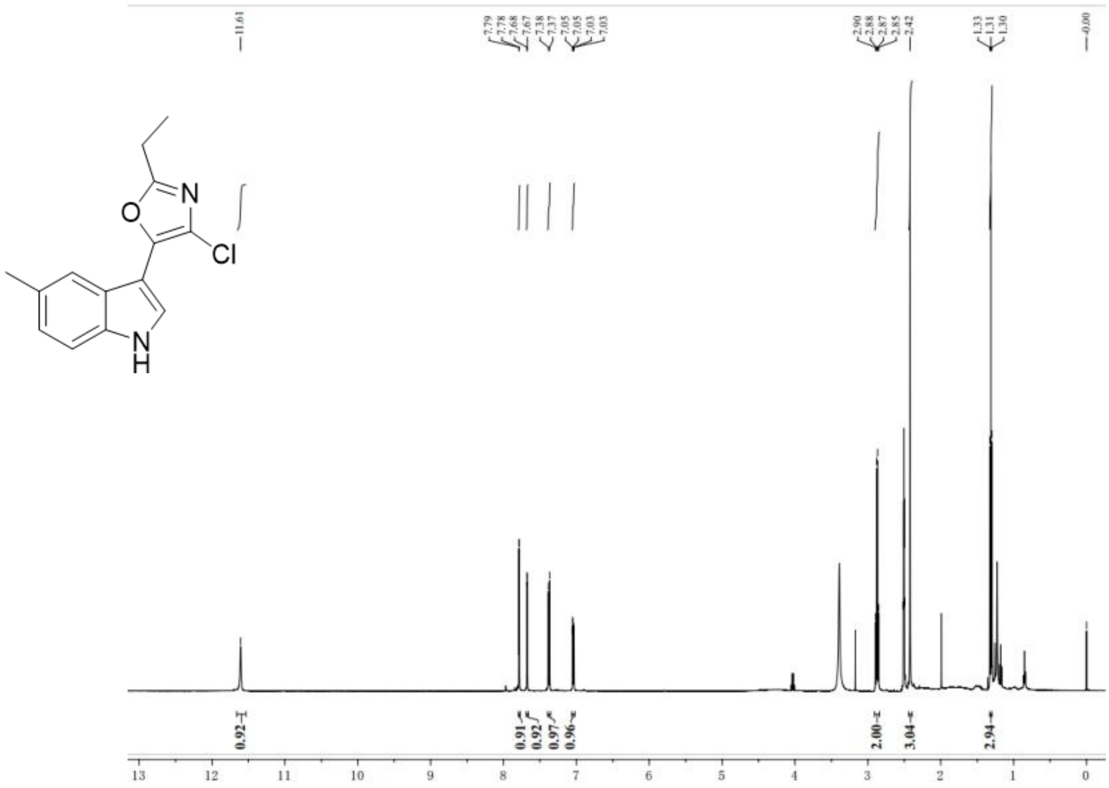

Compound 10a

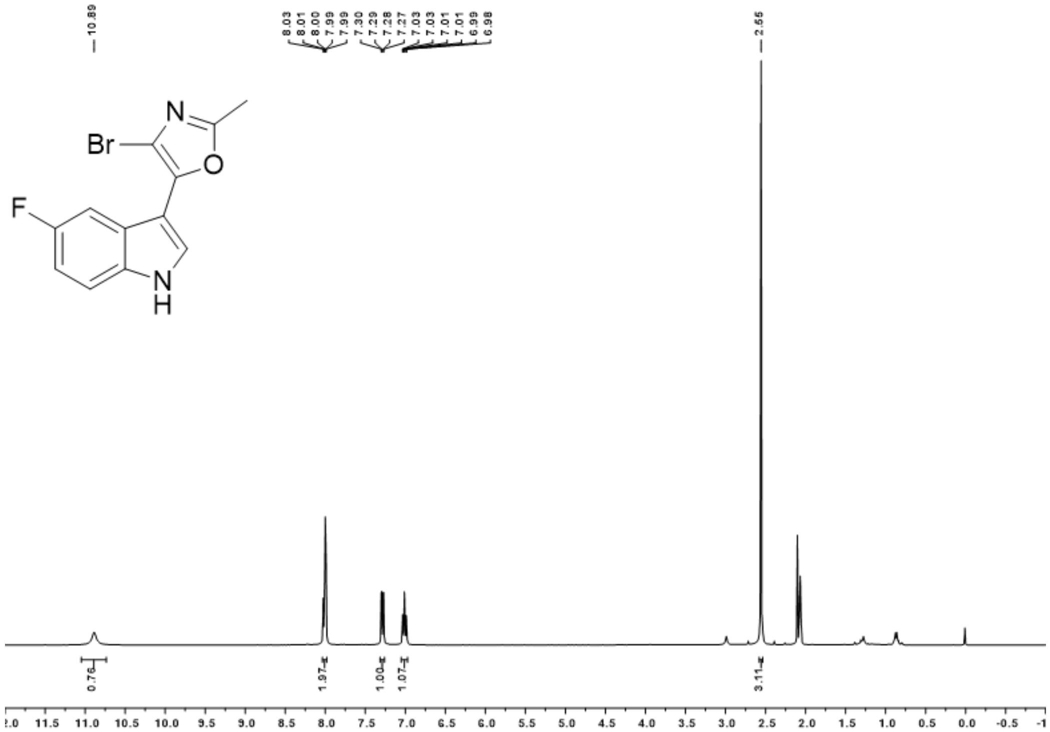

Compound 10b

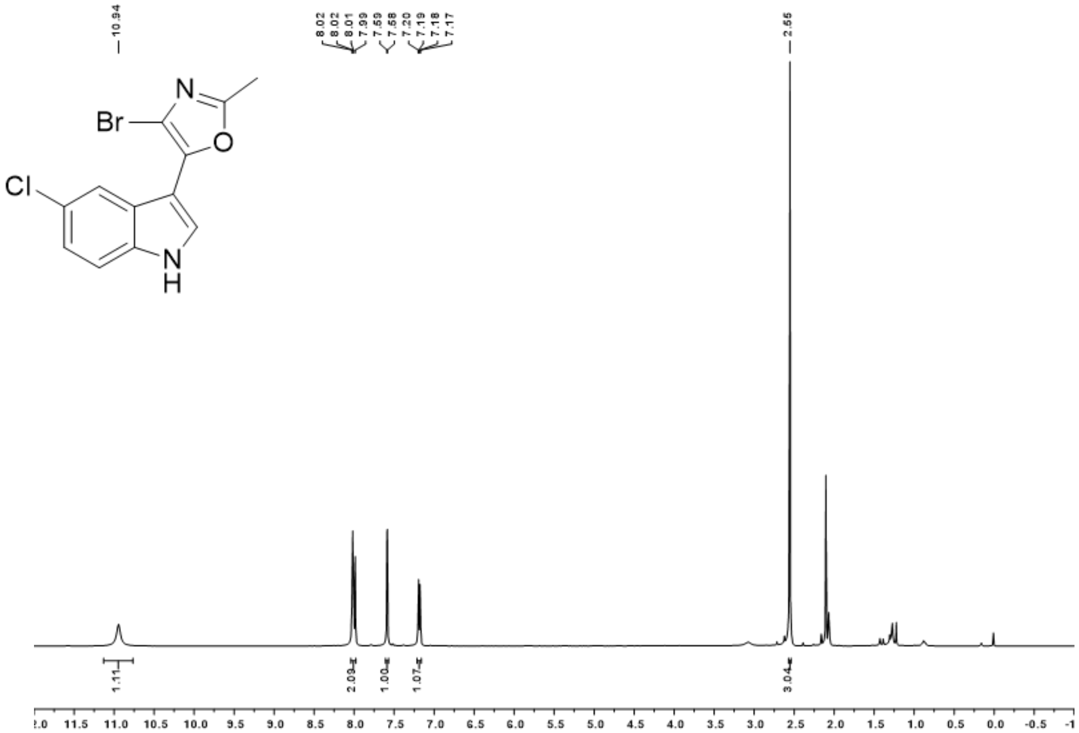

Compound 10c

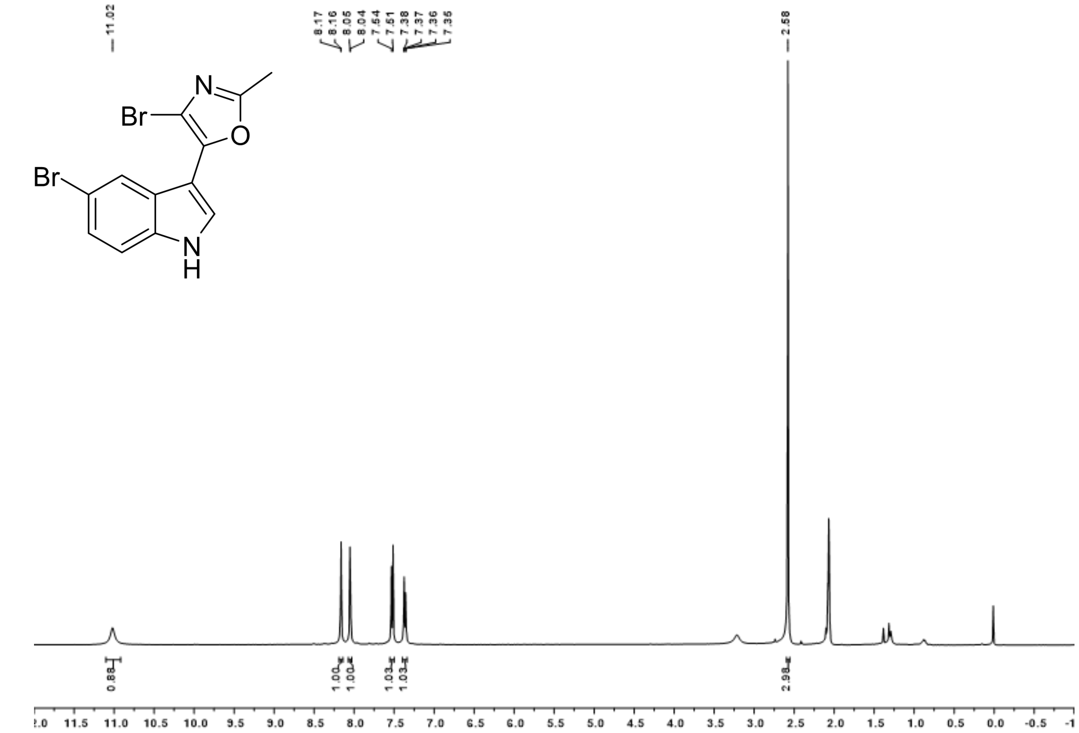

Compound 10d

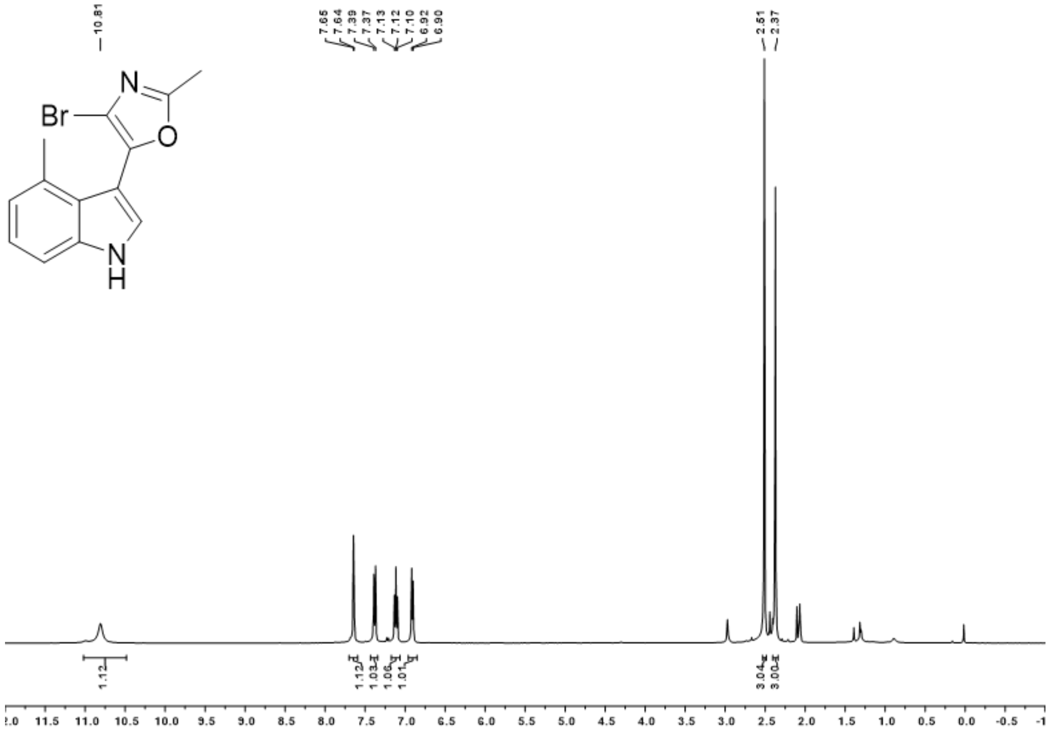

Compound 10e

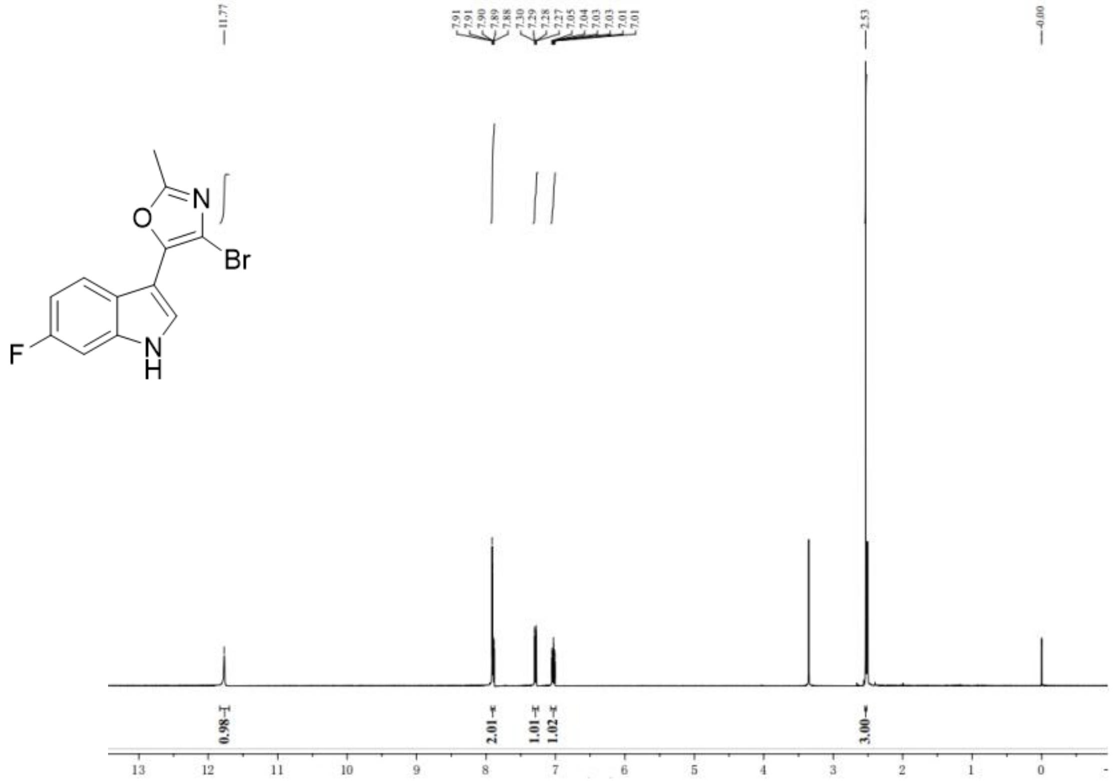

Compound 10f

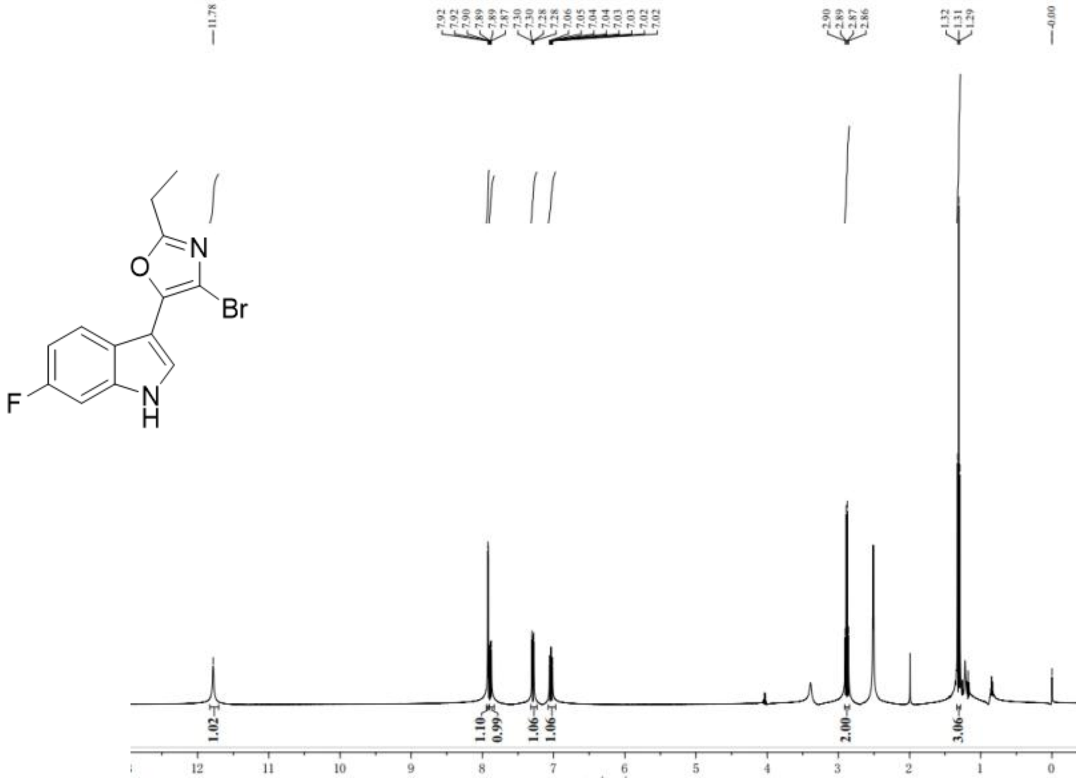

Compound 10g

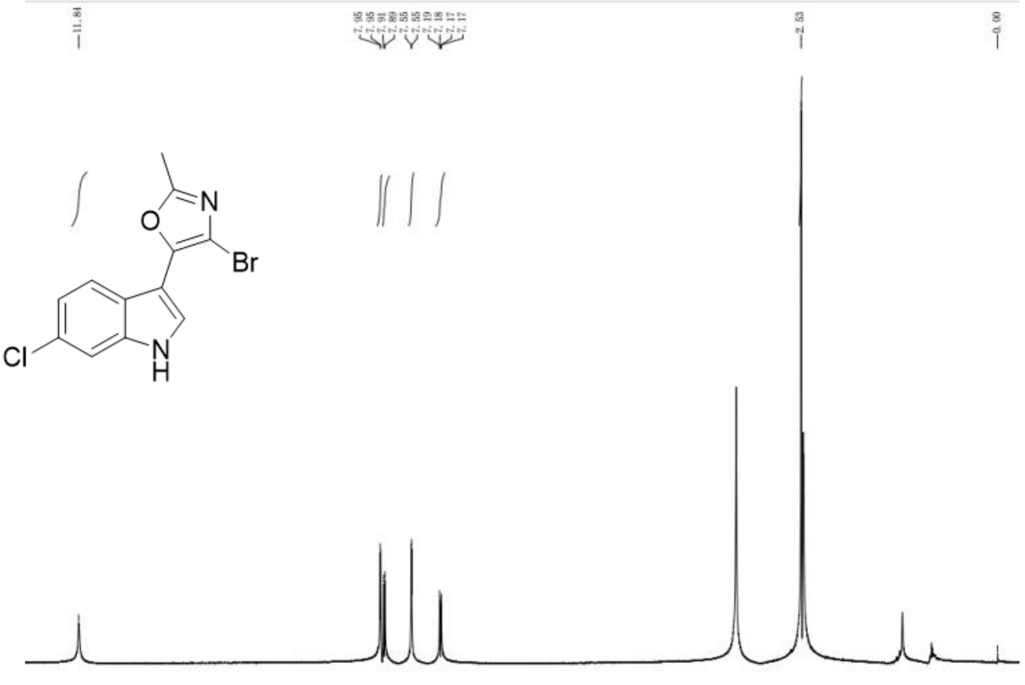

Compound 10h

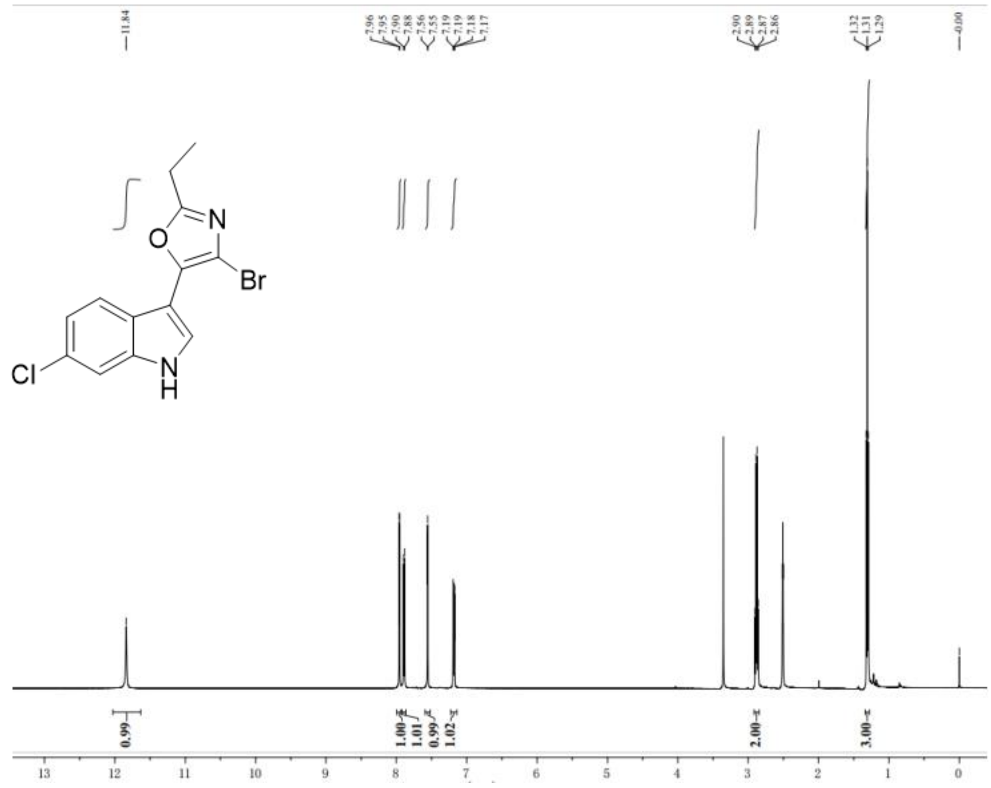

Compound 10i

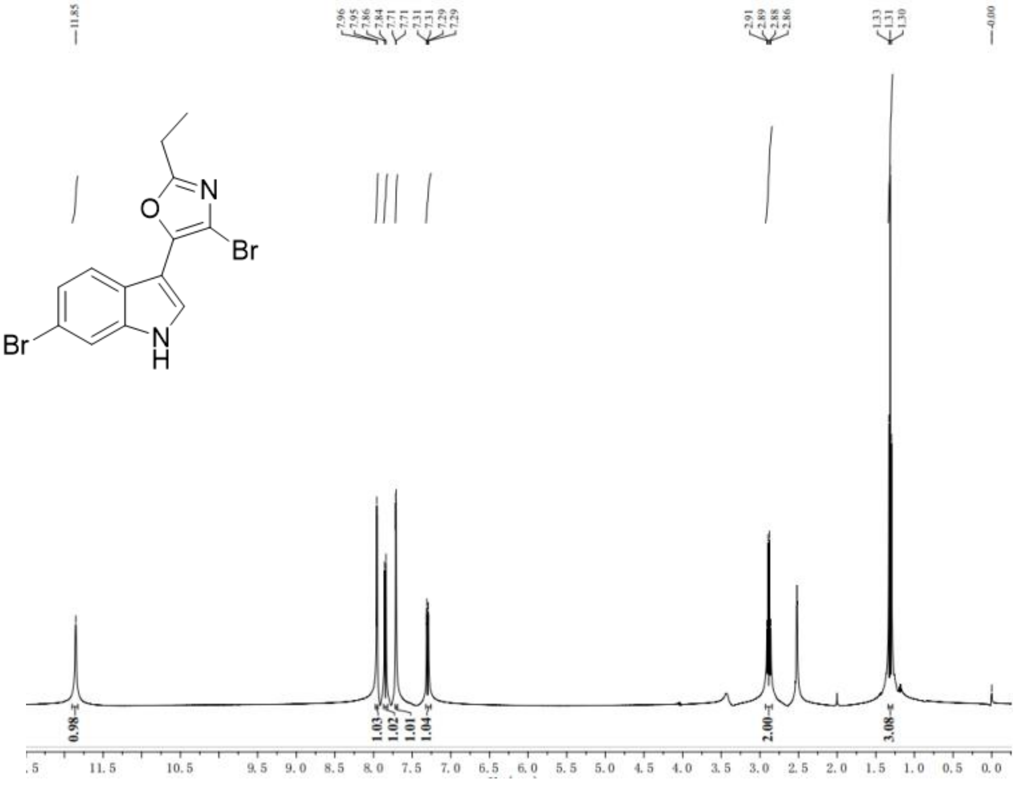

3. ^{13}C NMR

Compound 3a

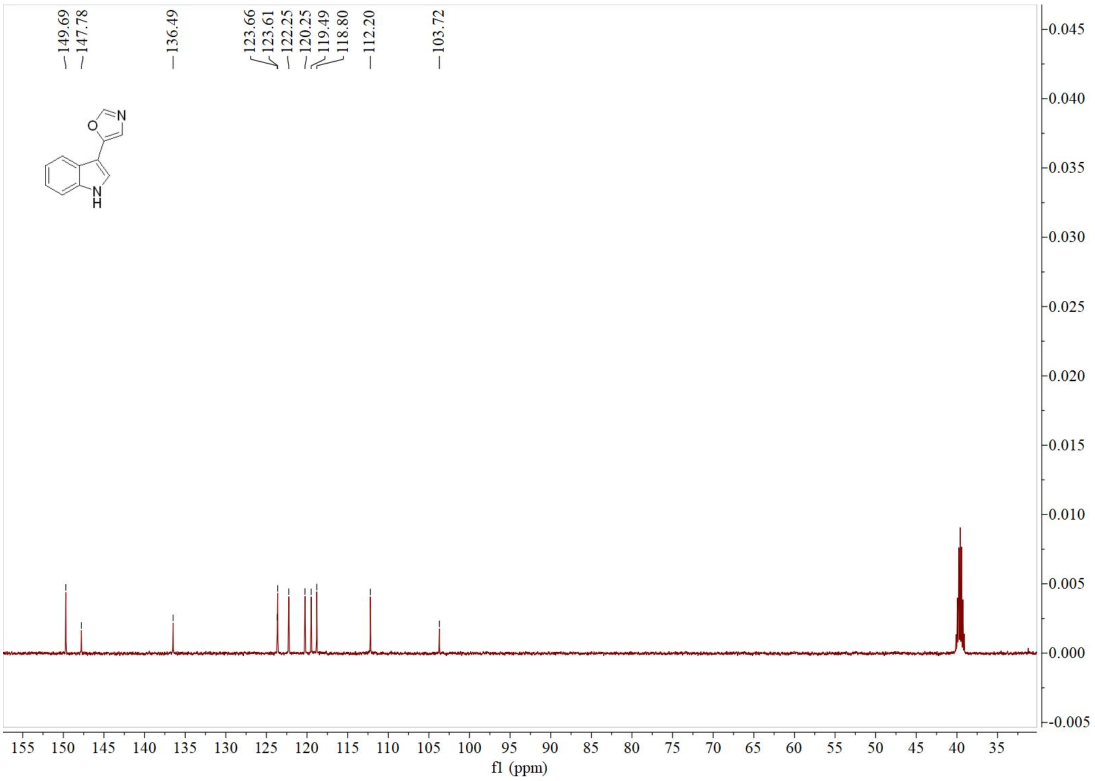

Compound 3b

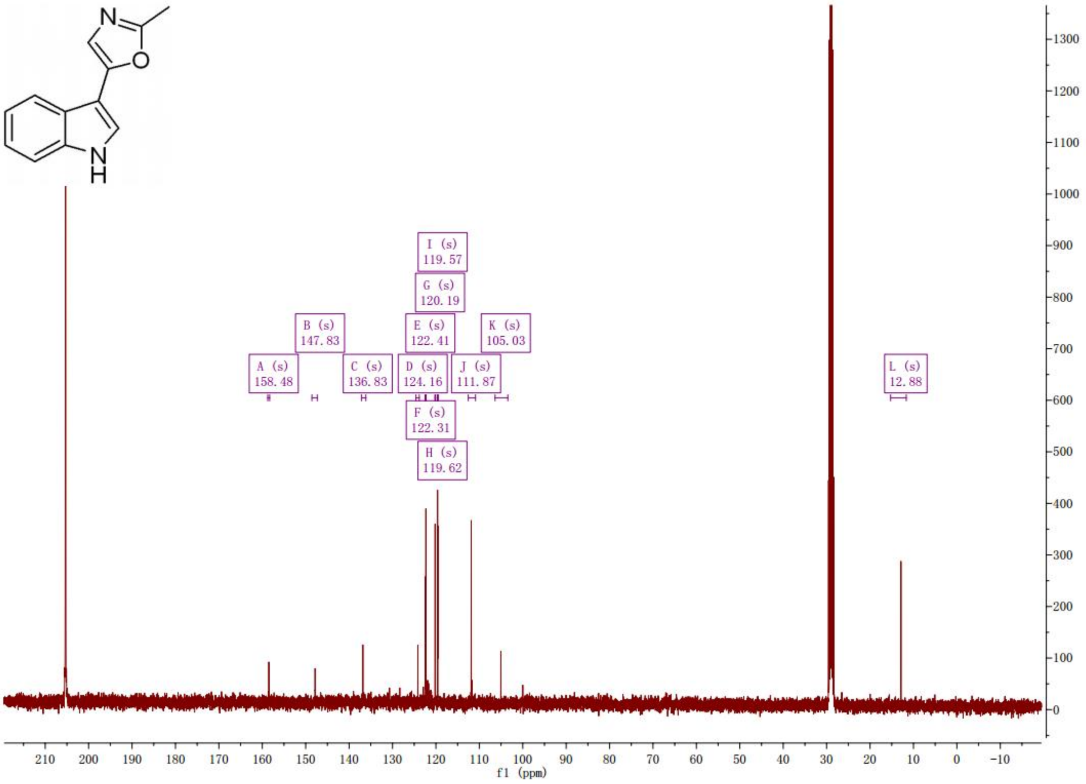

Compound 3c

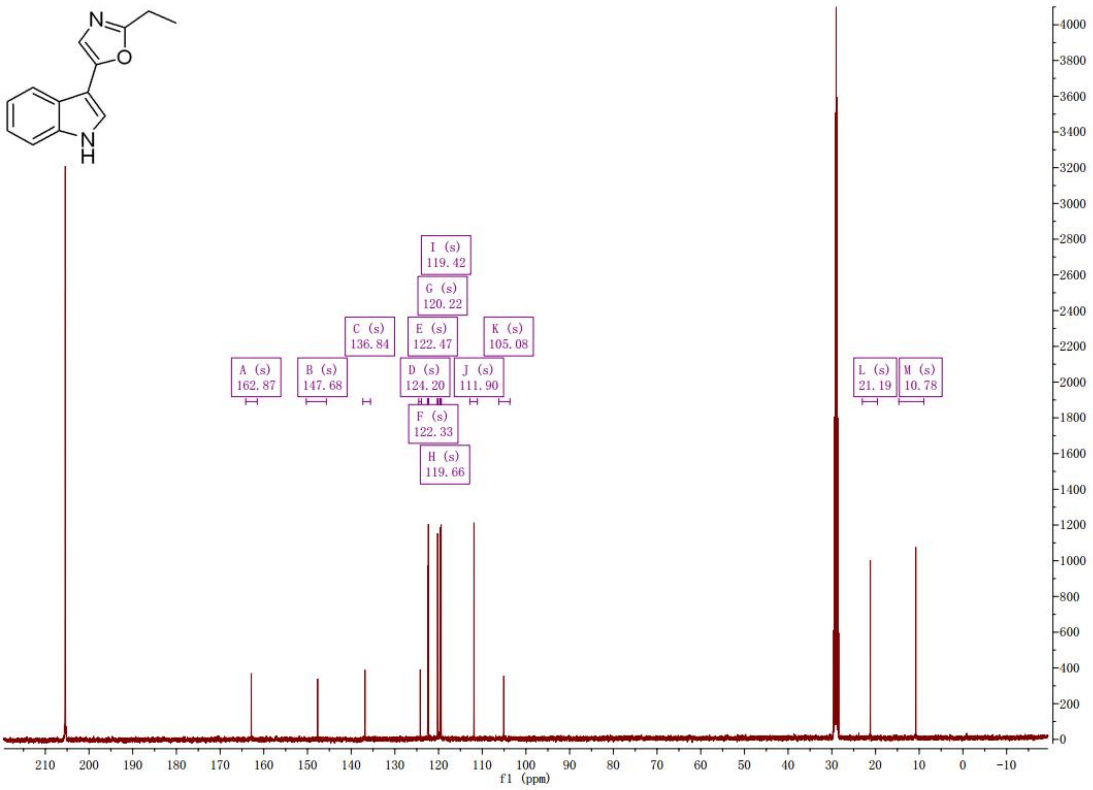

Compound 3d

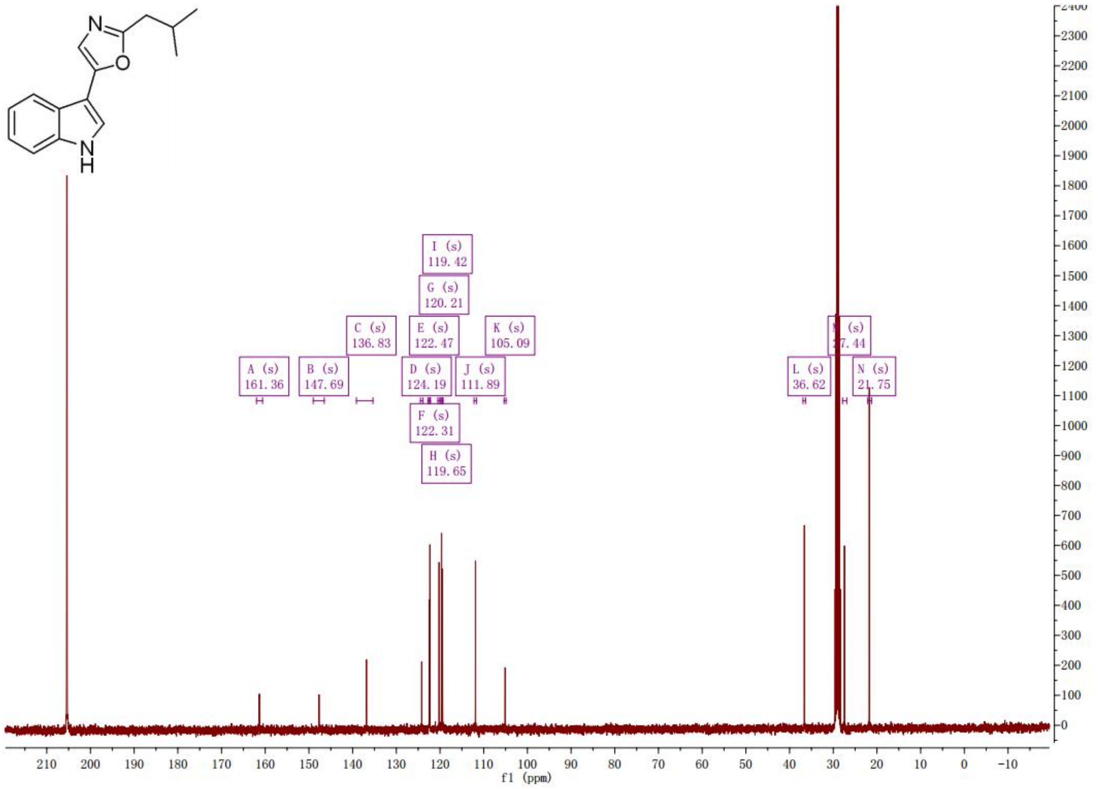

Compound 3e

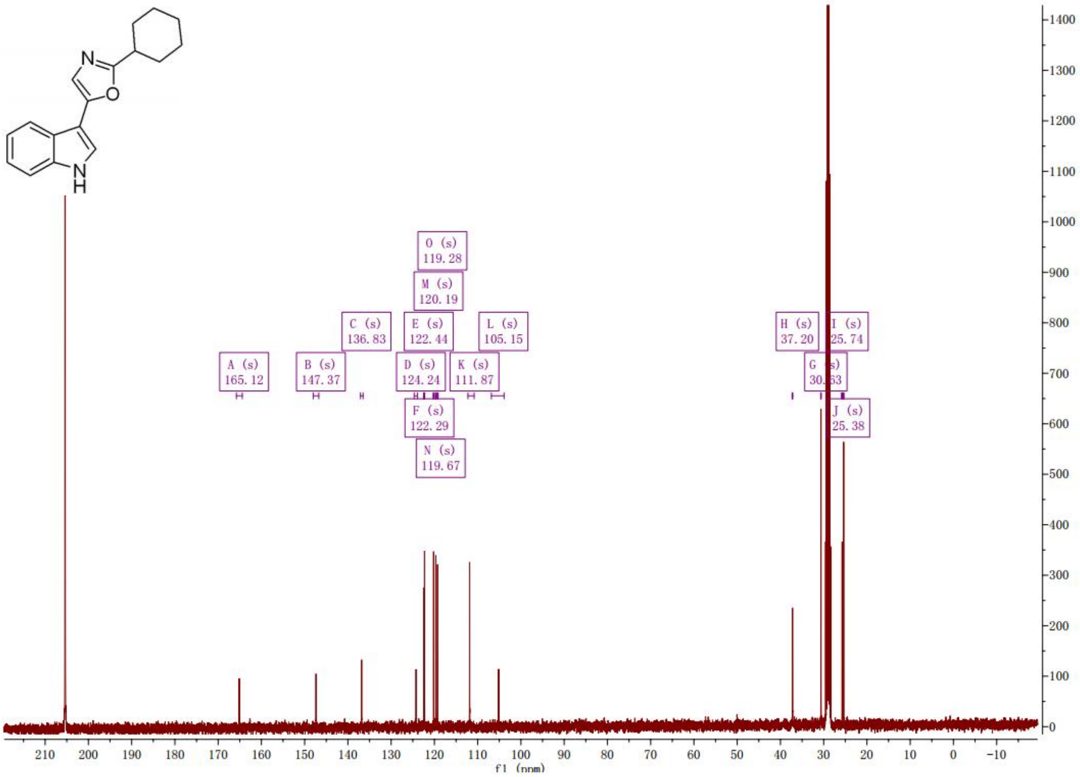

Compound 3f

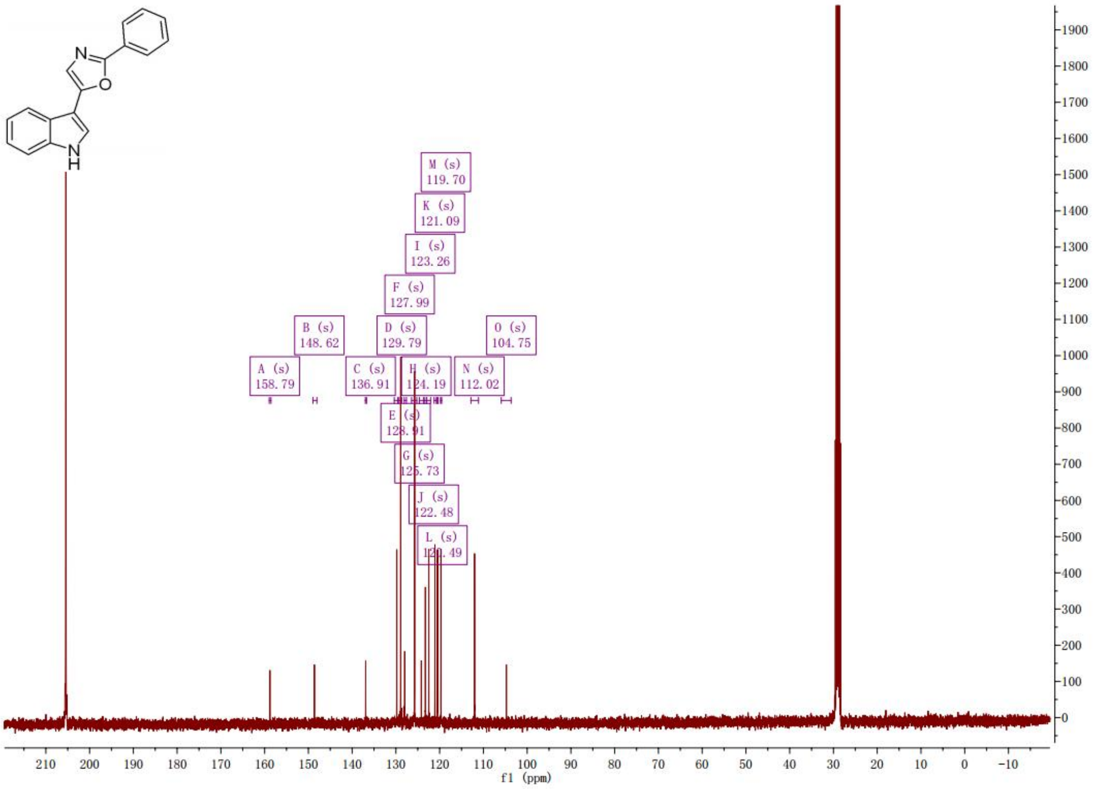

Compound 3g

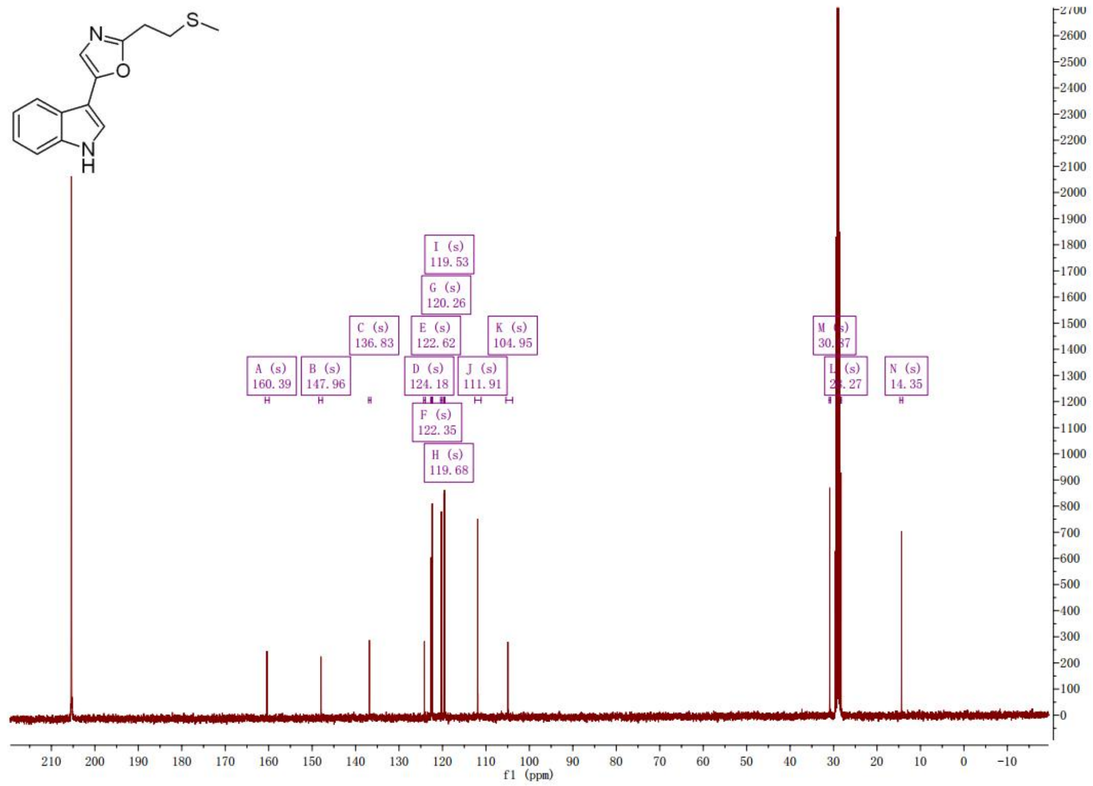

Compound 4a

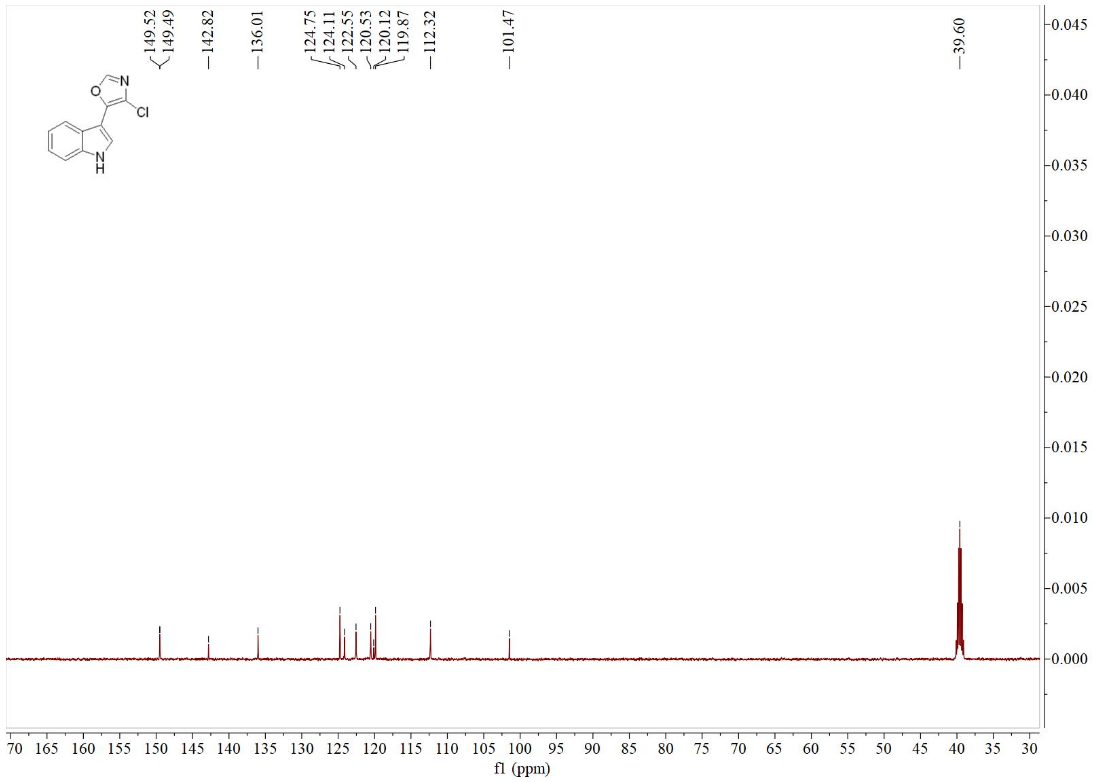

Compound 4b

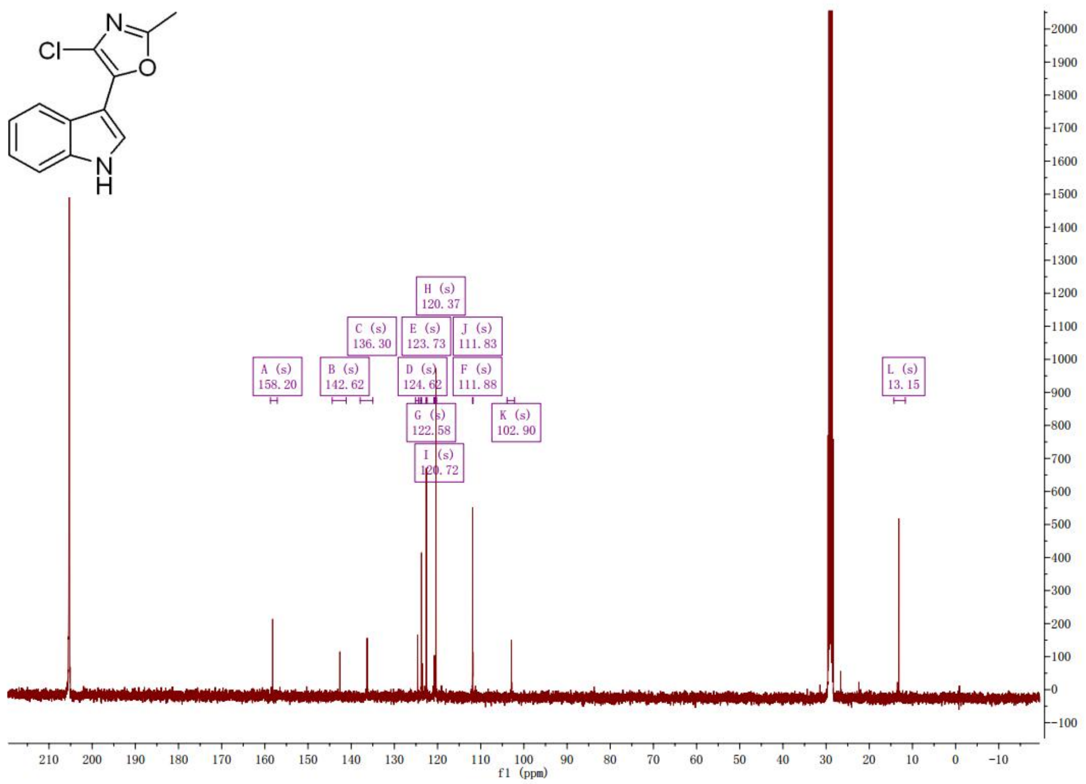

Compound 4c

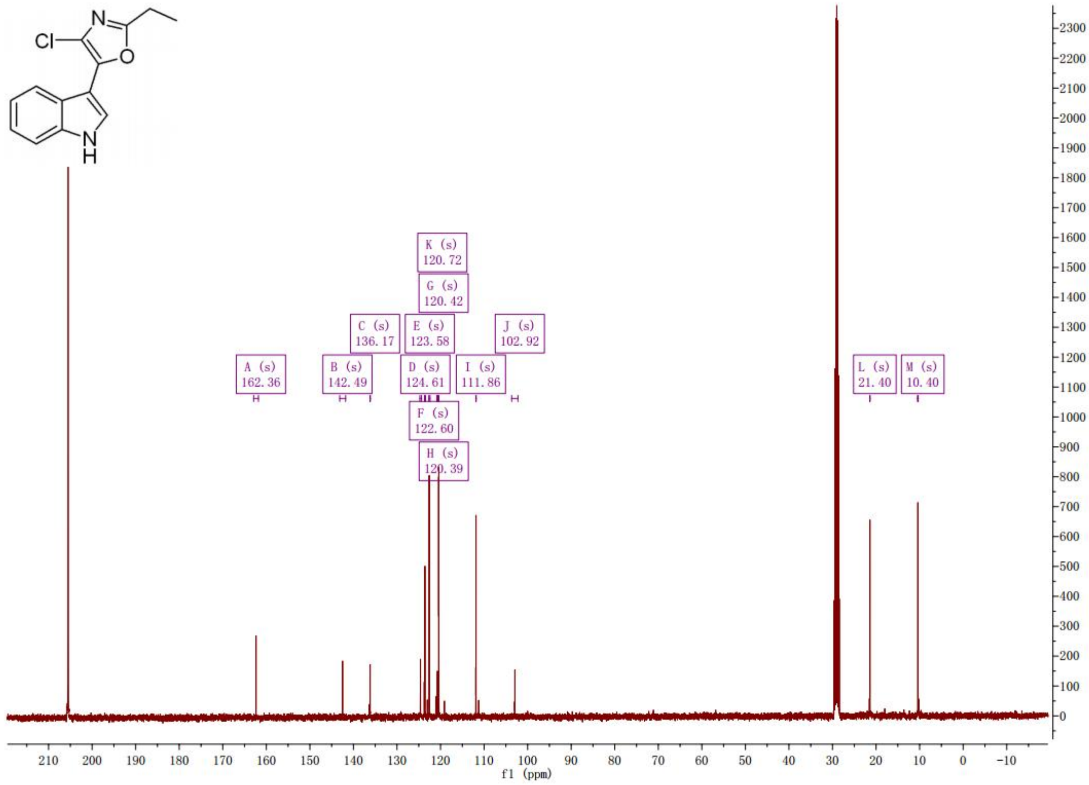

Compound 4d

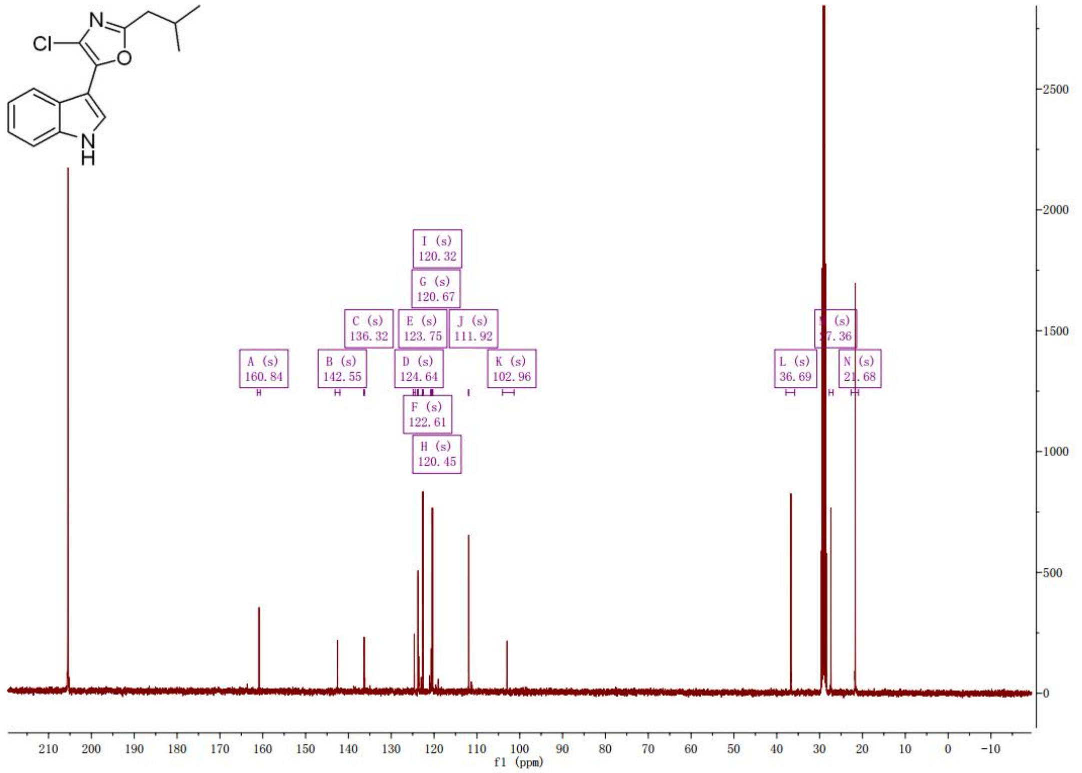

Compound 4e

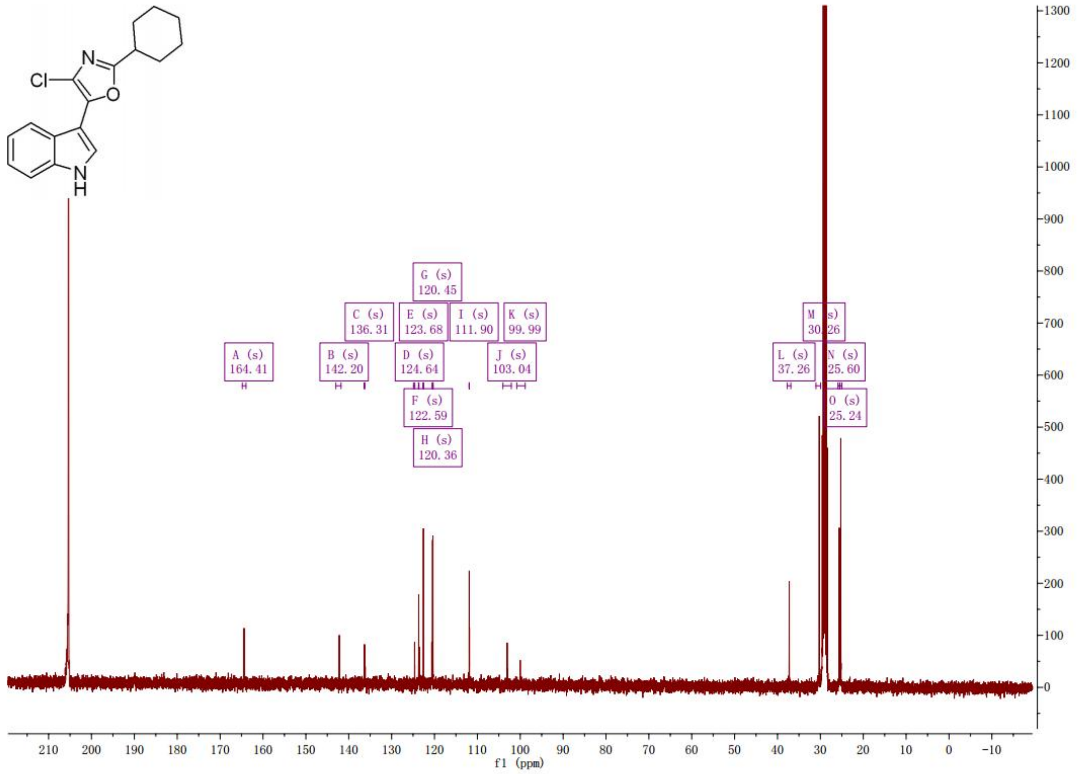

Compound 4f

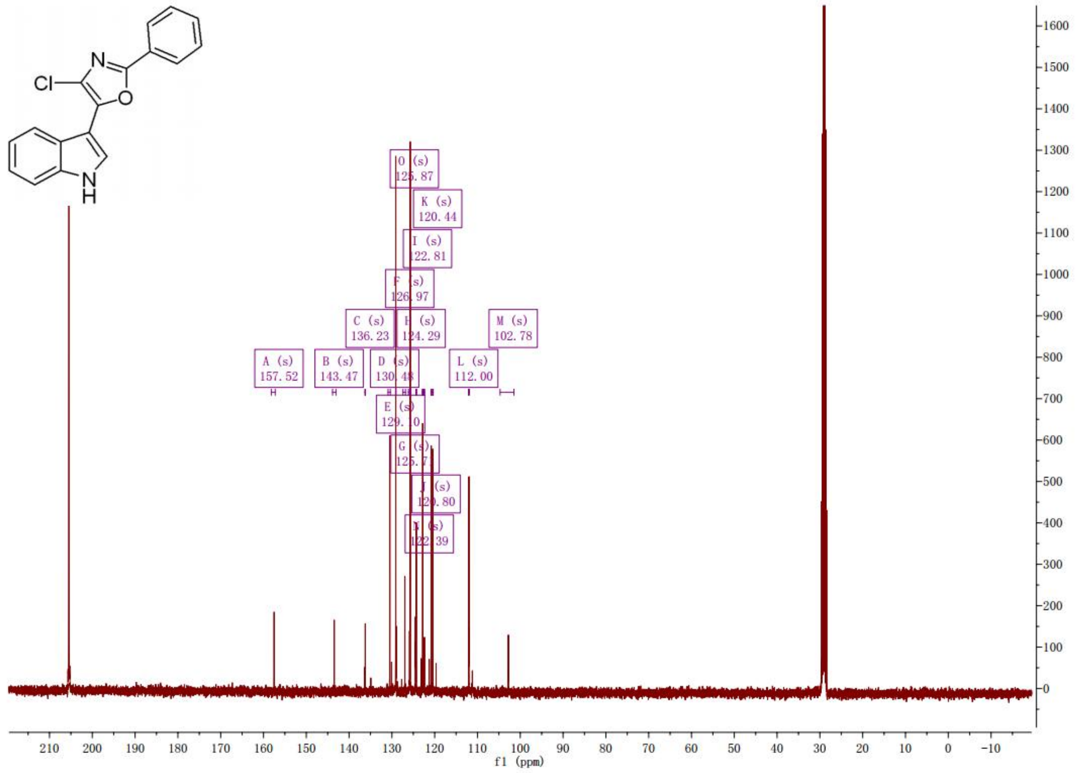

Compound 5a

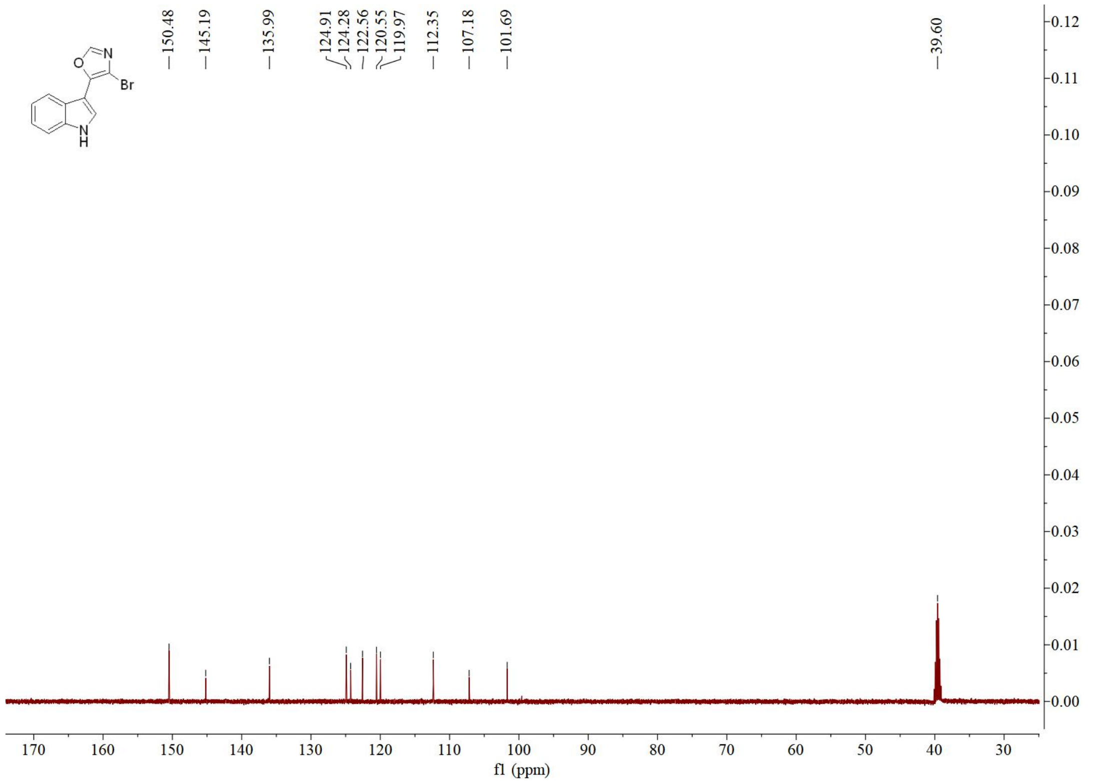

Compound 5b

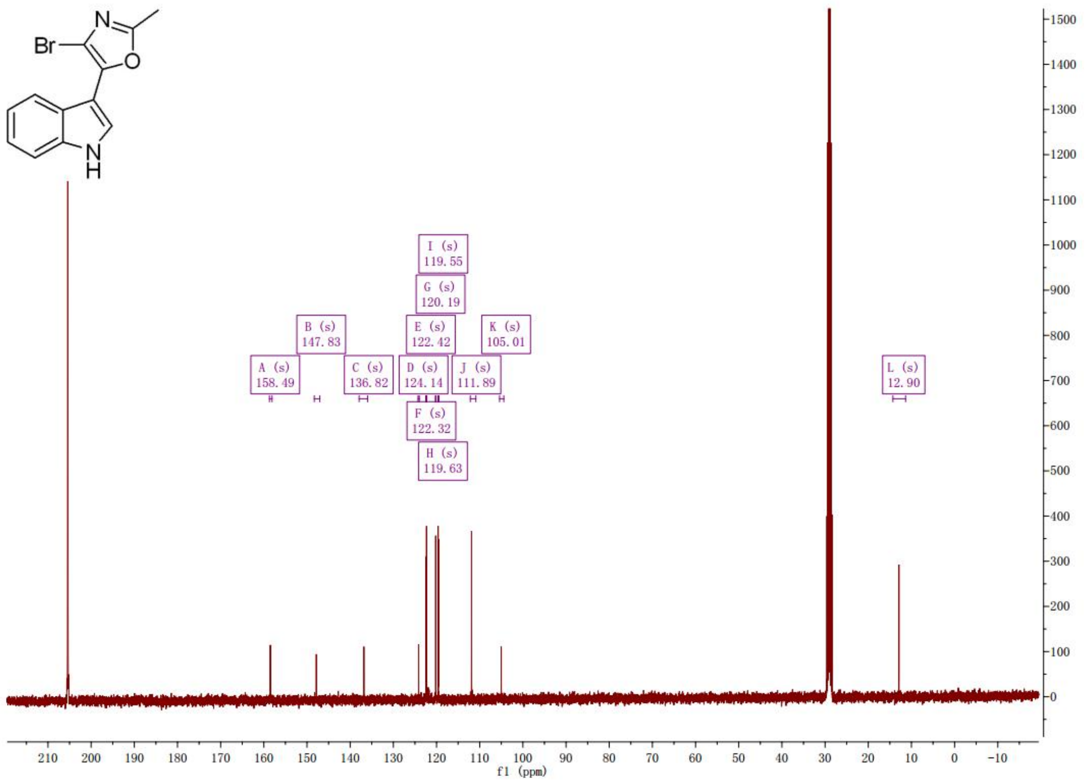

Compound 5c

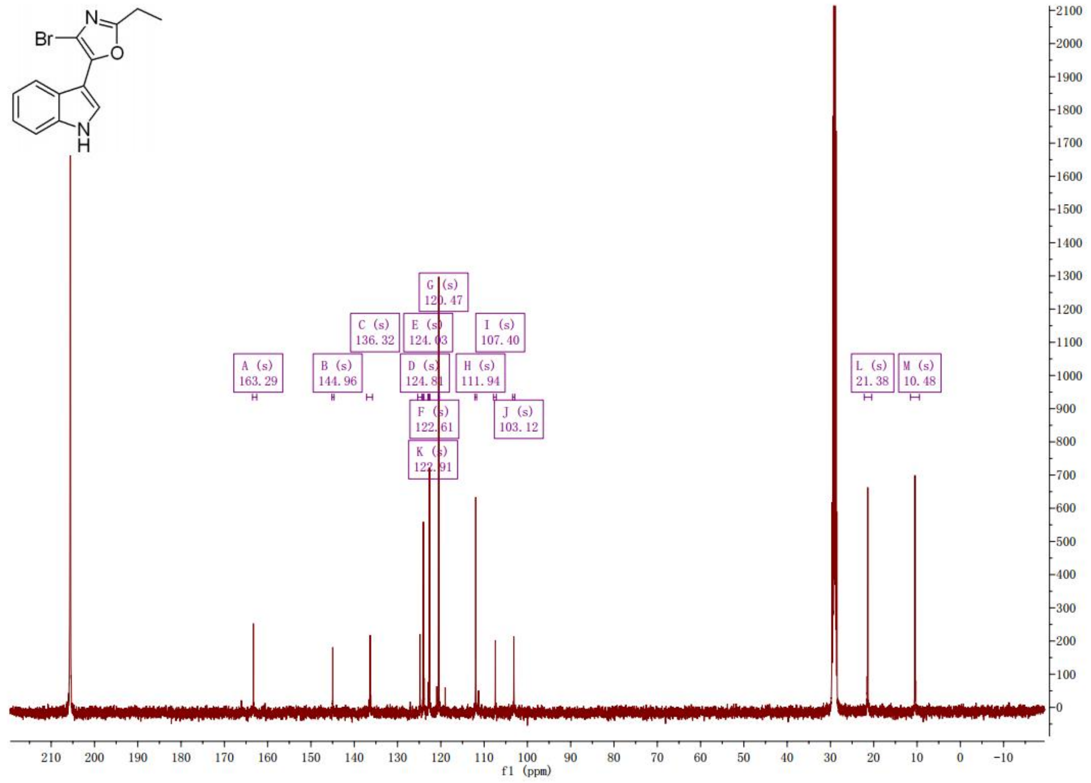

Compound 5d

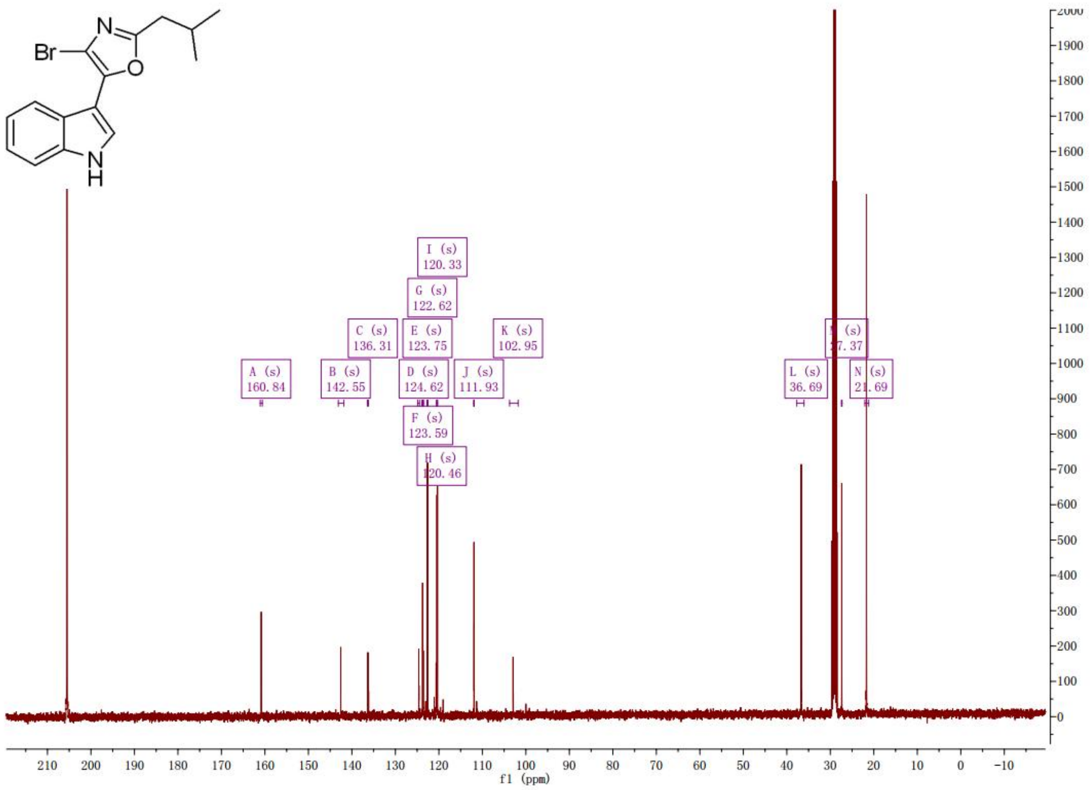

Compound 5e

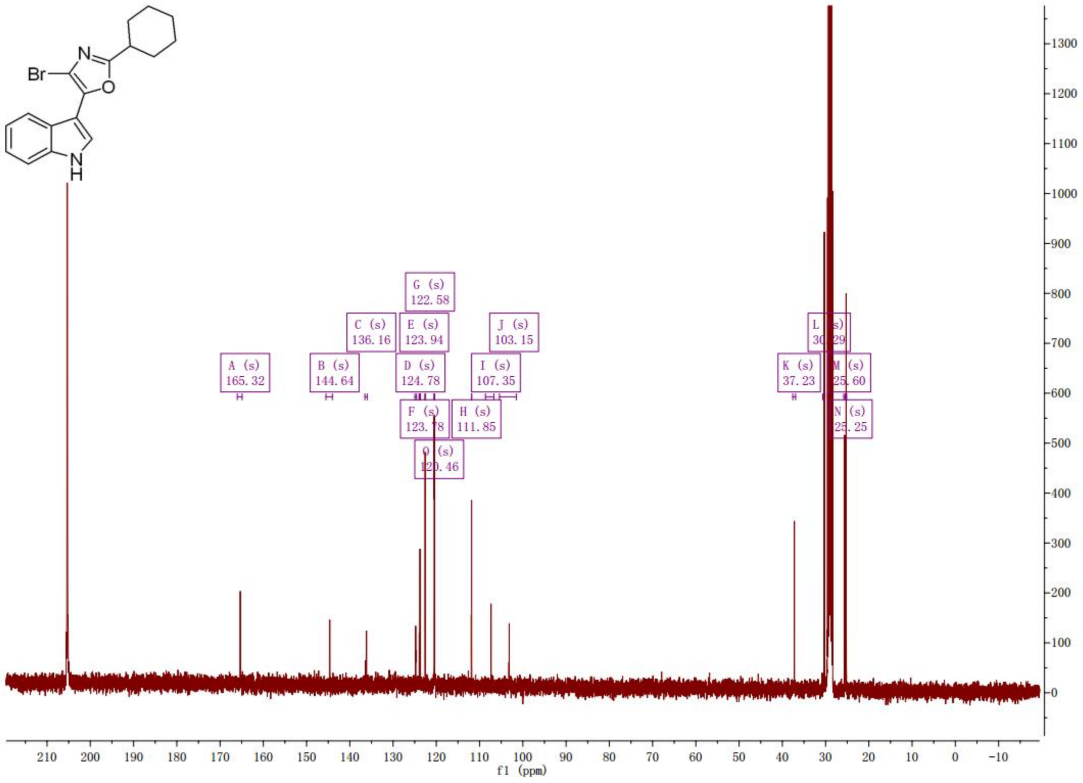

Compound 5f

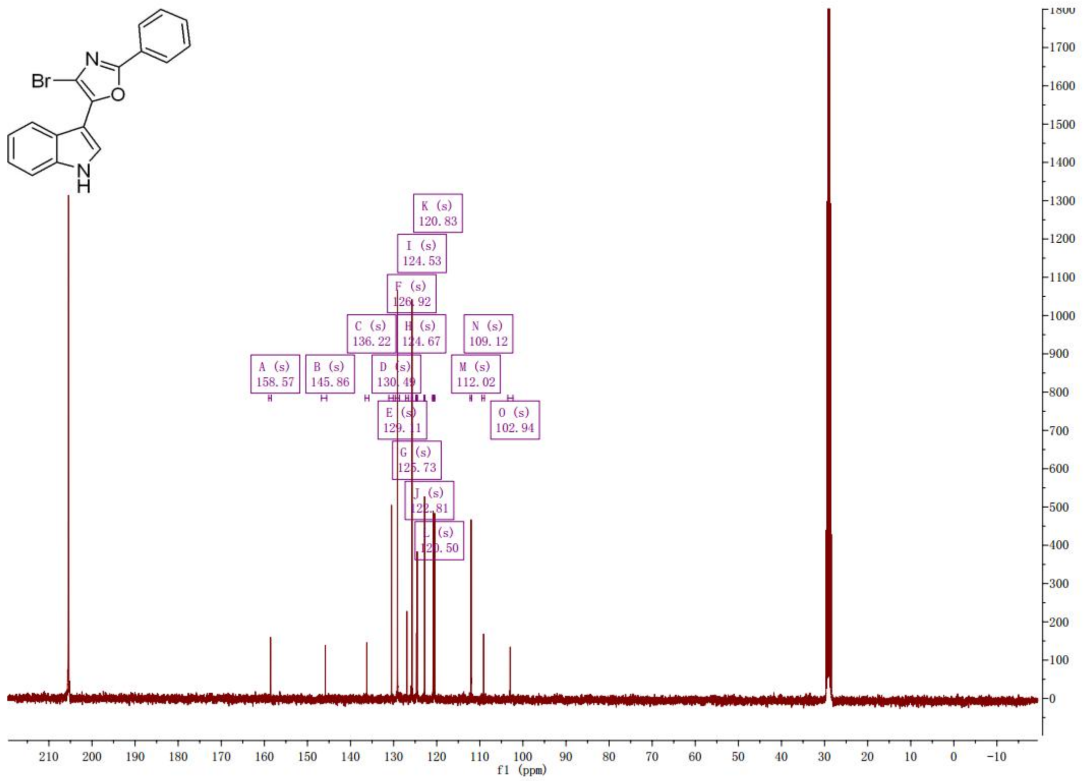

Compound 5g

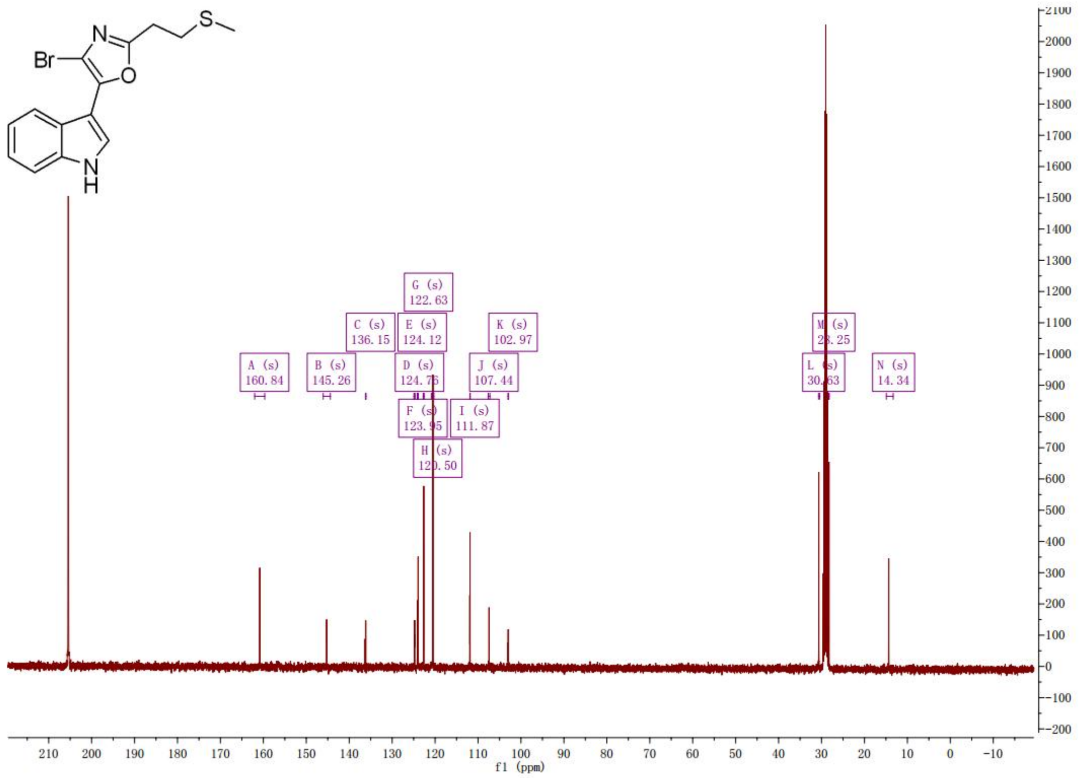

Compound 8a

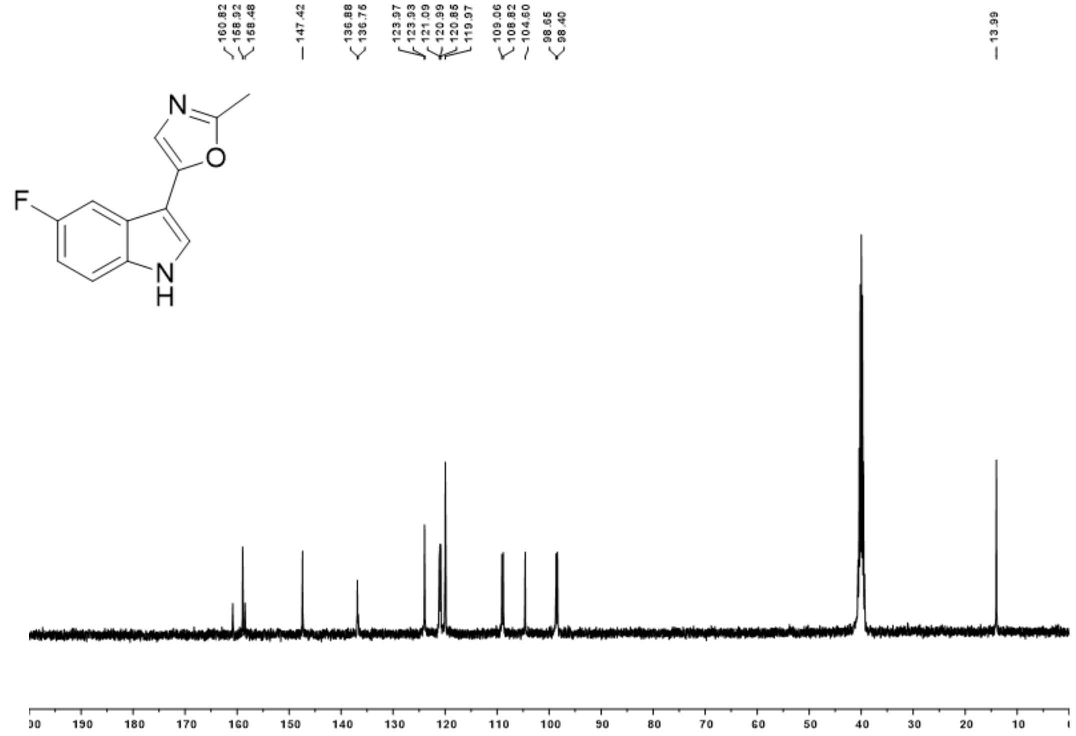

Compound 8b

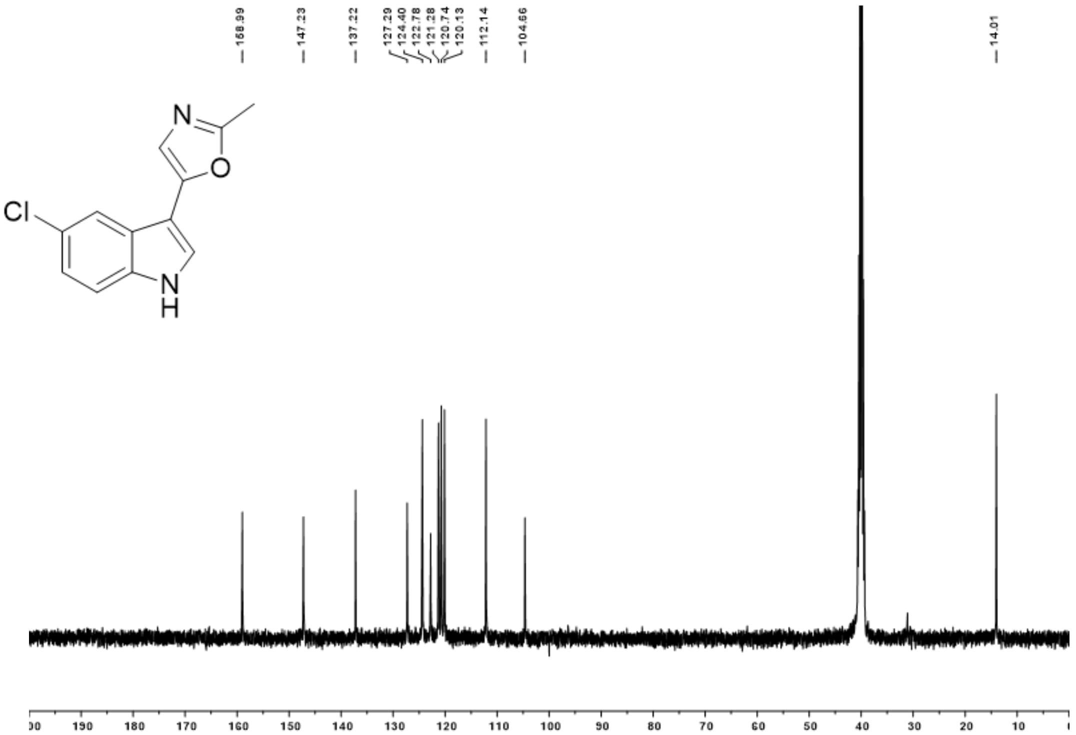

Compound 8c

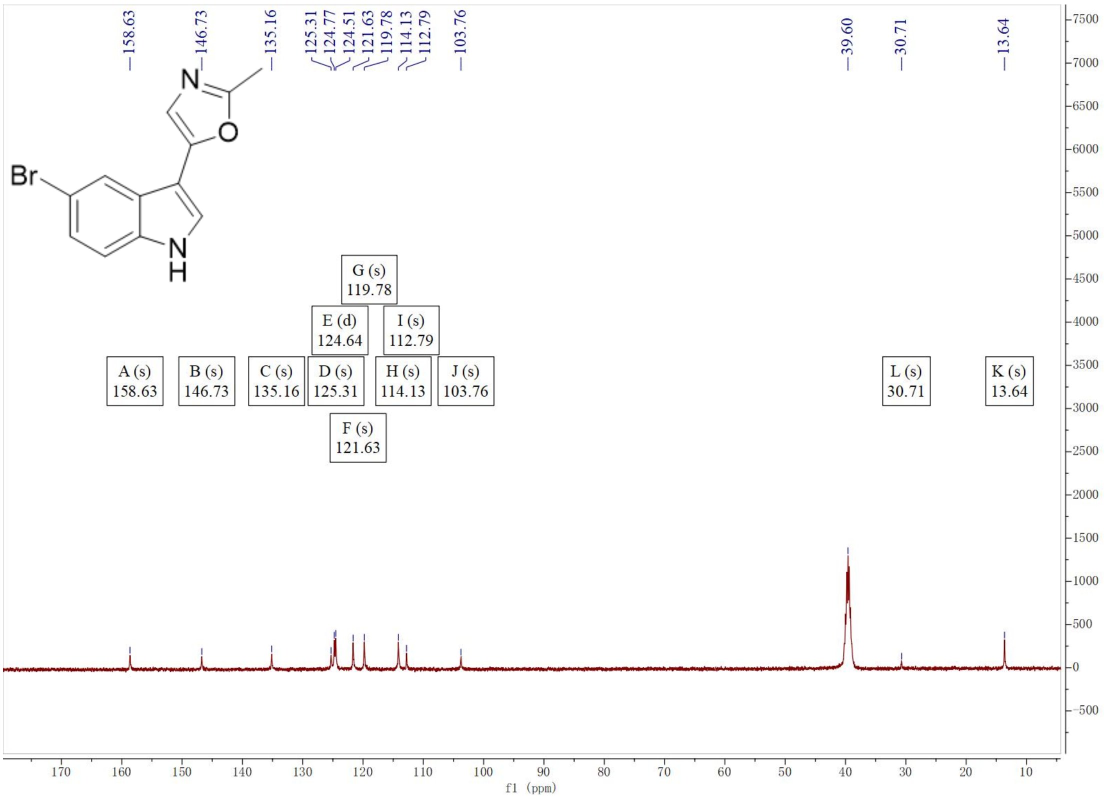

Compound 8d

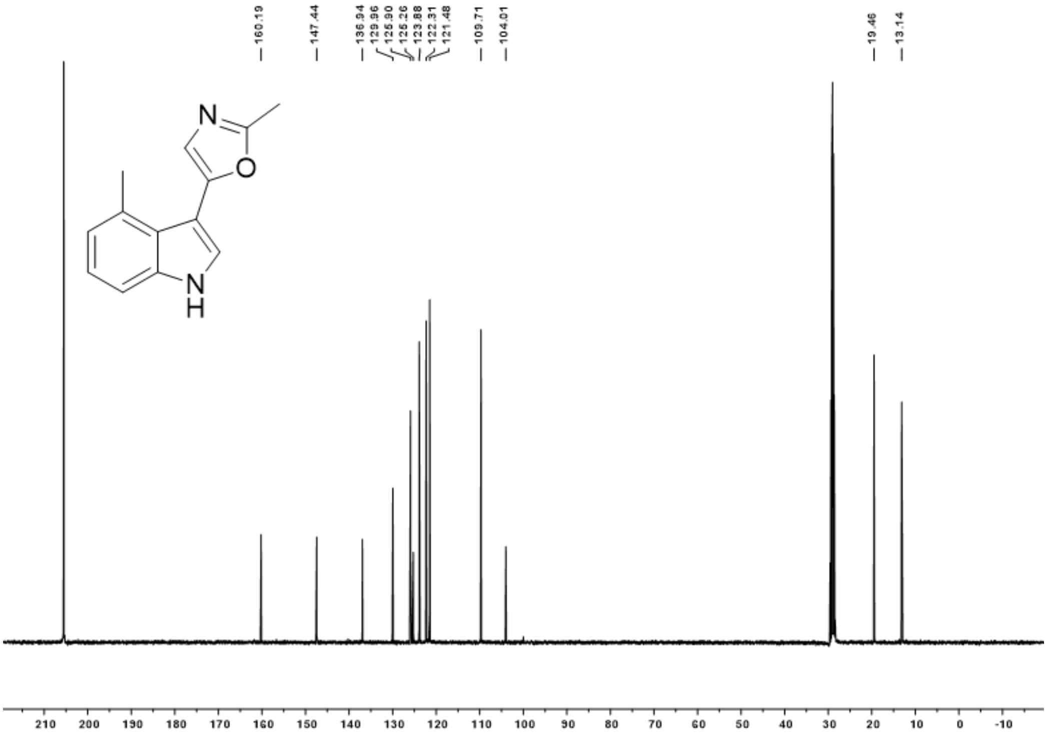

Compound 8e

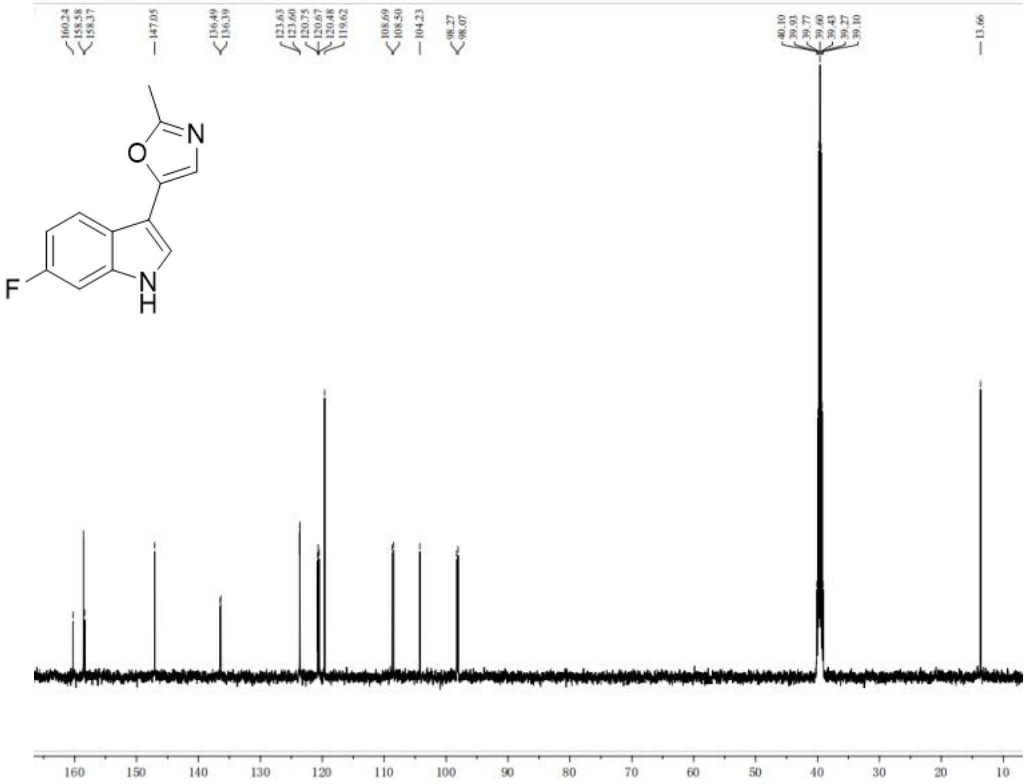

Compound 8f

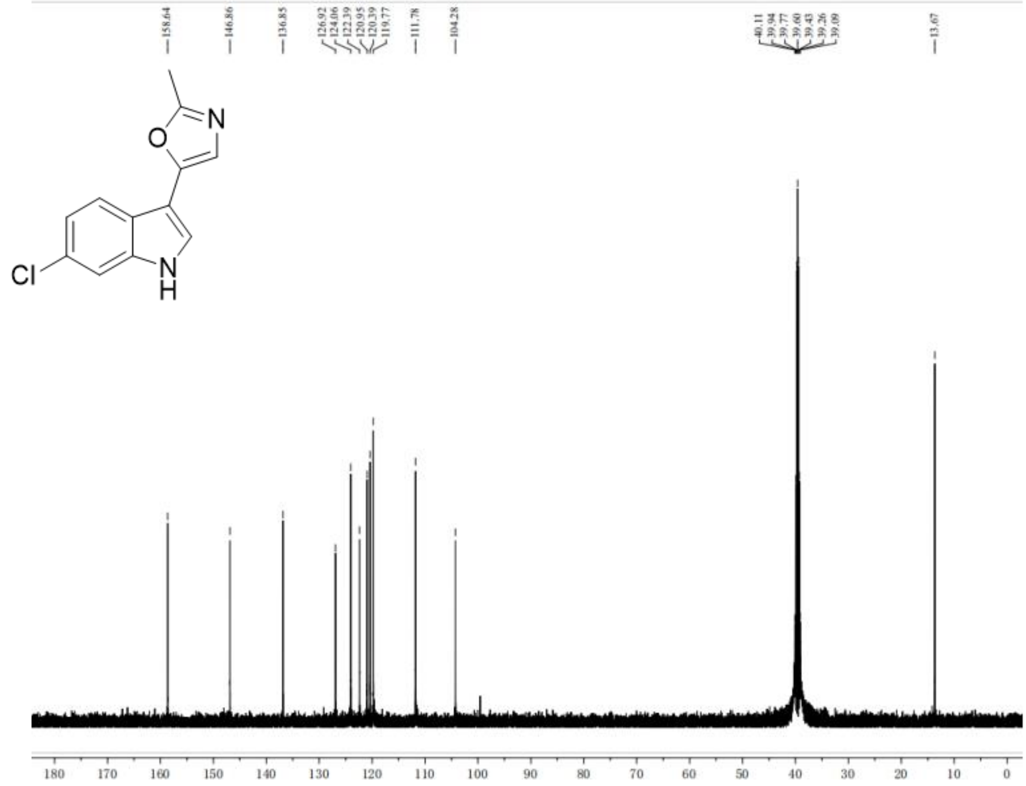

Compound 8g

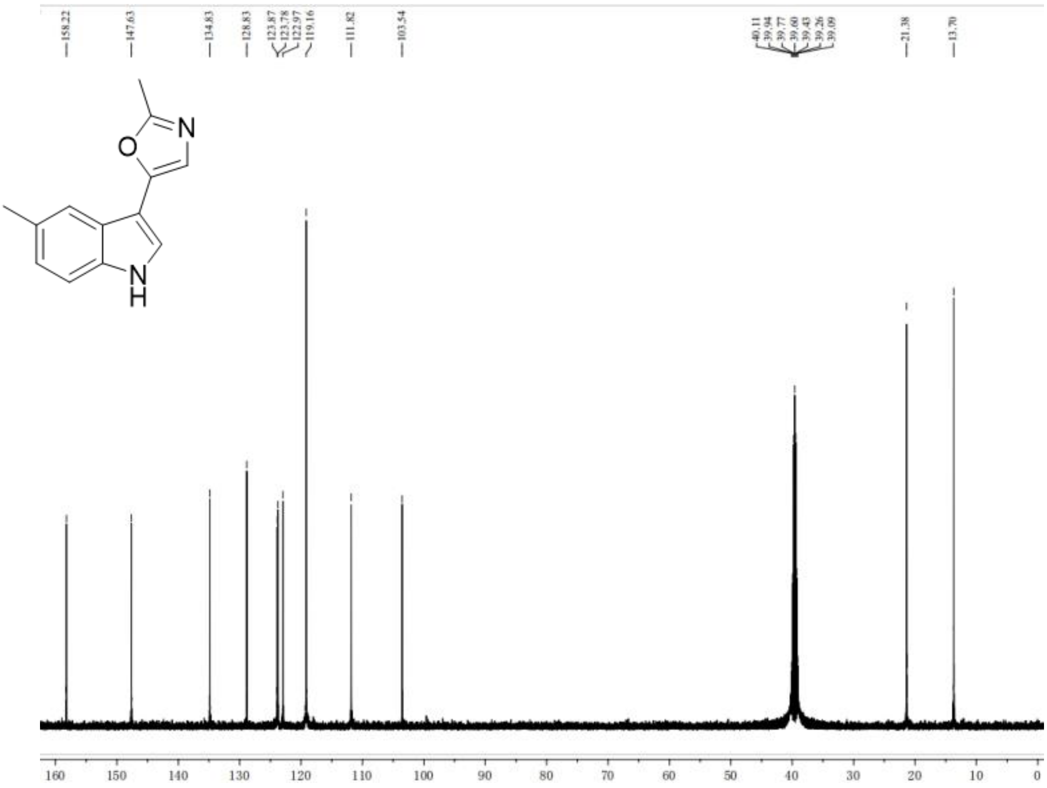

Compound 8h

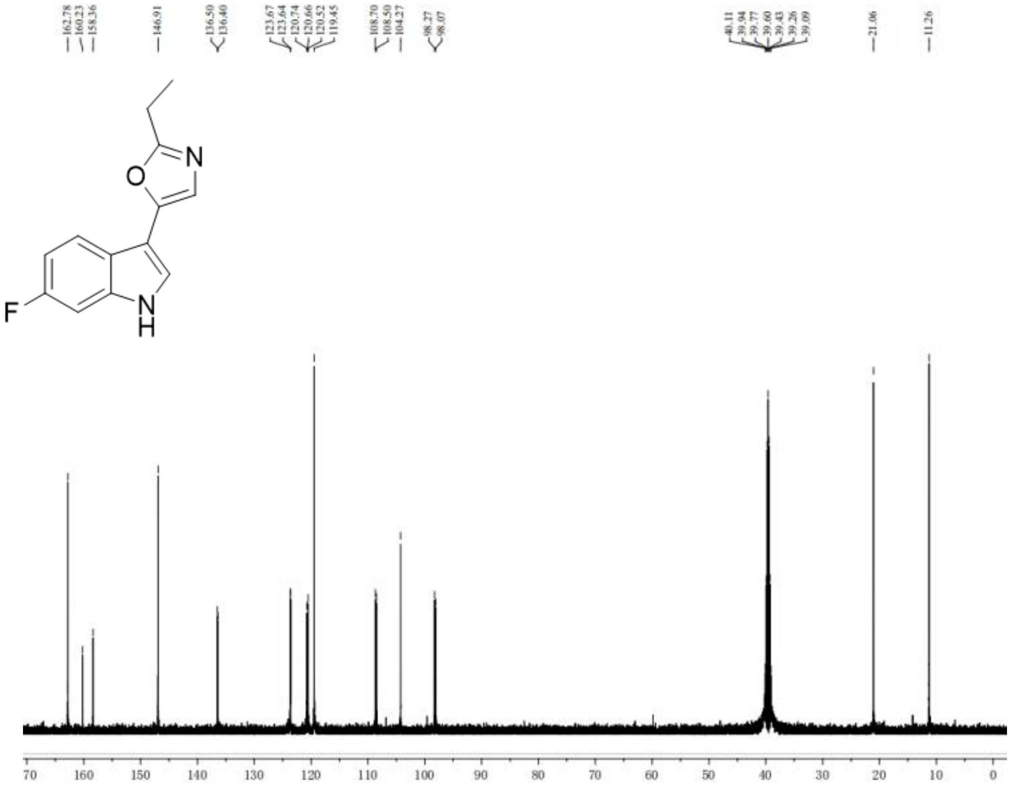

Compound 8i

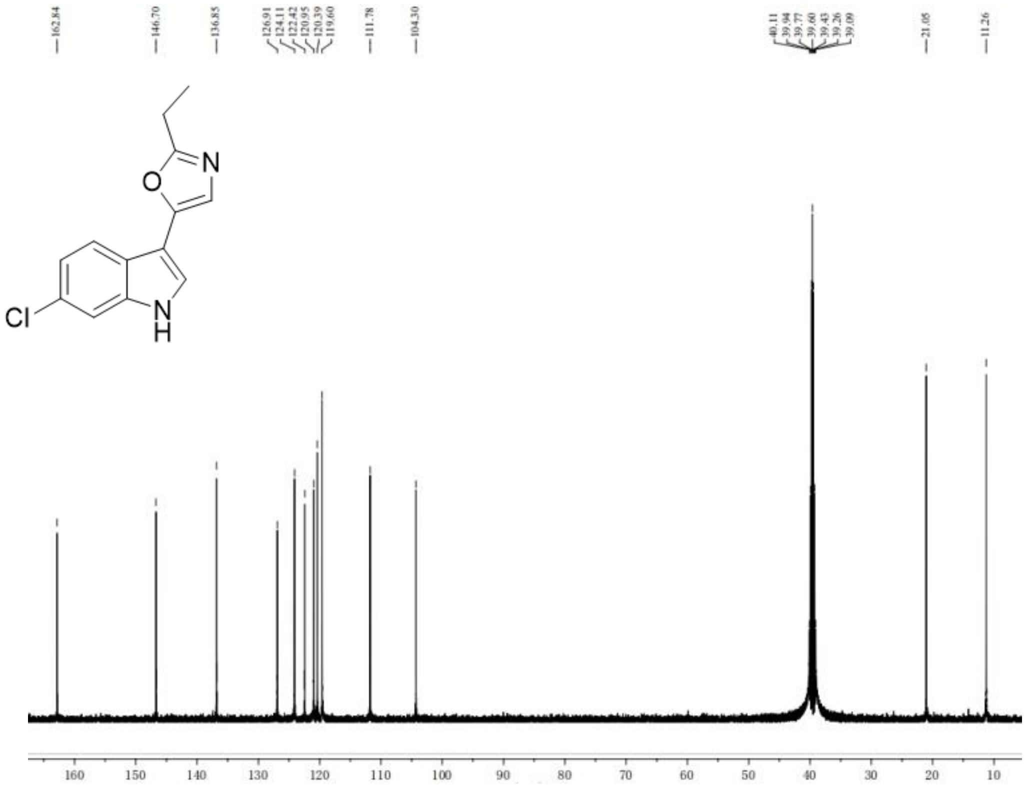

Compound 8j

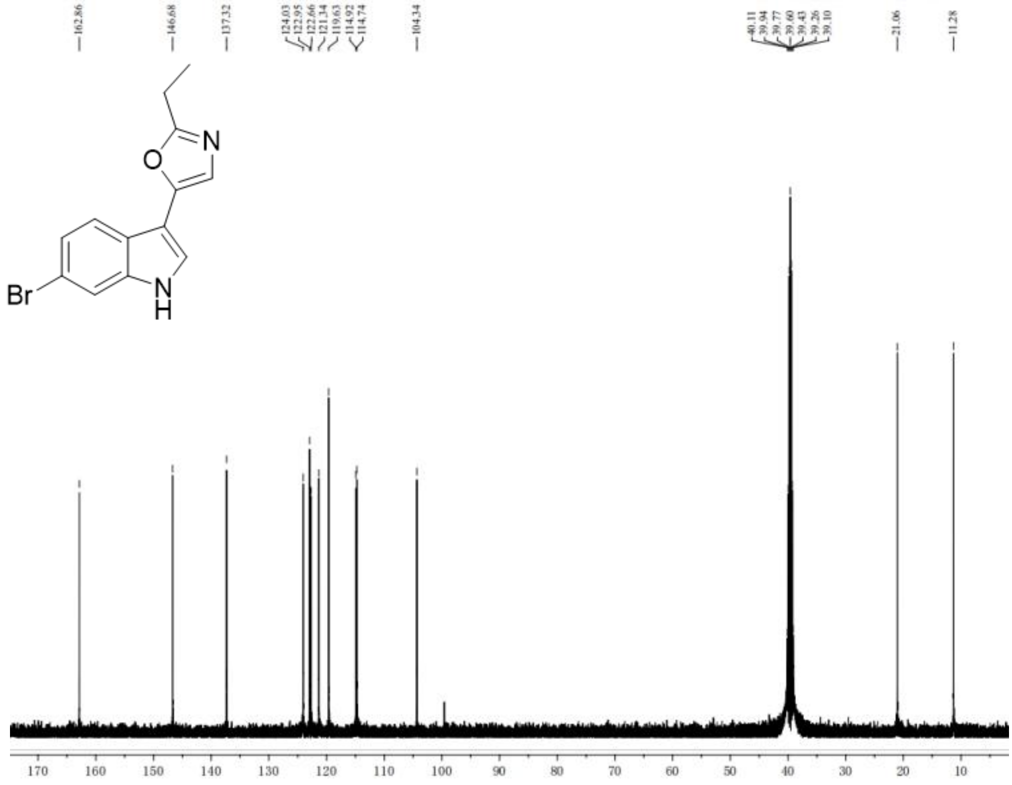

Compound 8k

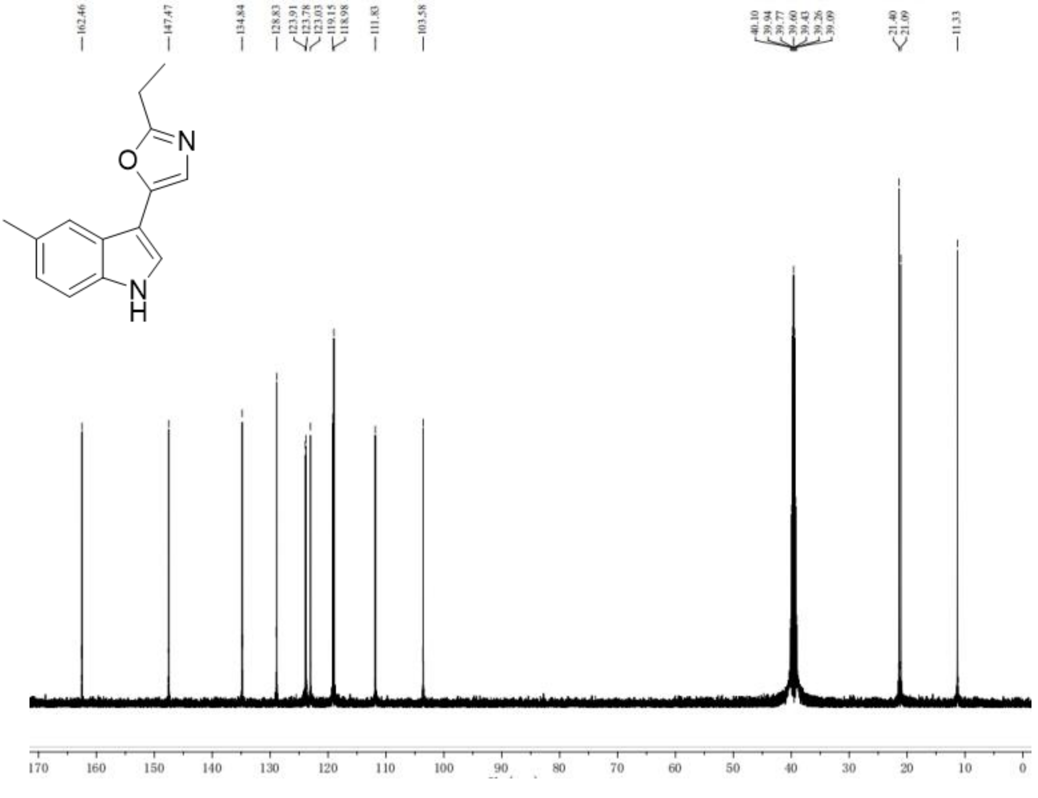

Compound 9a

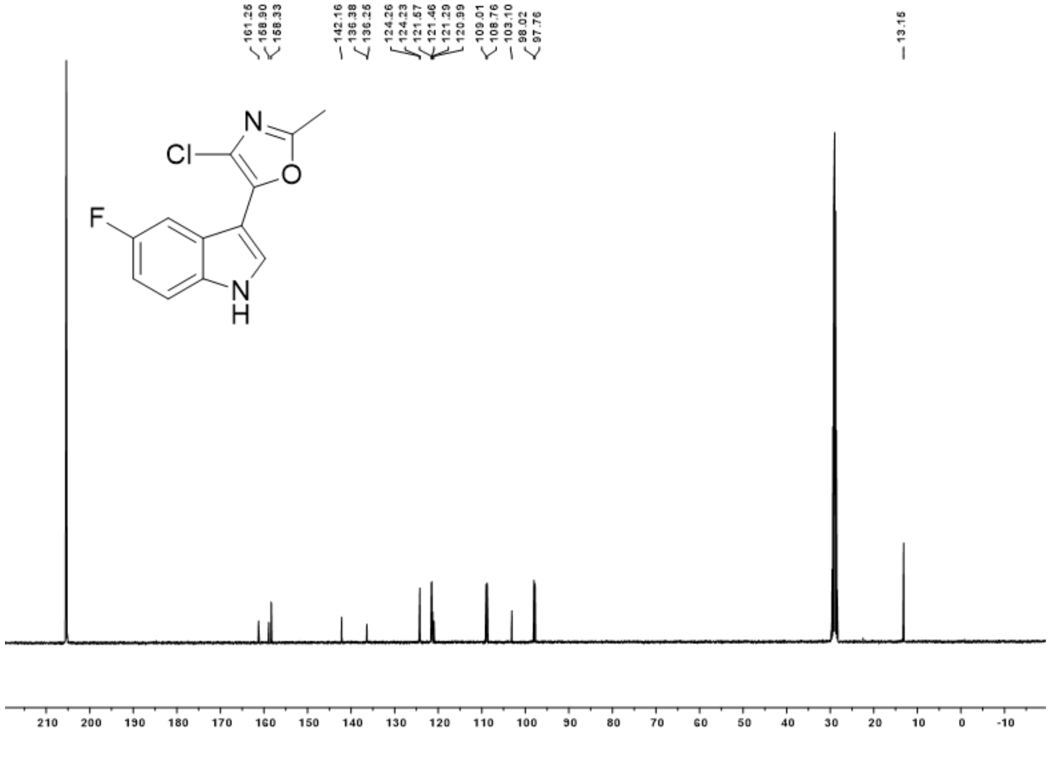

Compound 9b

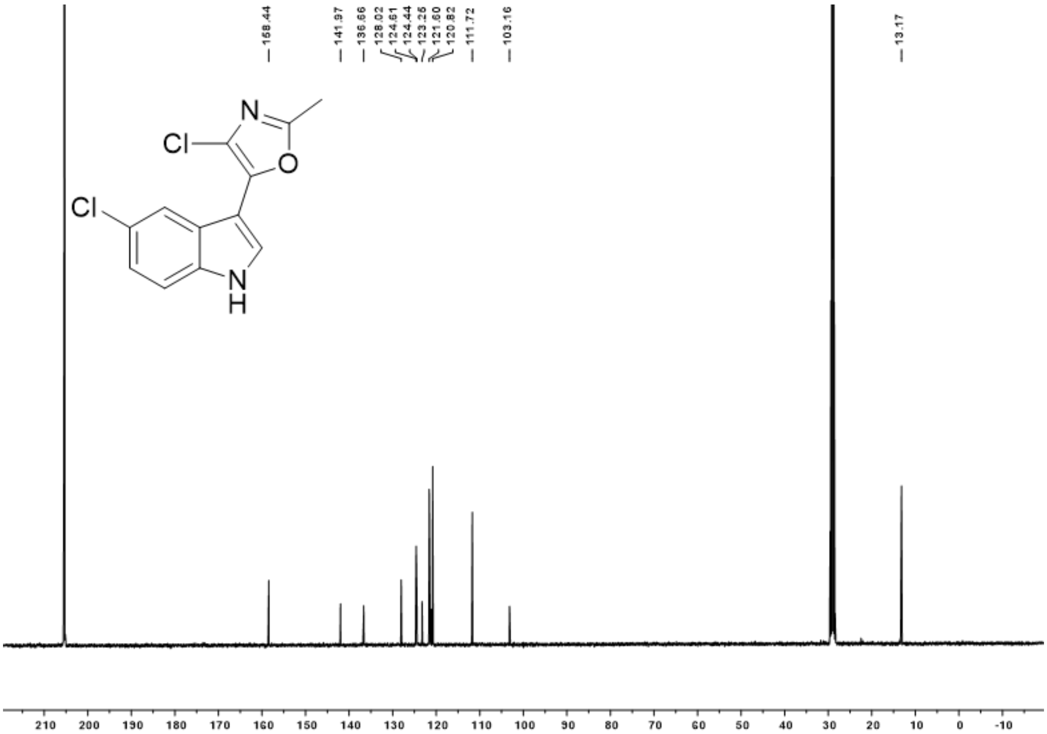

Compound 9c

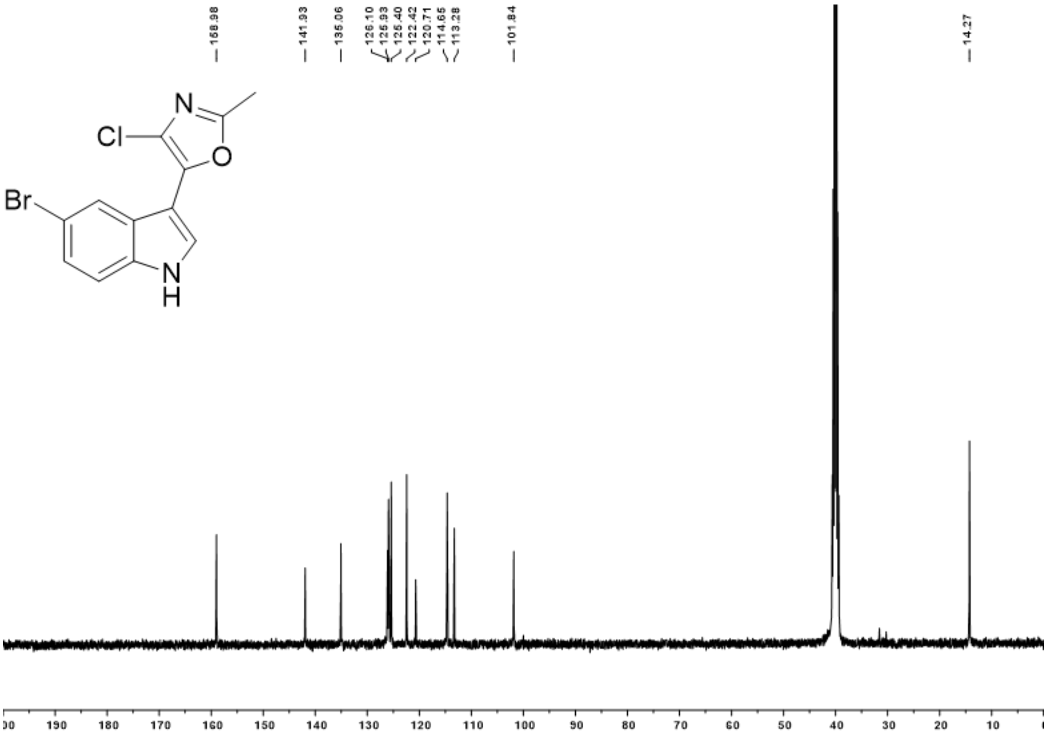

Compound 9d

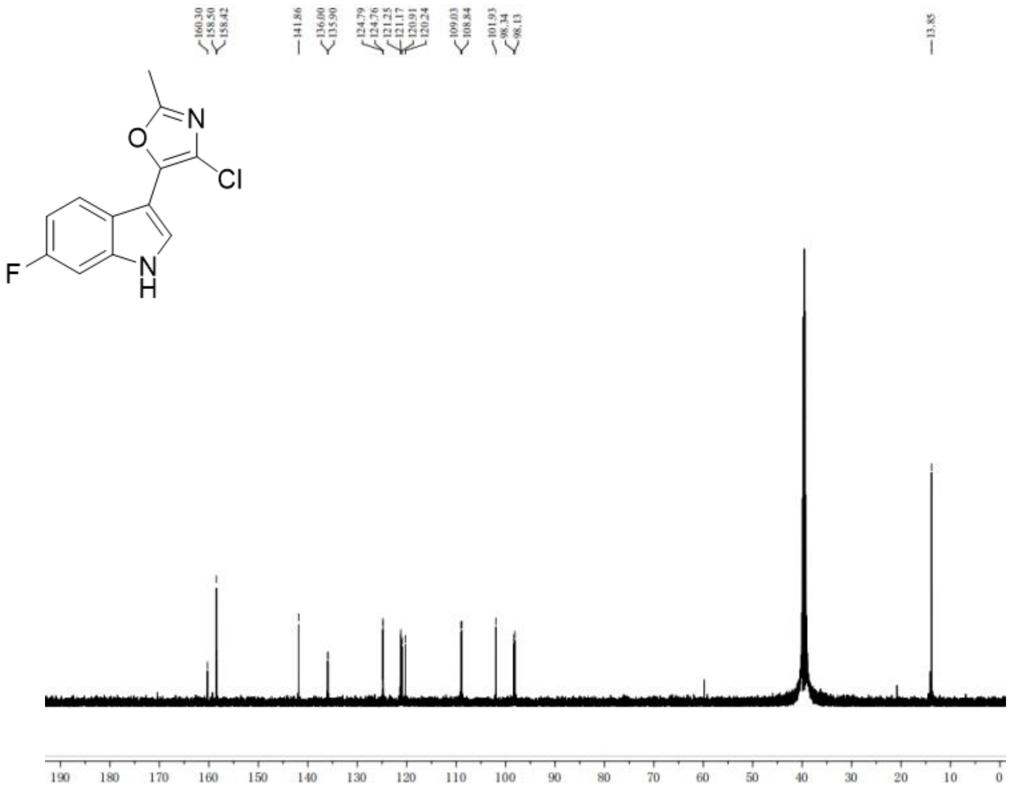

Compound 9e

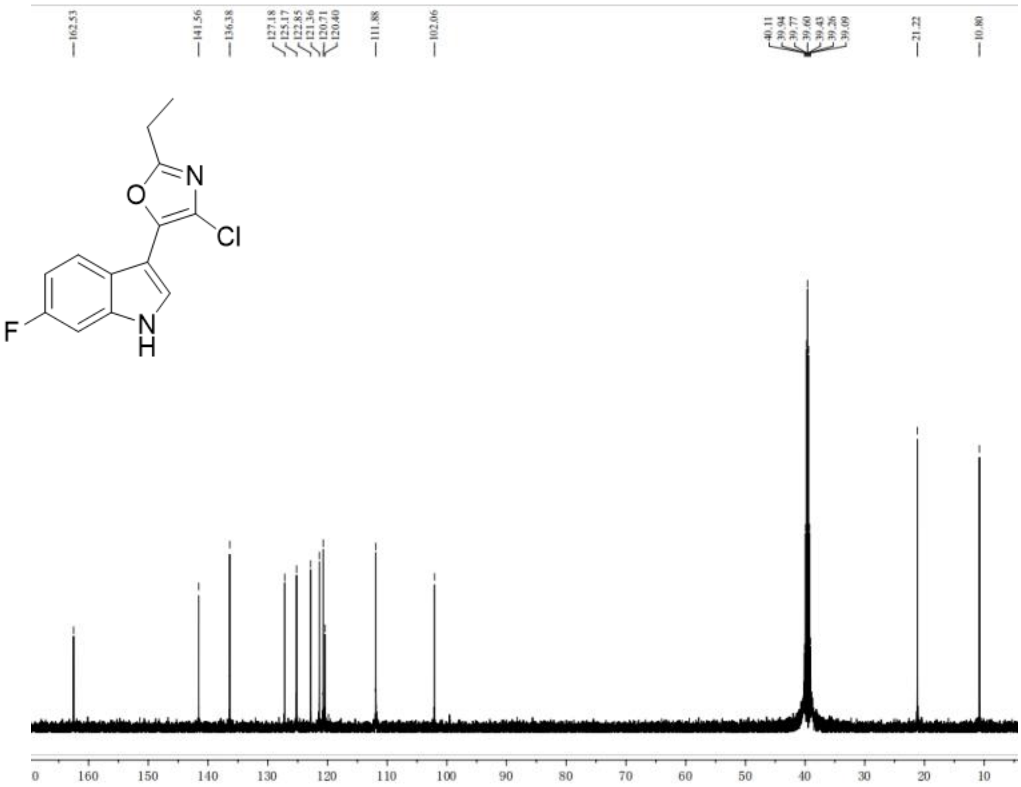

Compound 9f

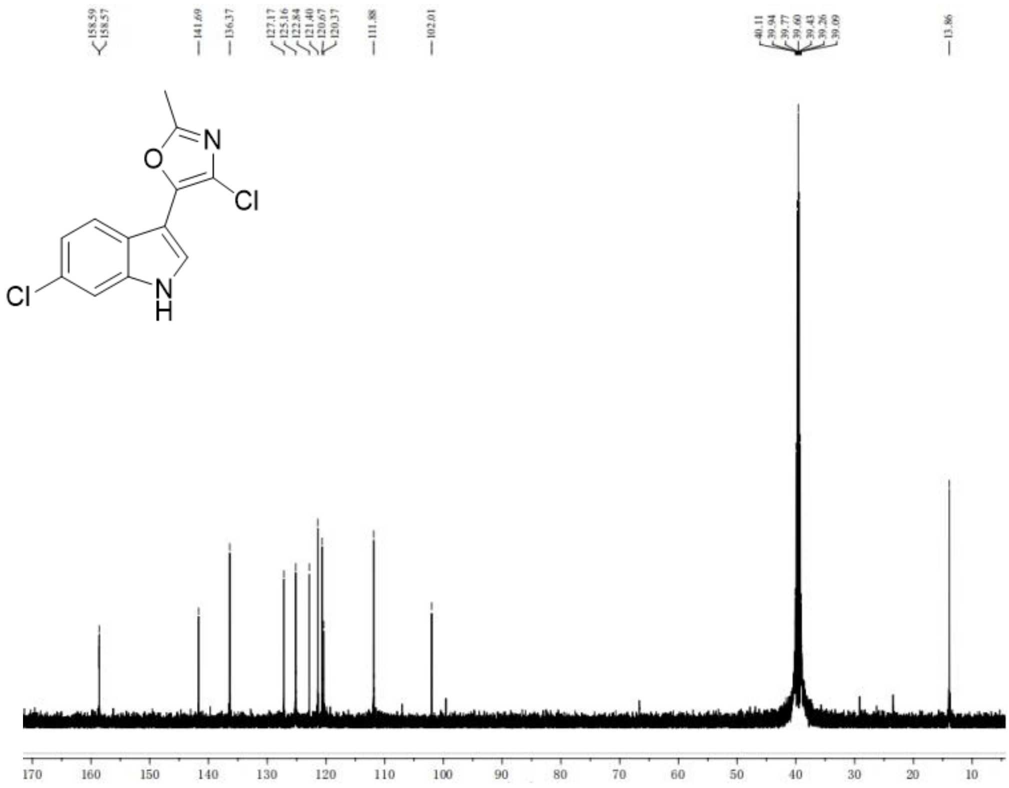

Compound 9g

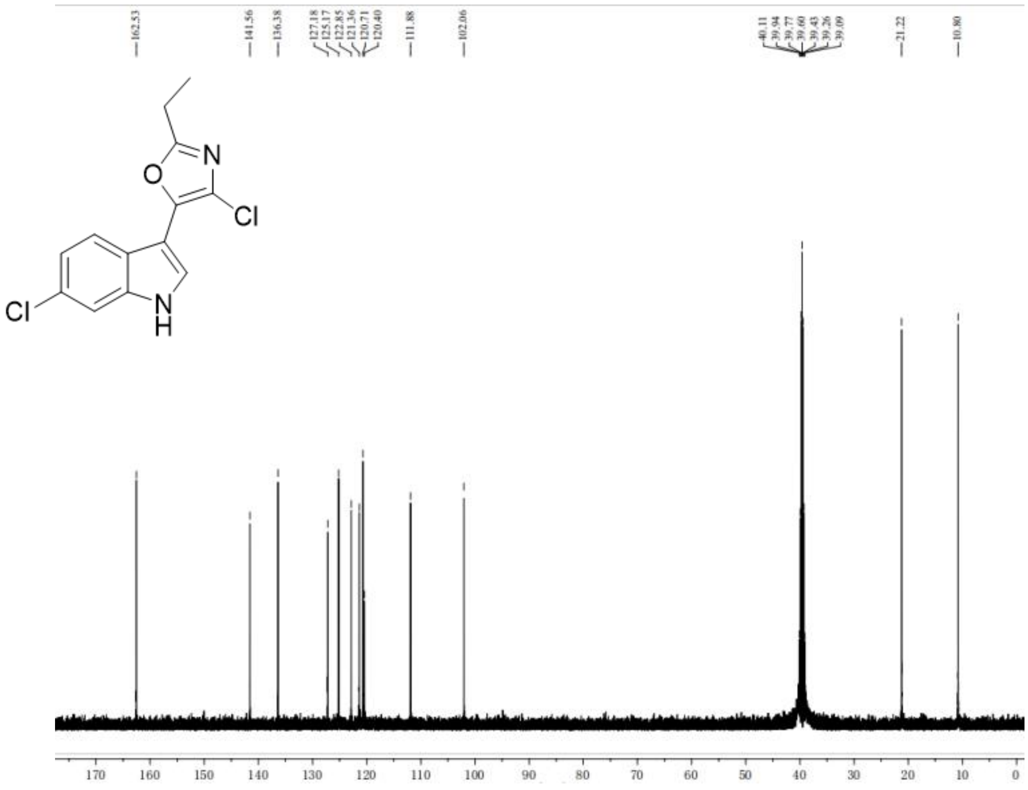

Compound 9h

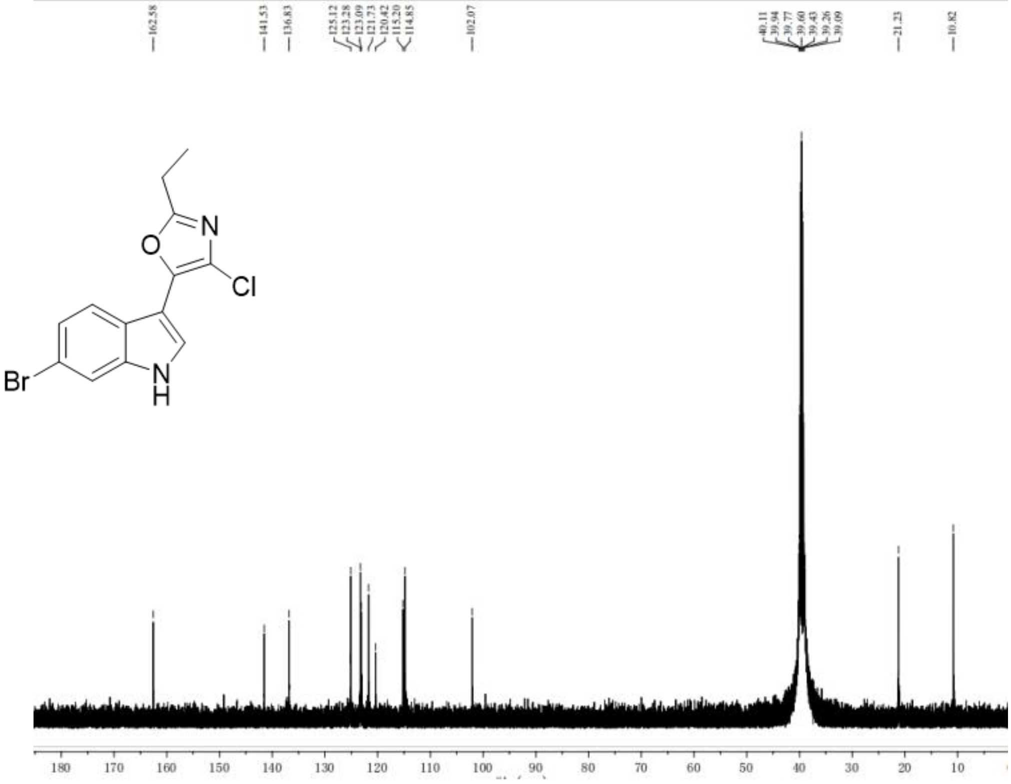

Compound 9i

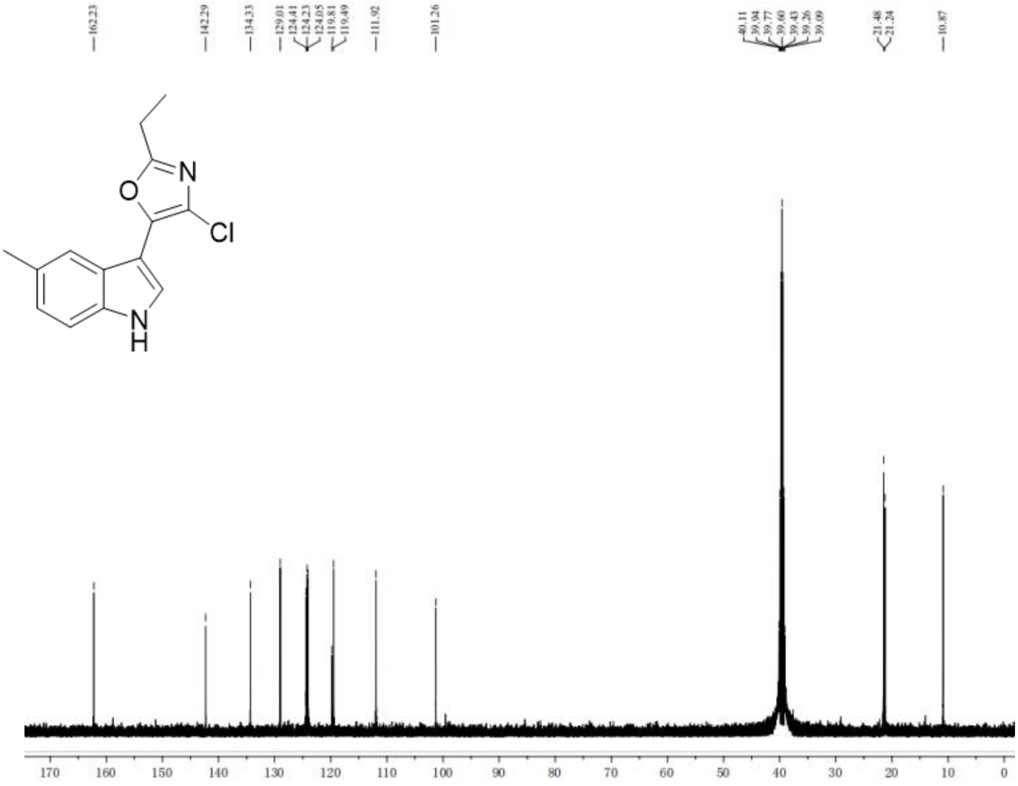

Compound 10a

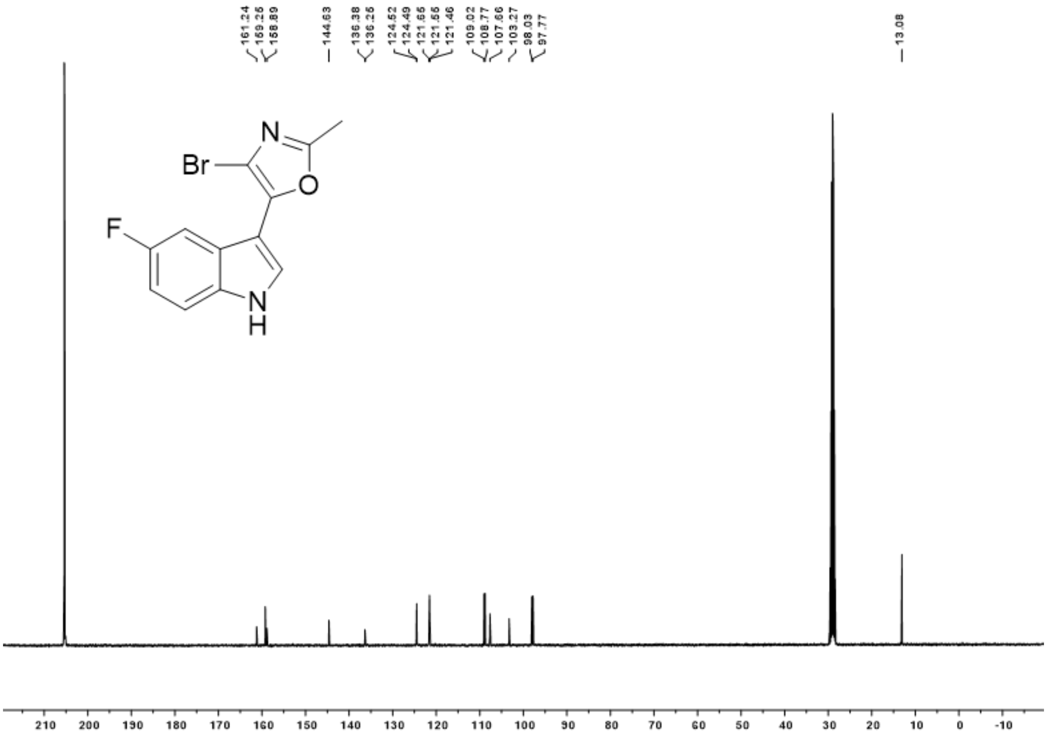

Compound 10b

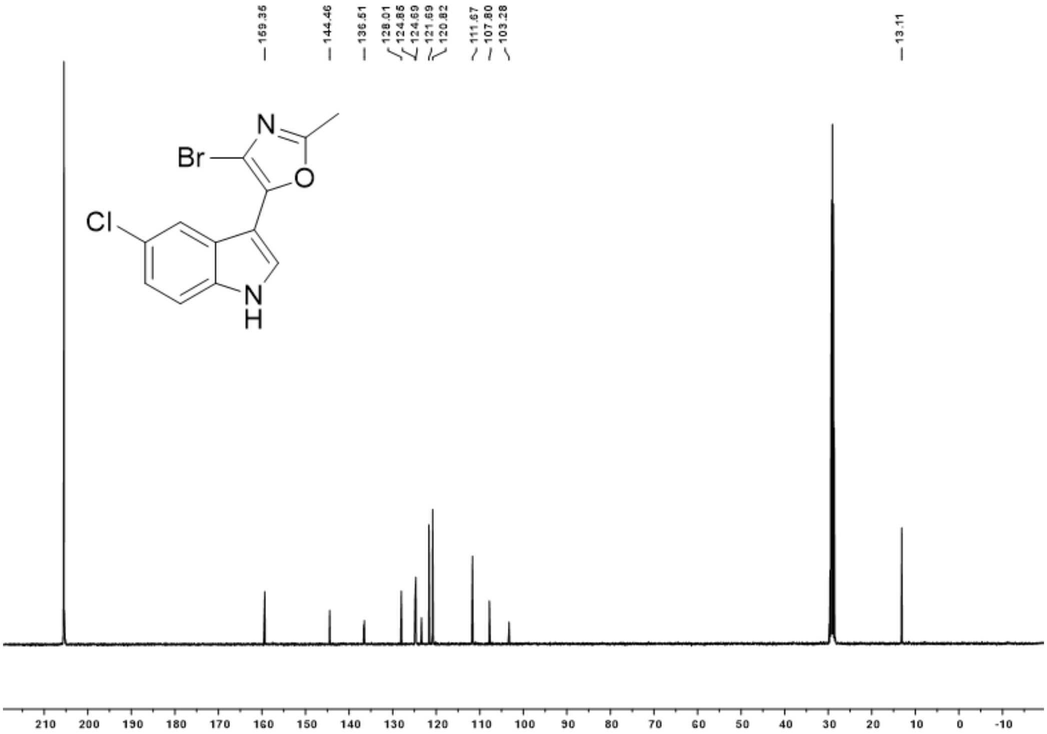

Compound 10c

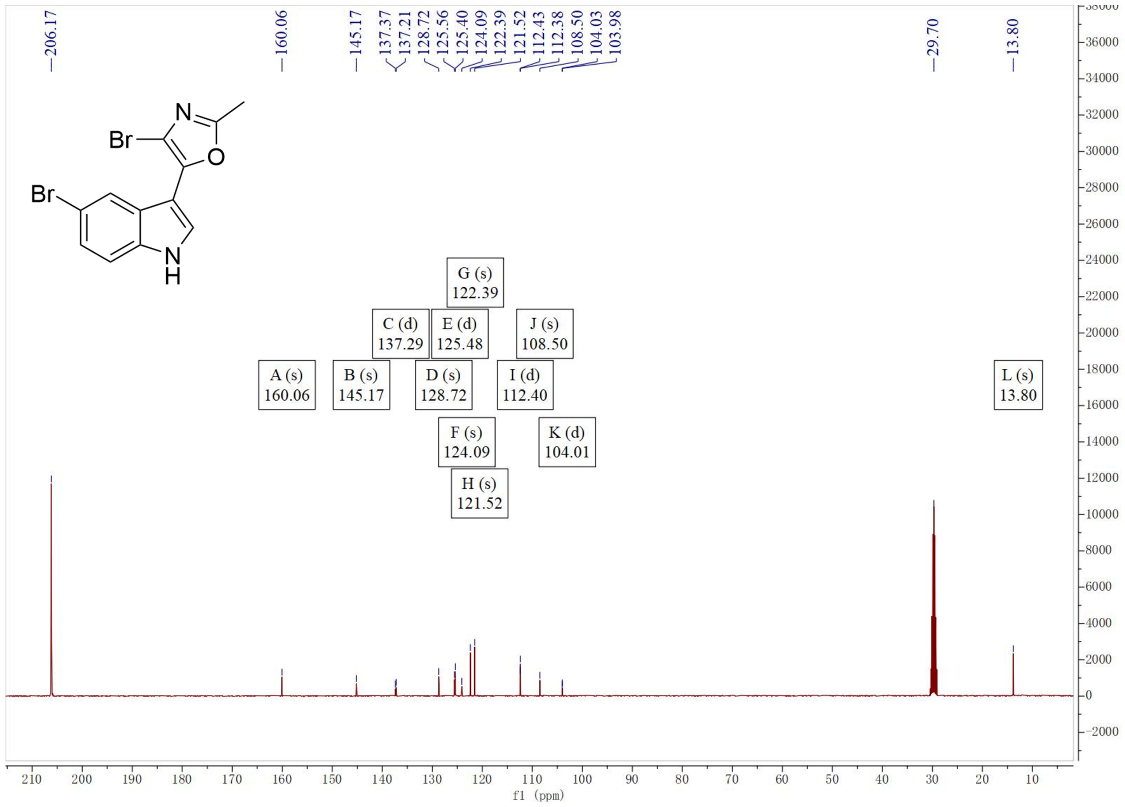

Compound 10d

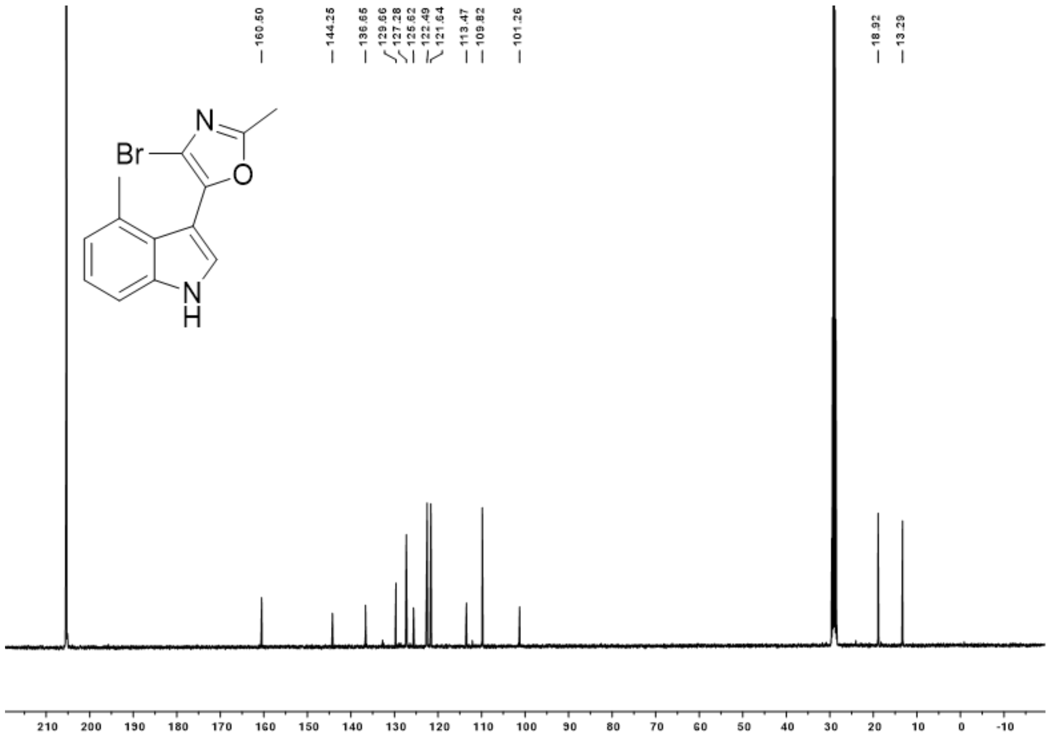

Compound 10e

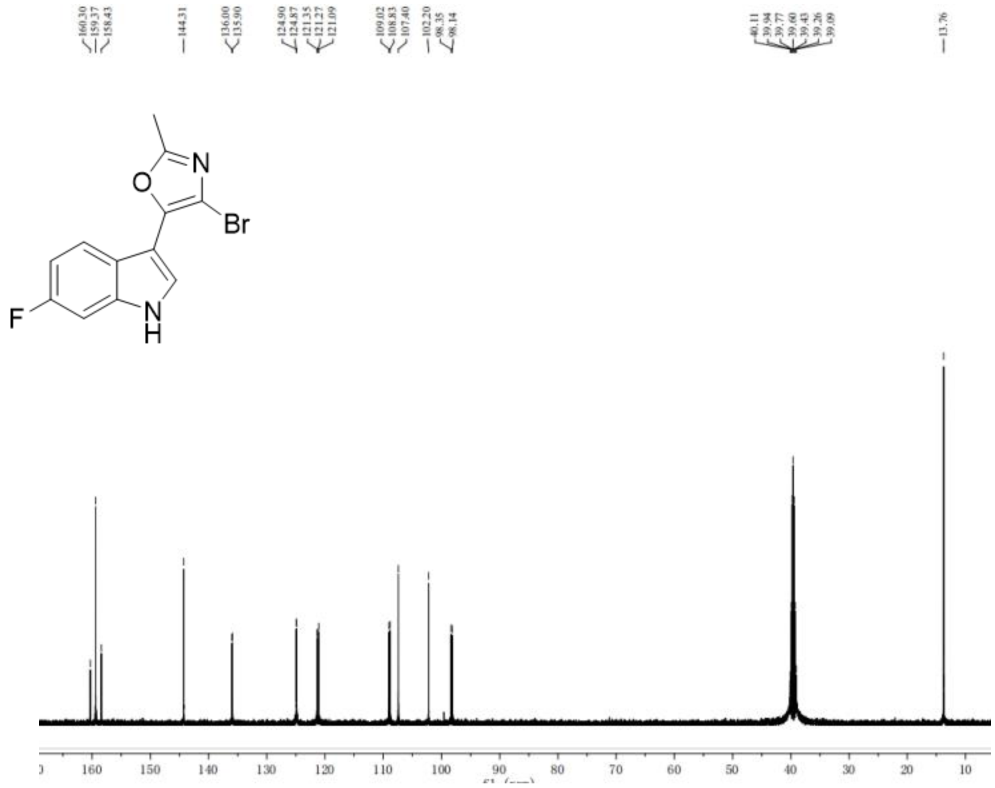

Compound 10f

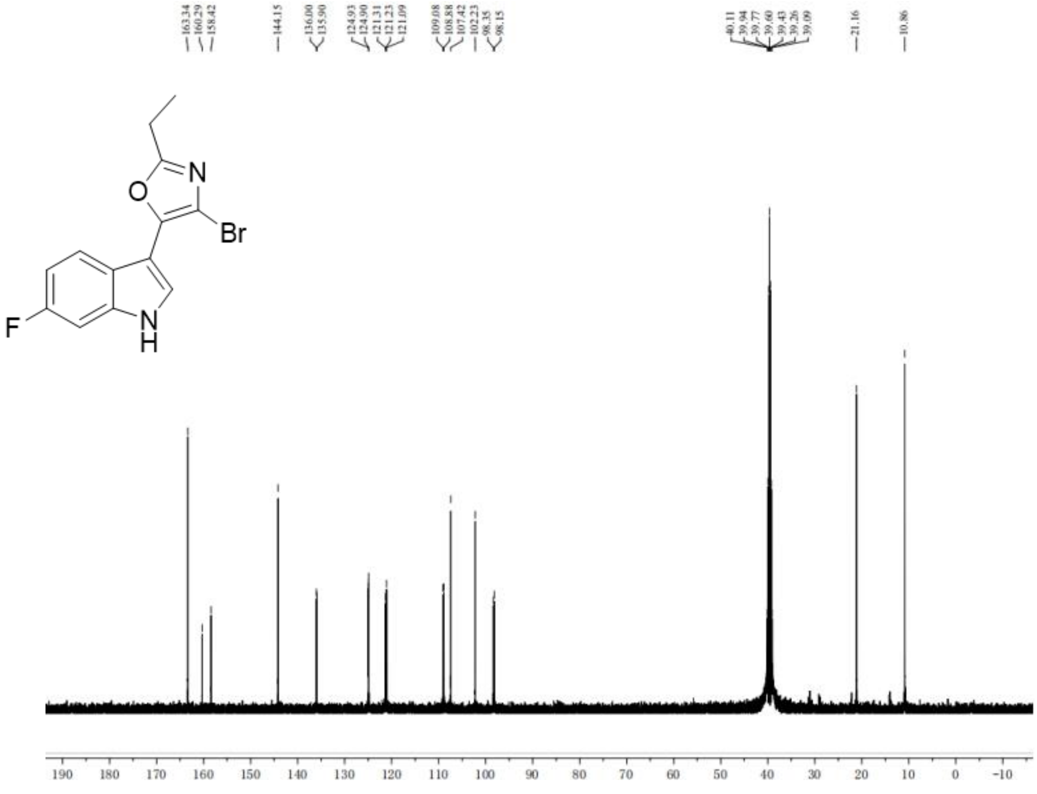

Compound 10g

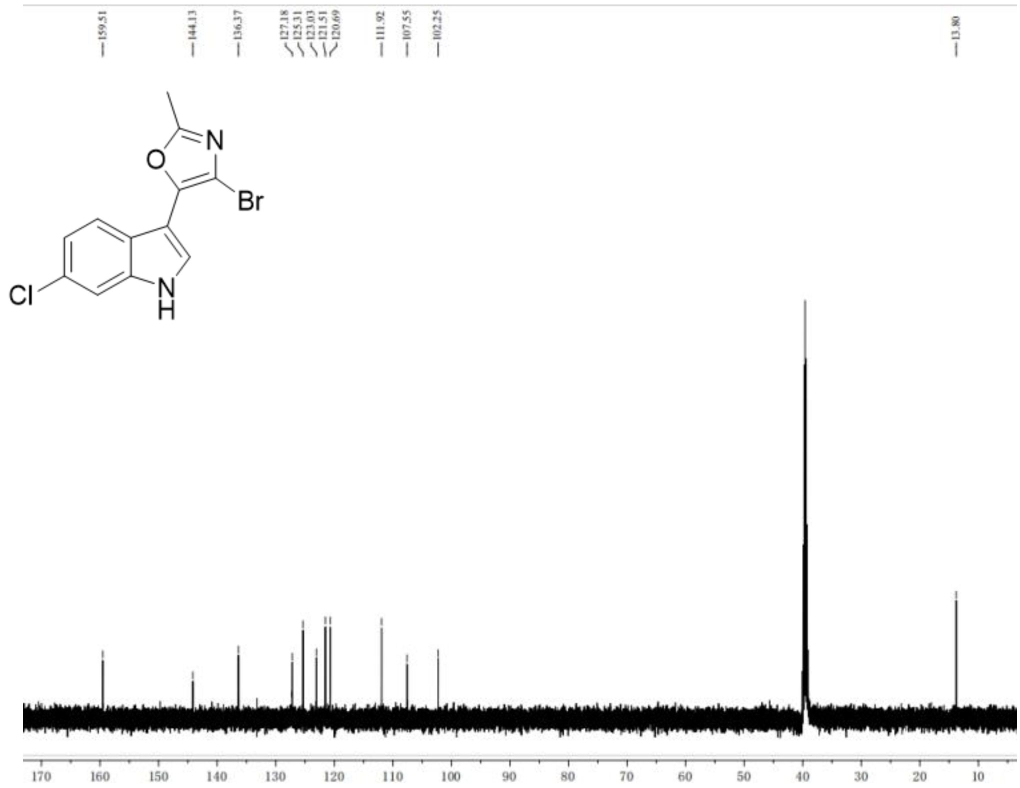

Compound 10h

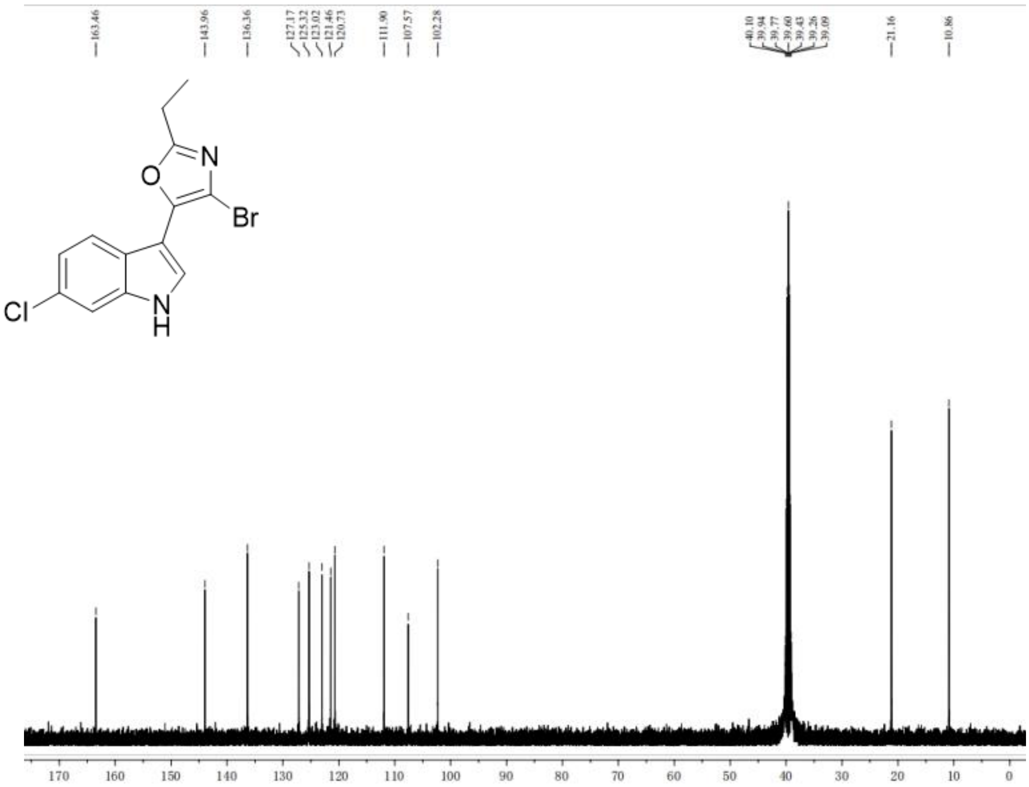

Compound 10i

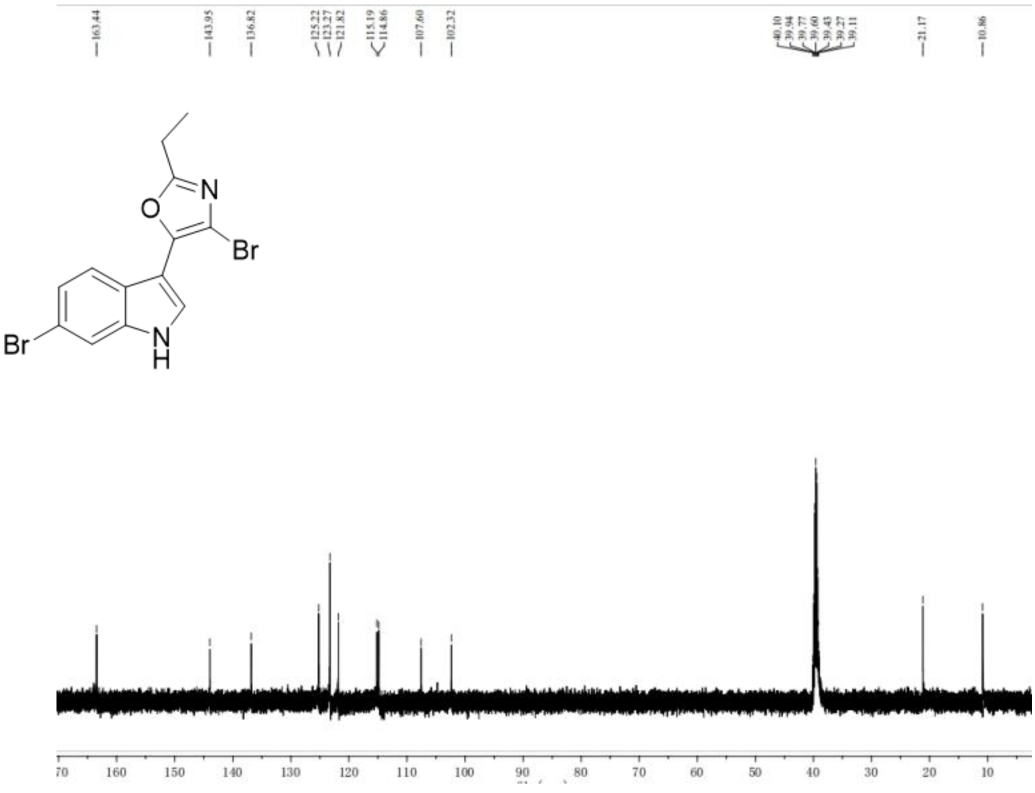

4. HR-MS

Compound 3a

Spectrum from 3.wiff (sample 1) - Sample004, +TOF MS (90 - 1000) from 2.084 to 2.091 min

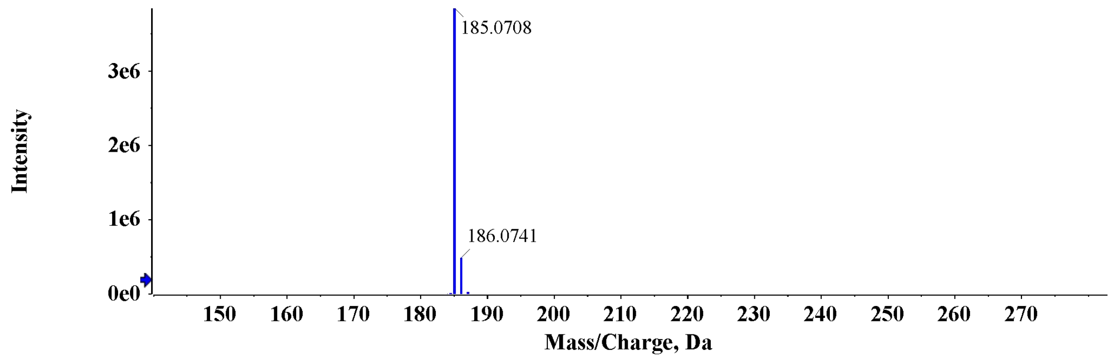

Compound 3b

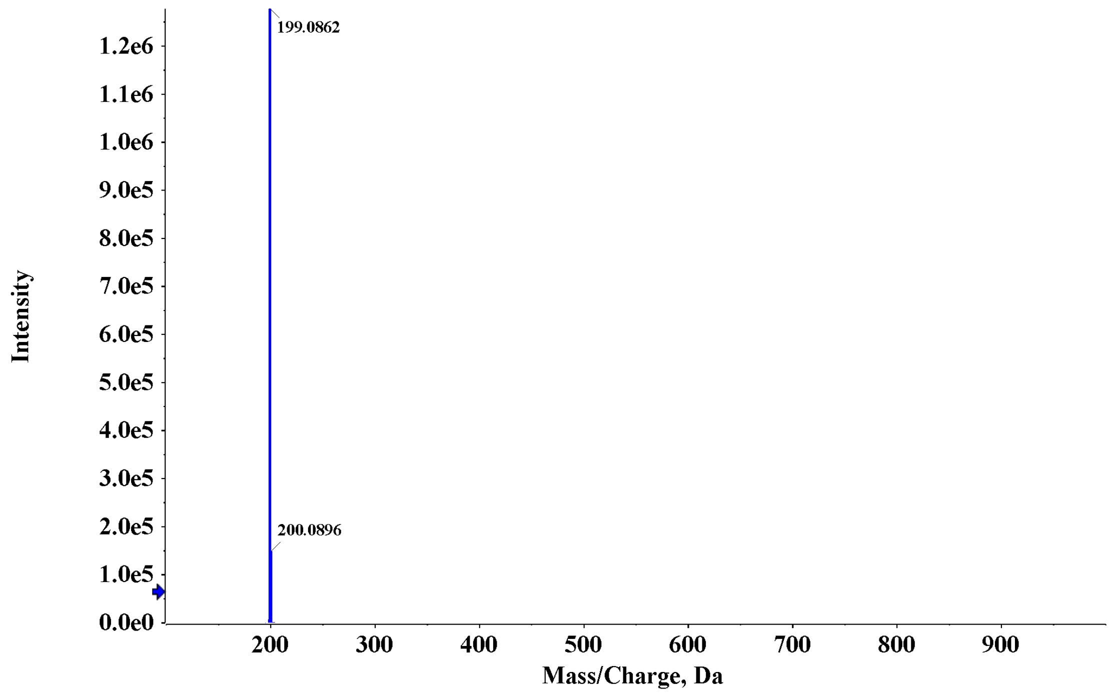

Compound 3c

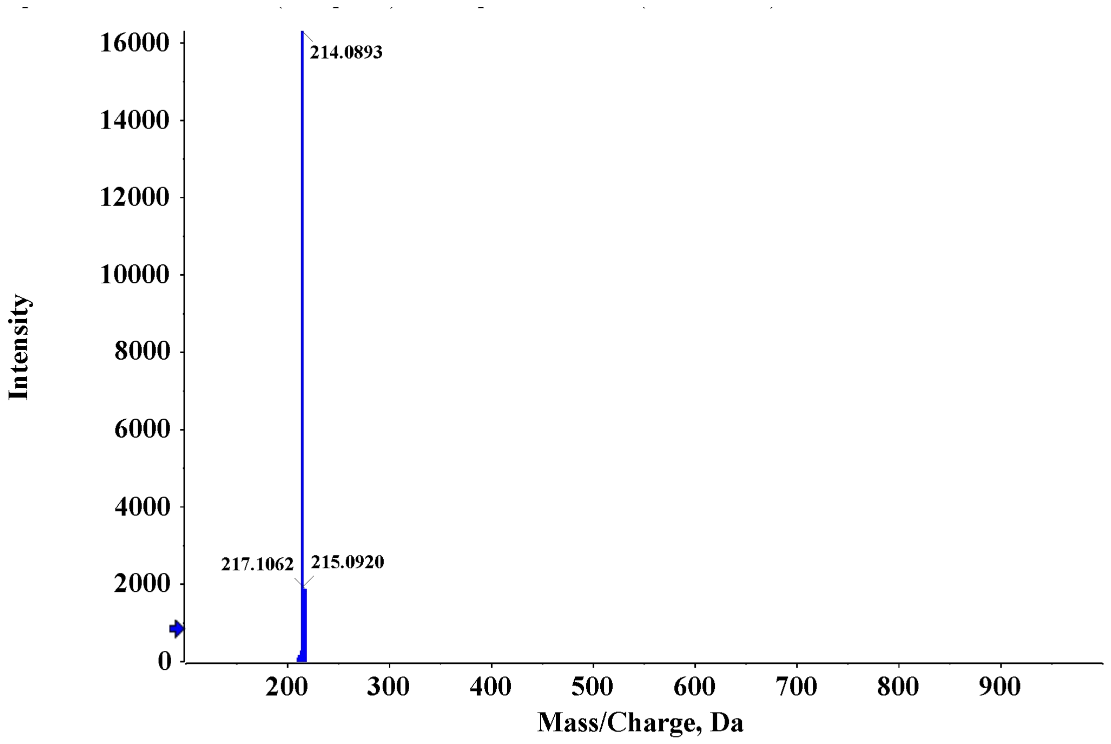

Compound 3d

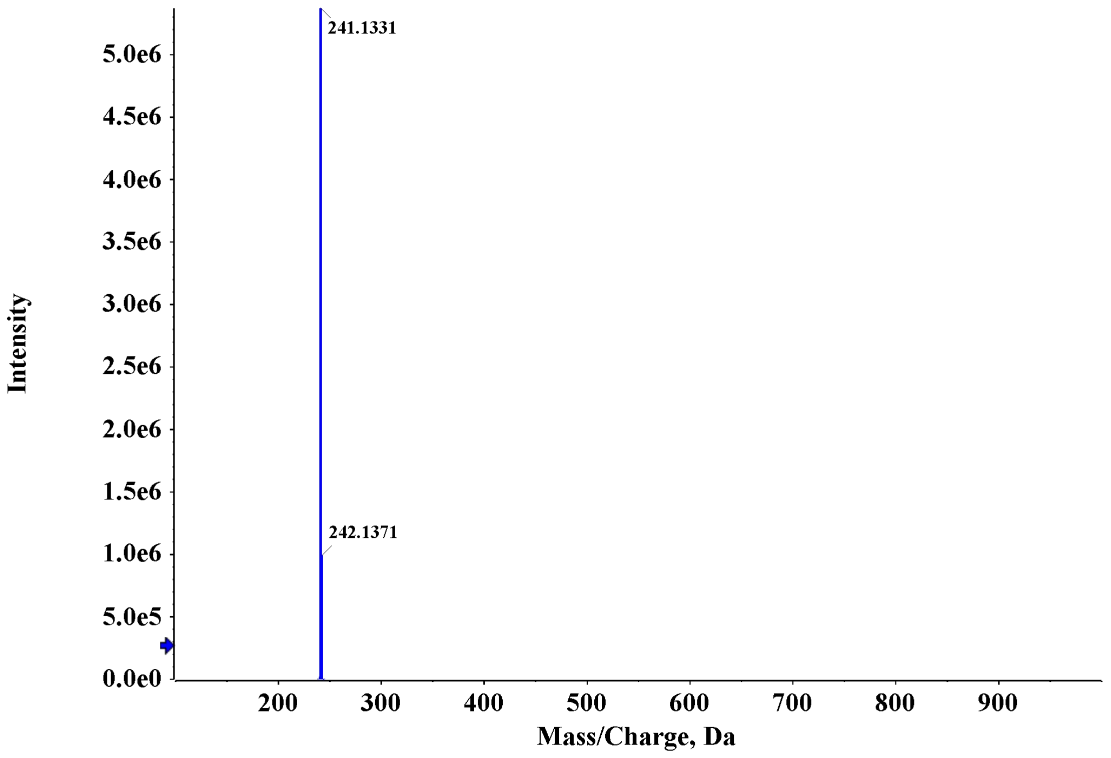

Compound 3e

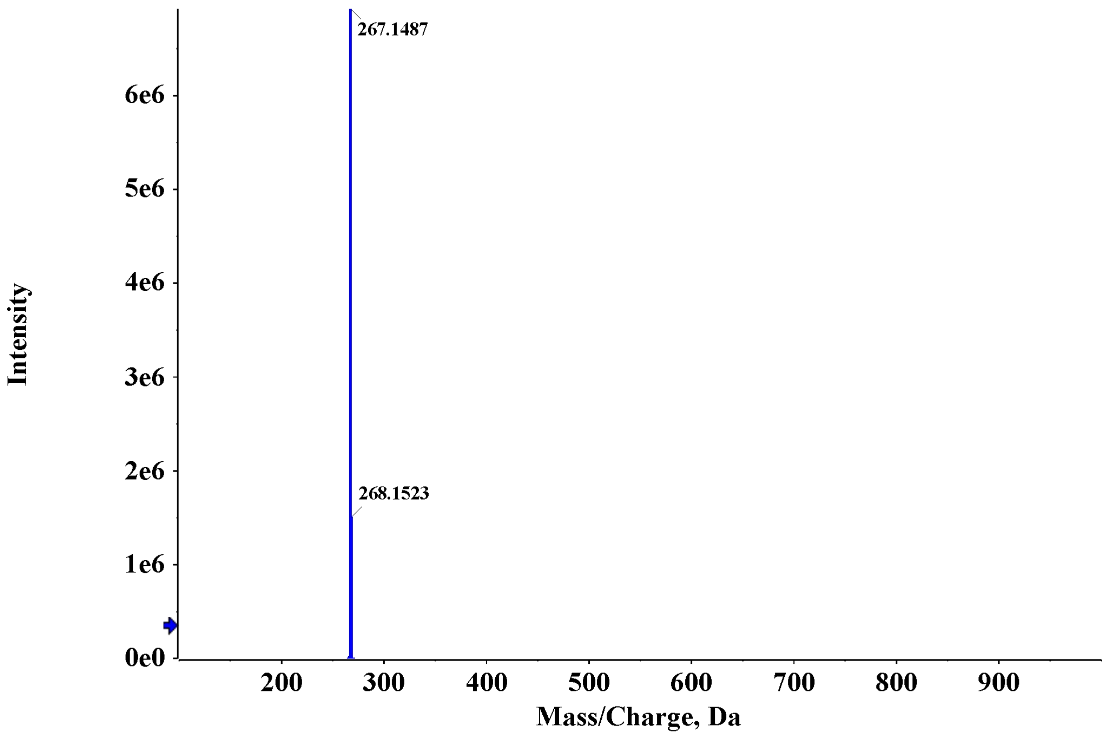

Compound 3f

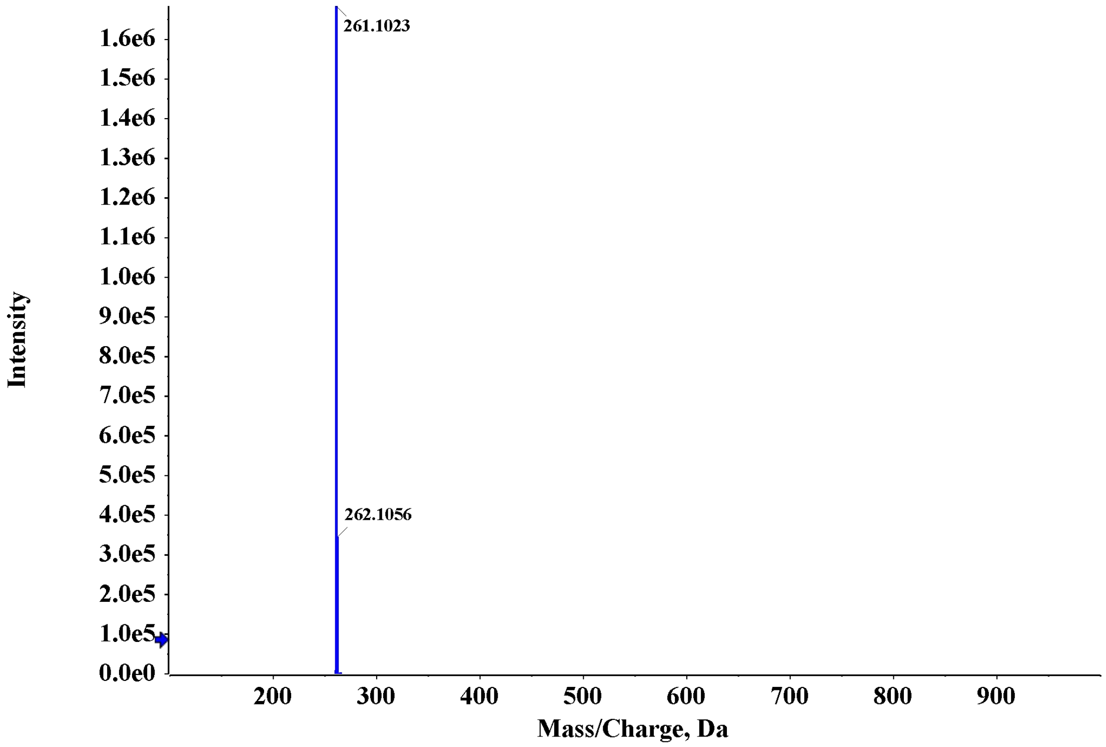

Compound 3g

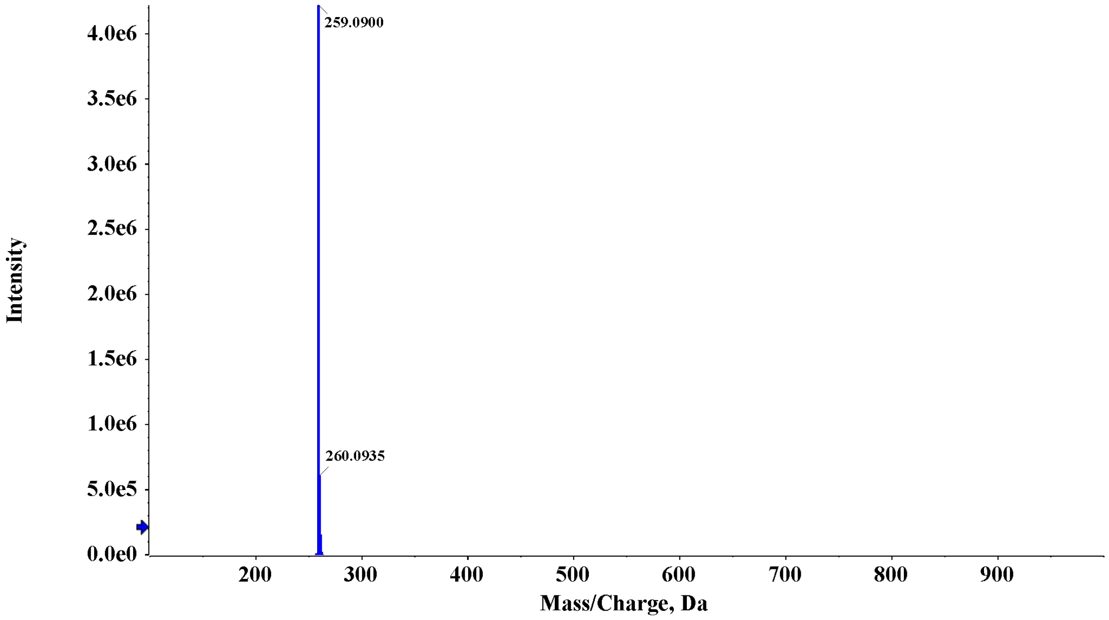

Compound 4a

Spectrum from 1.wiff (sample 1) - Sample002, +TOF MS (90 - 1000) from 2.118 to 2.126 min

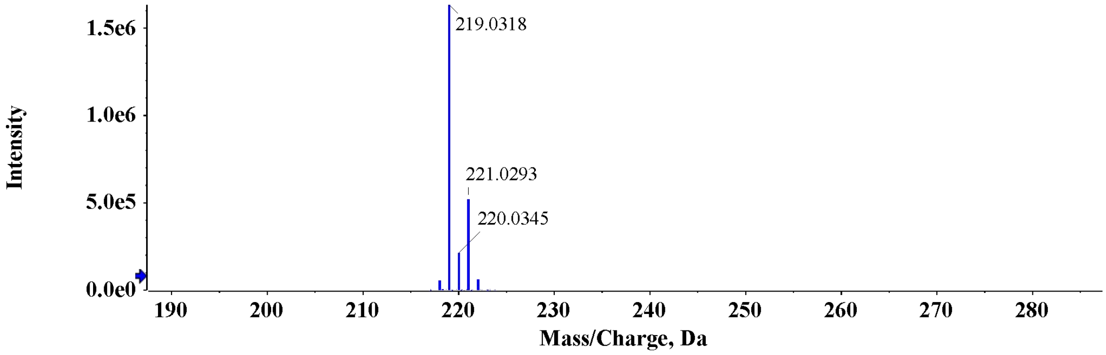

Compound 4b

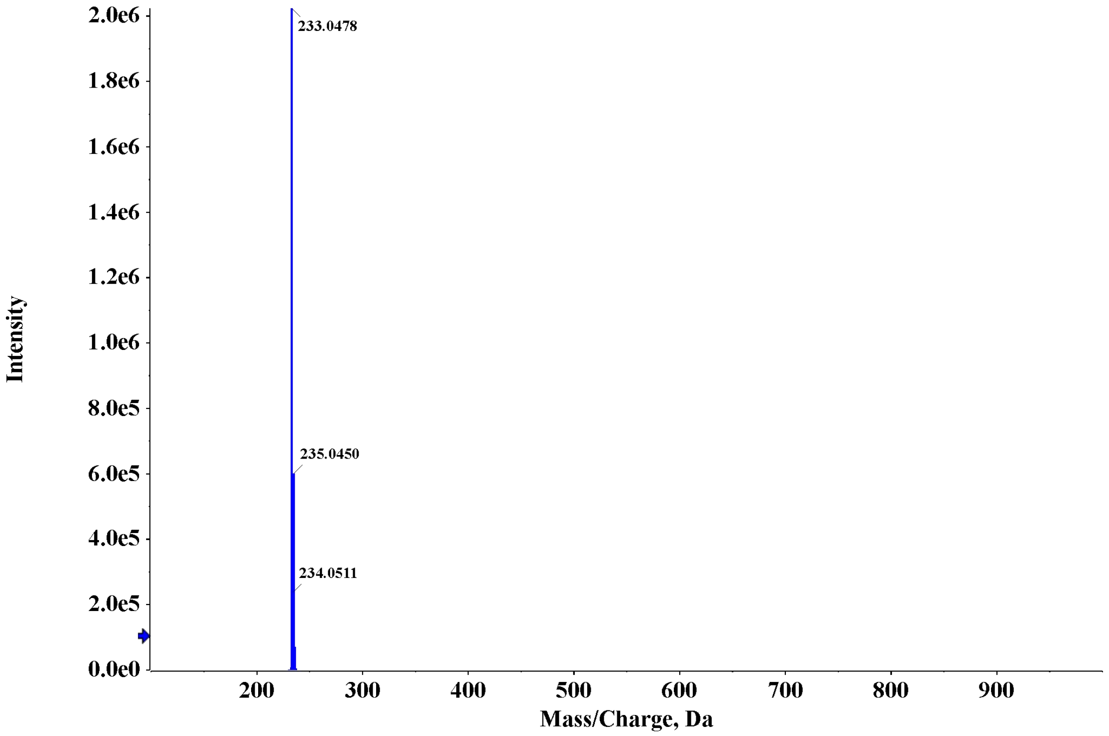

Compound 4c

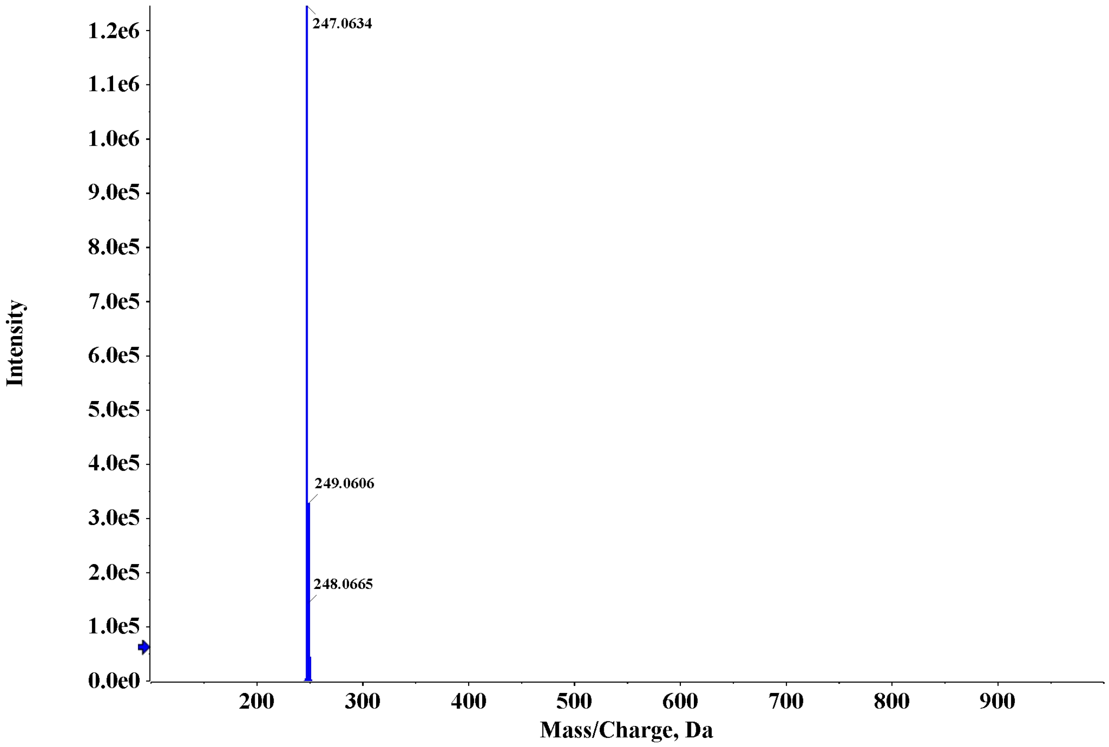

Compound 4d

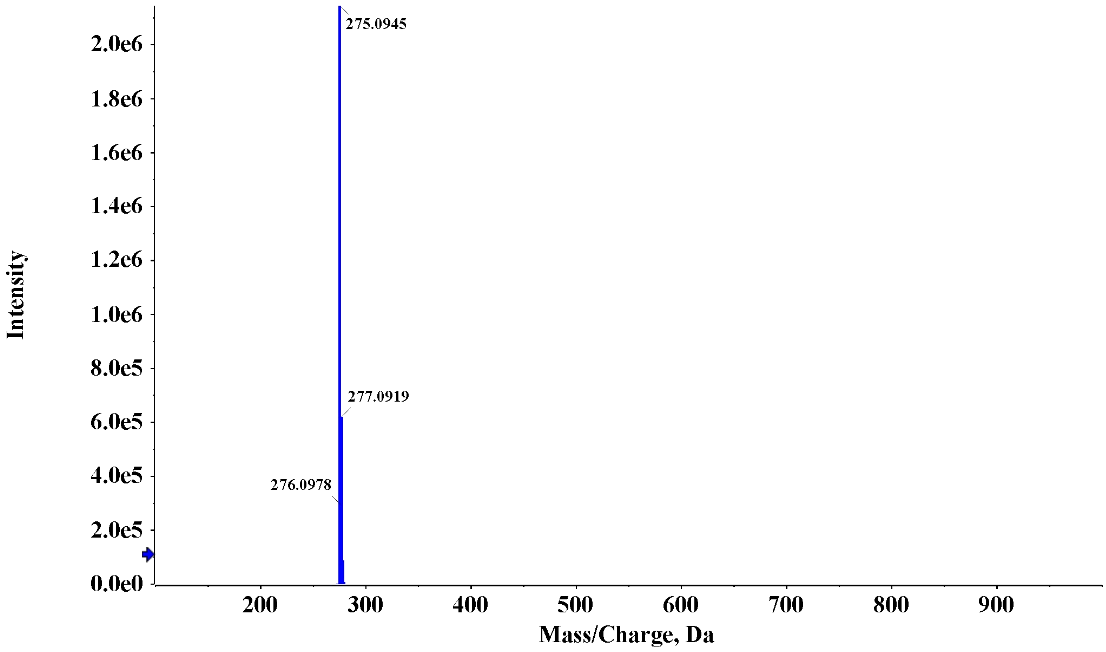

Compound 4e

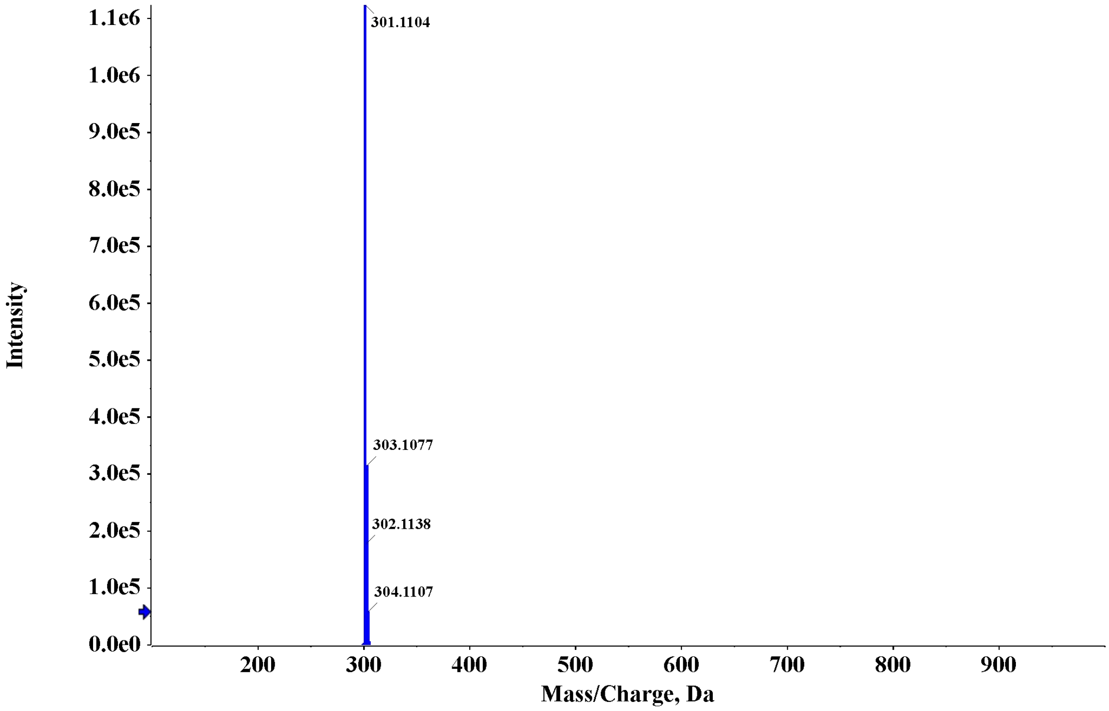

Compound 4f

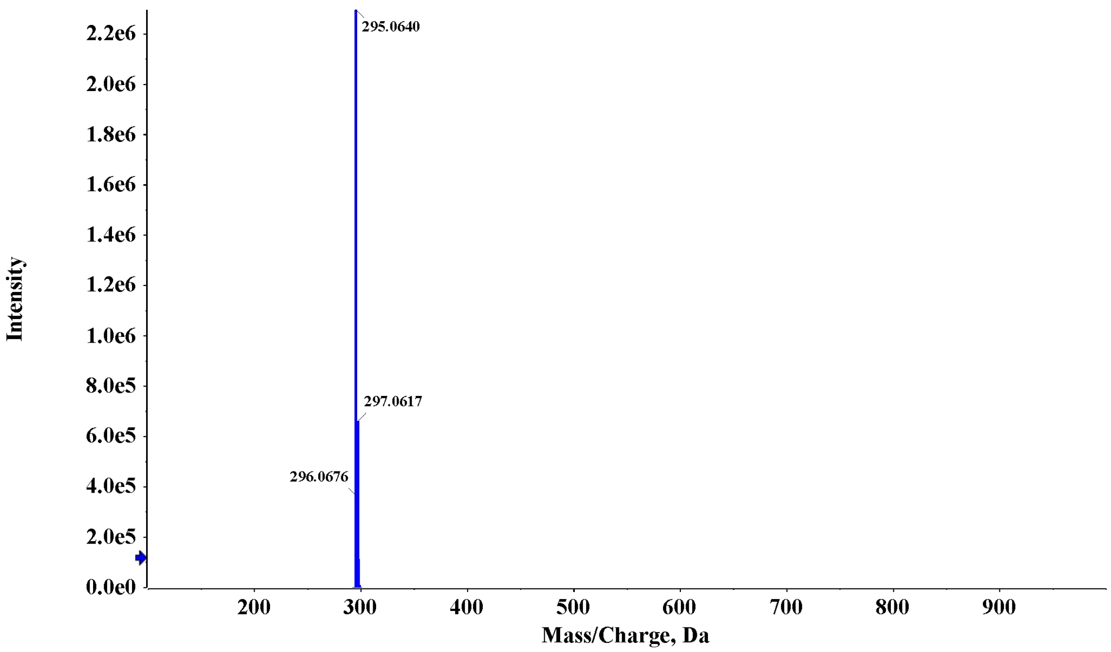

Compound 5a

Spectrum from 2.wiff (sample 1) - Sample003, +TOF MS (90 - 1000) from 2.194 to 2.202 min

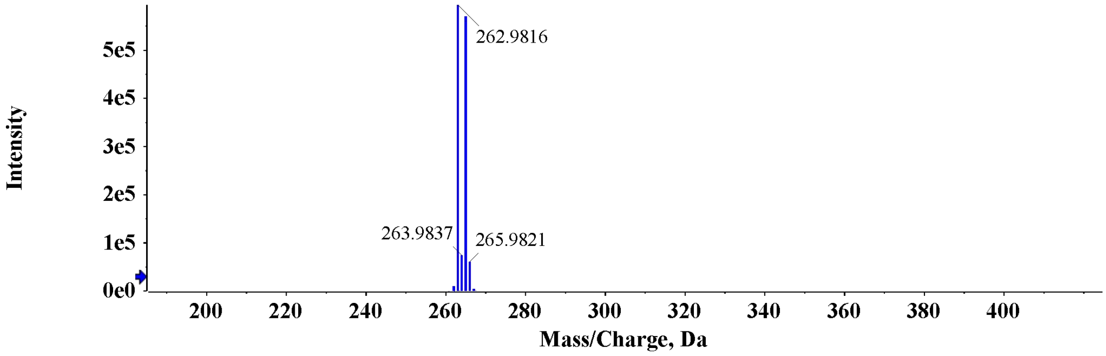

Compound 5b

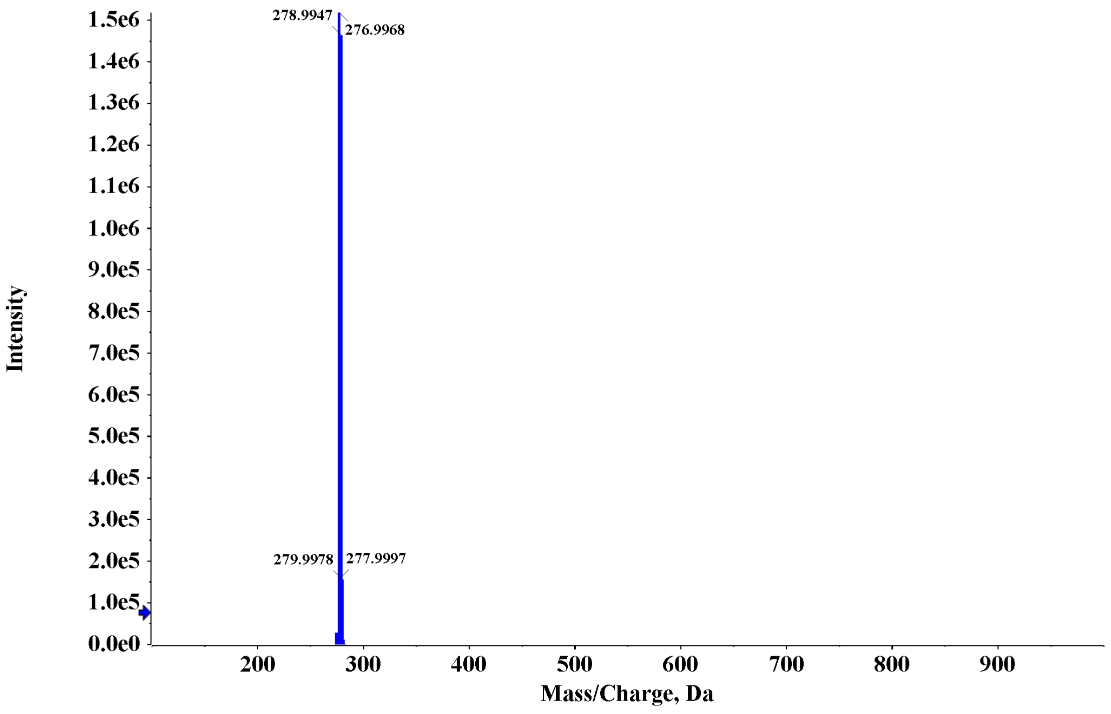

Compound 5c

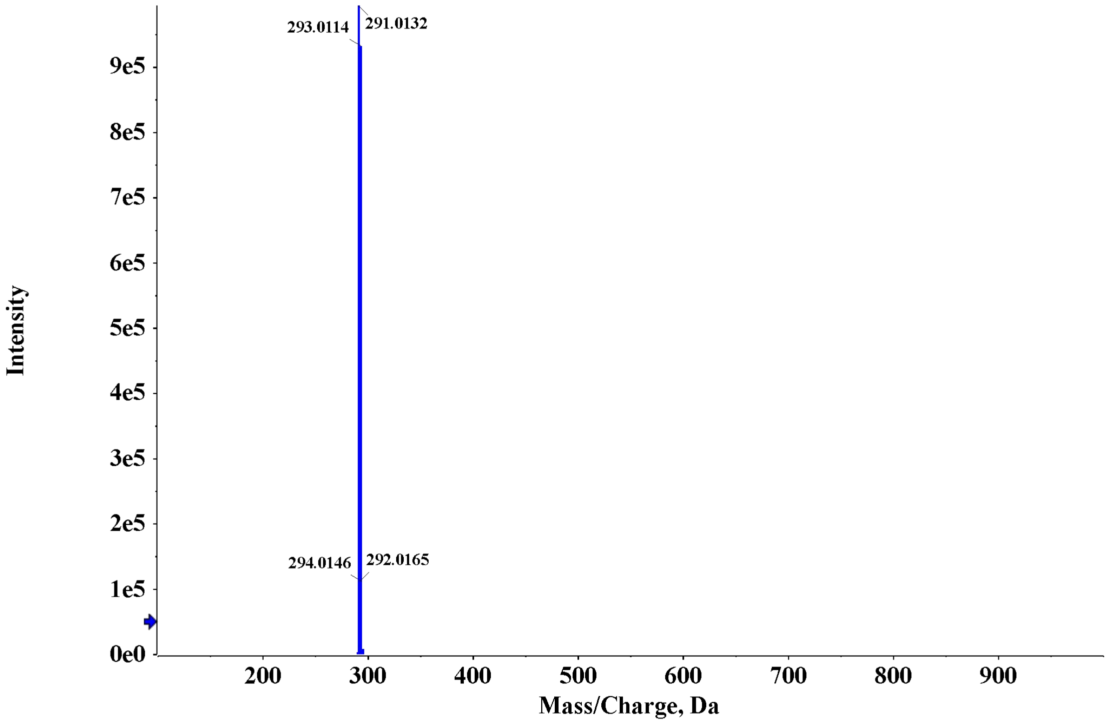

Compound 5d

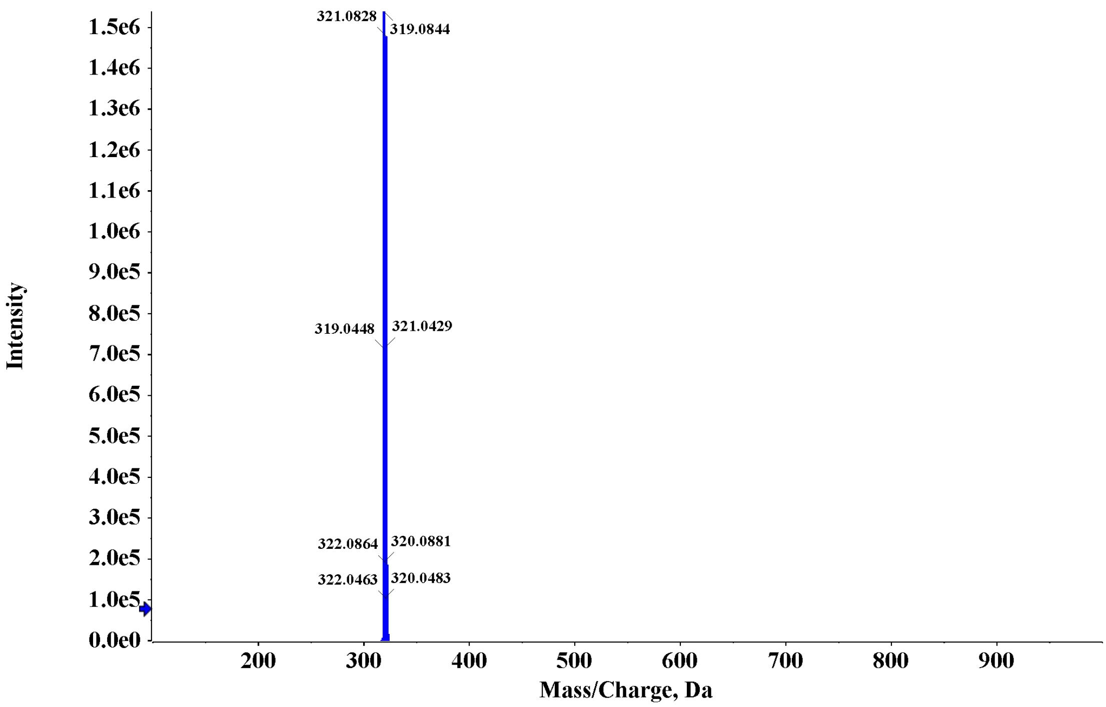

Compound 5e

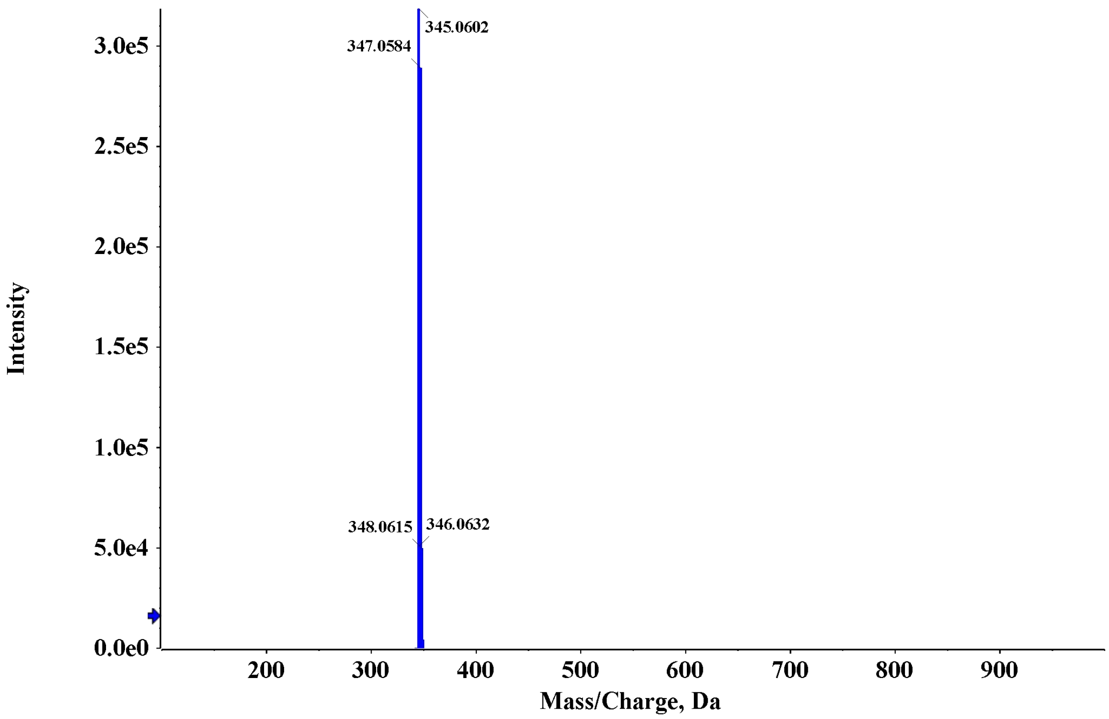

Compound 5f

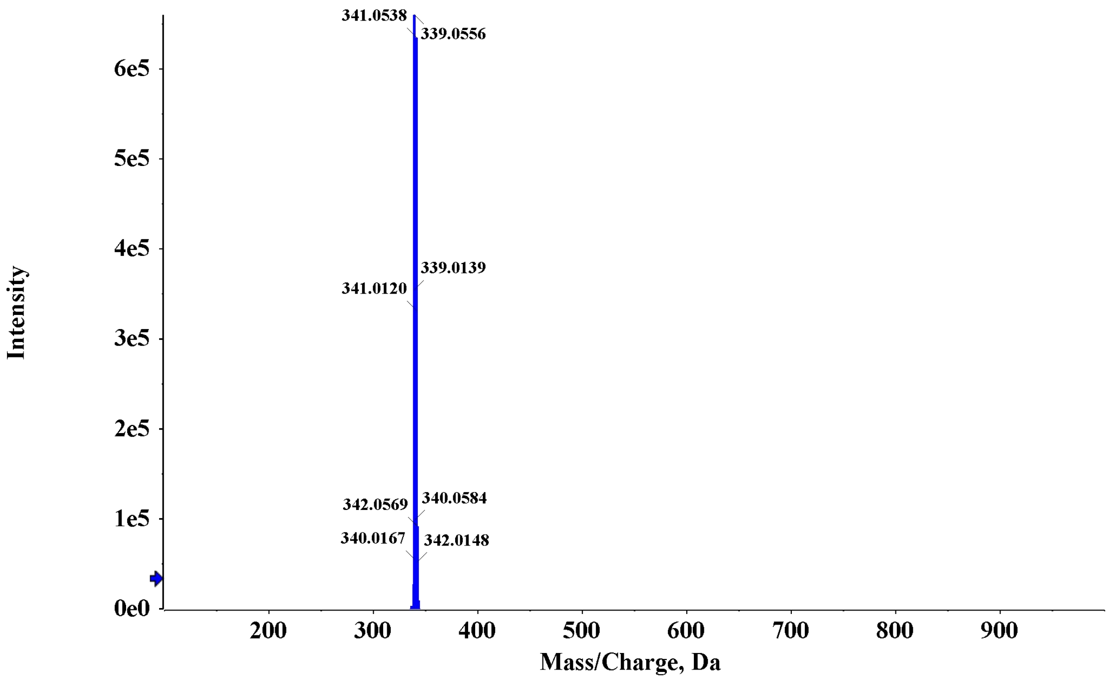

Compound 5g

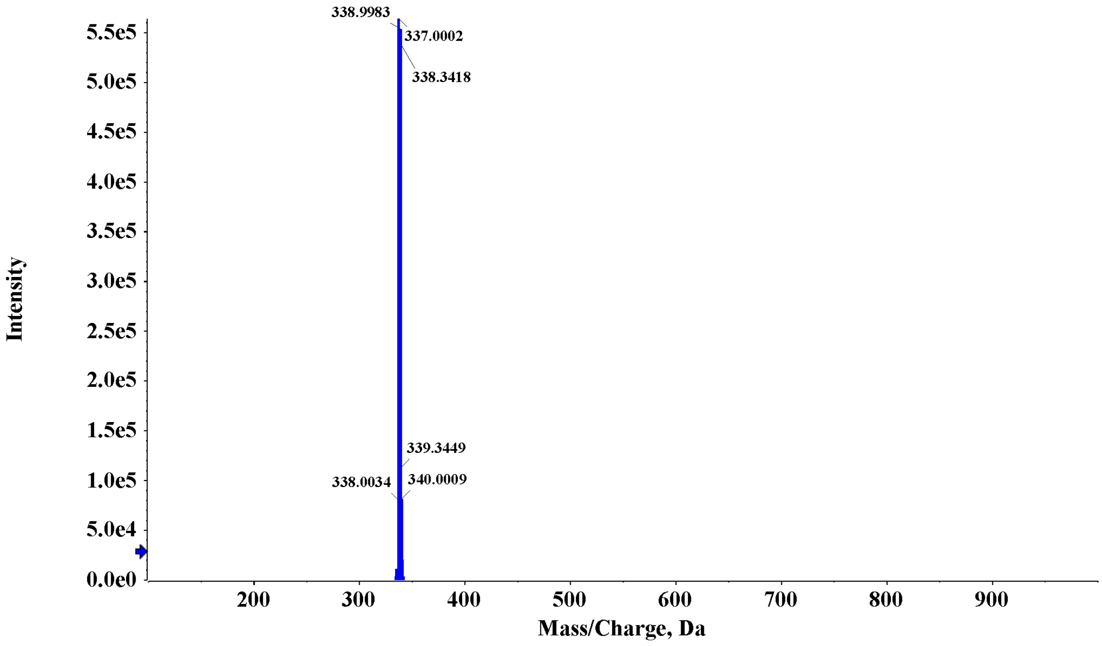

Compound 8a

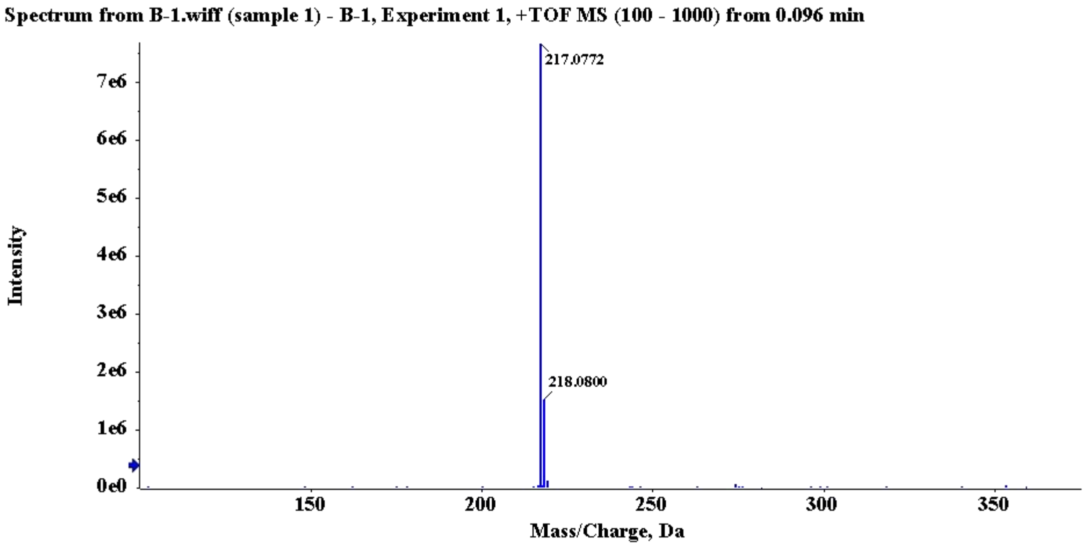

Compound 8b

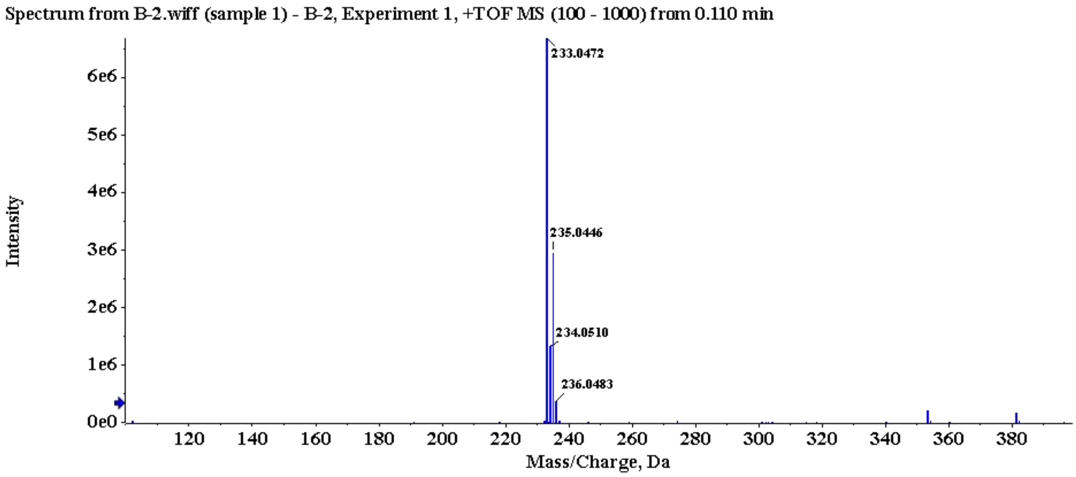

Compound 8c

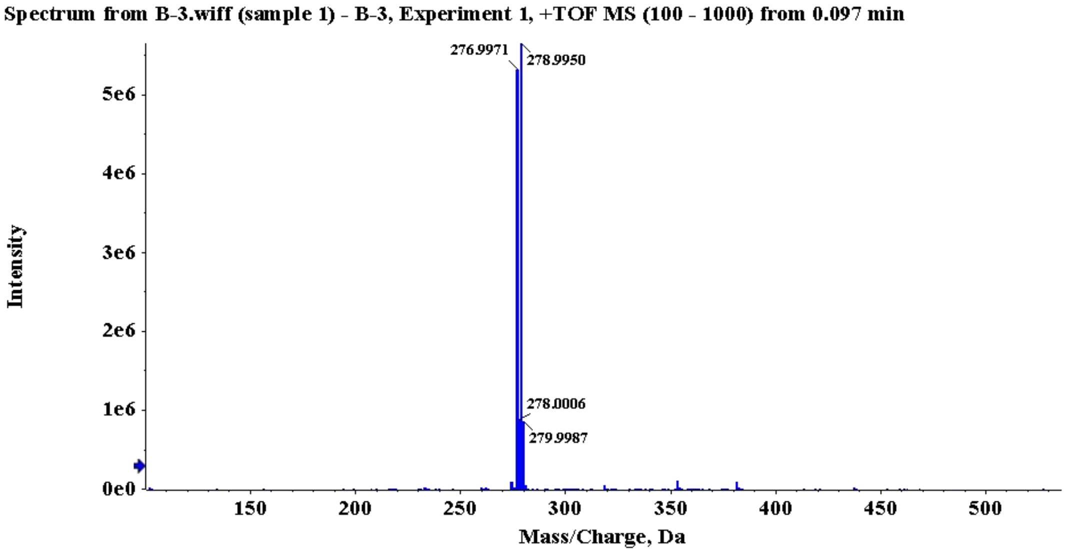

Compound 8d

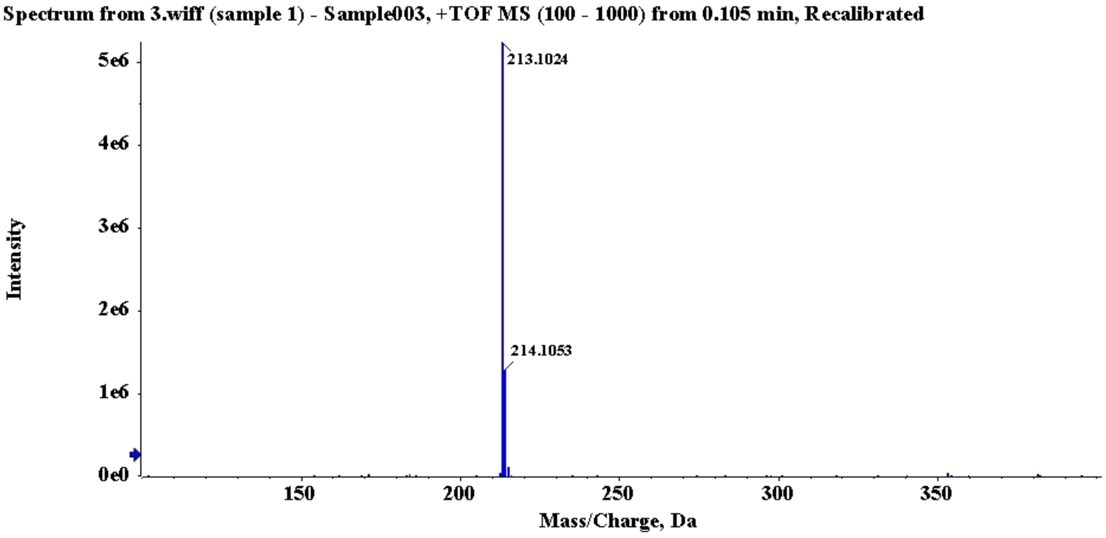

Compound 8e

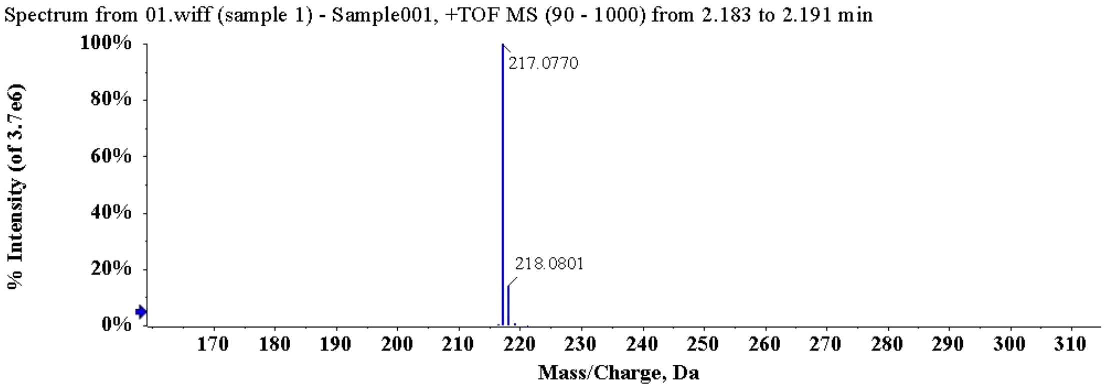

Compound 8f

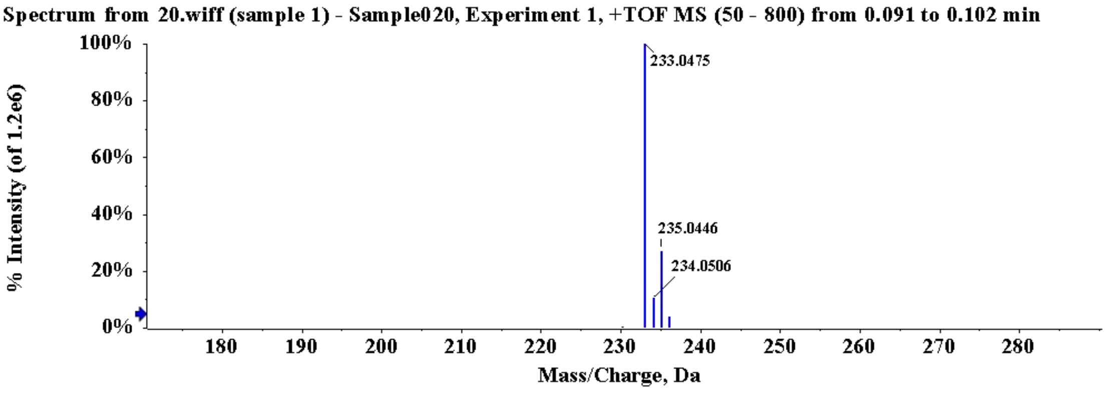

Compound 8g

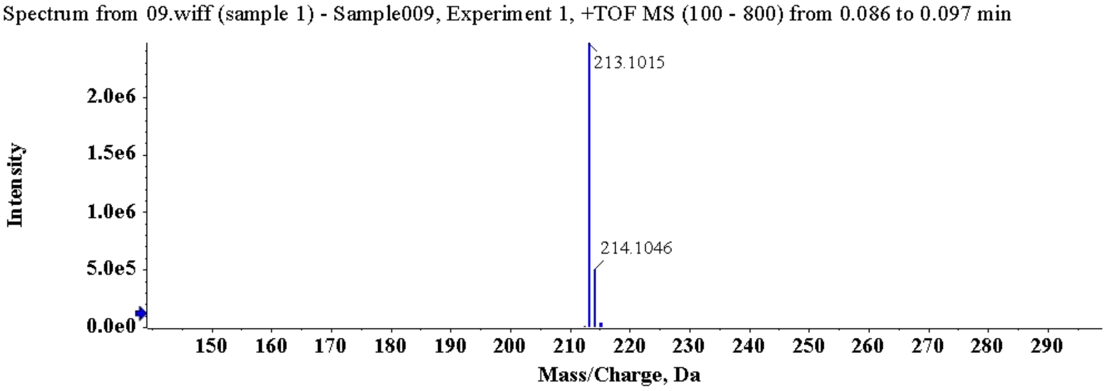

Compound 8h

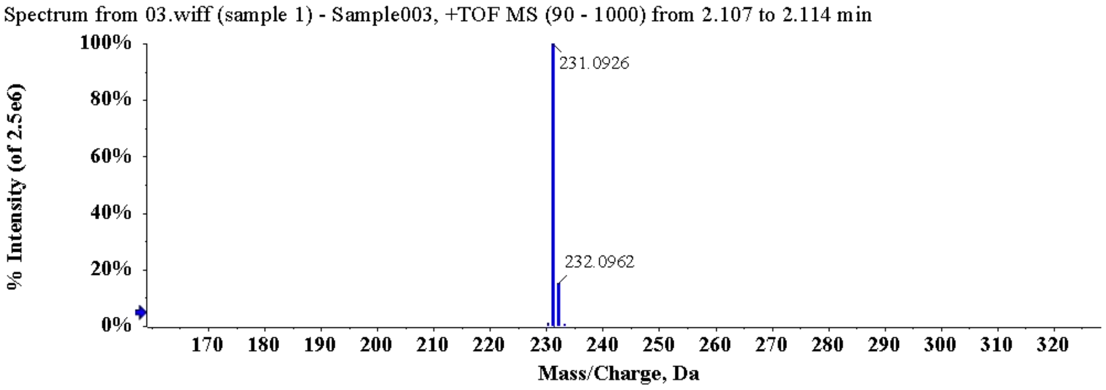

Compound 8i

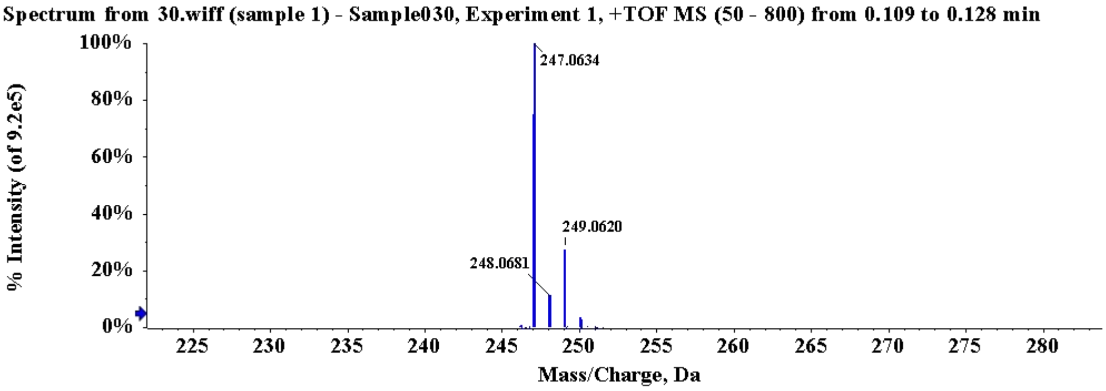

Compound 8j

Spectrum from 06.wiff (sample 1) - Sample006, Experiment 1, +TOF MS (100 - 800) from 0.096 to 0.107 min

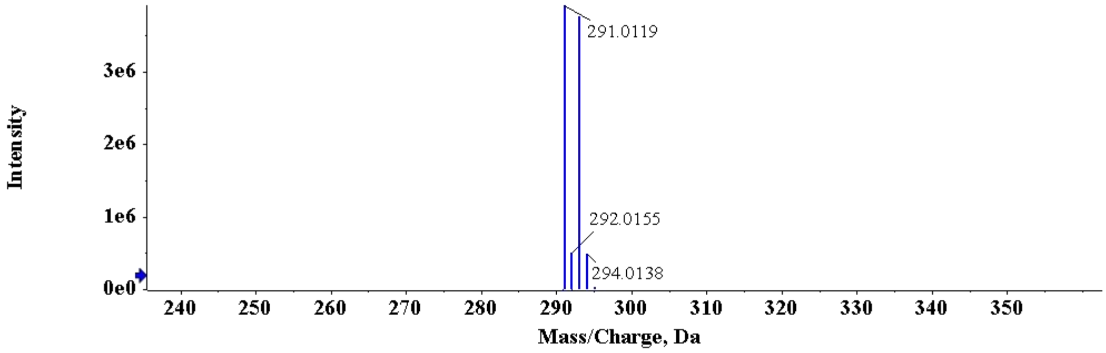

Compound 8k

Spectrum from 10.wiff (sample 1) - Sample010, Experiment 1, +TOF MS (100 - 800) from 0.101 to 0.112 min

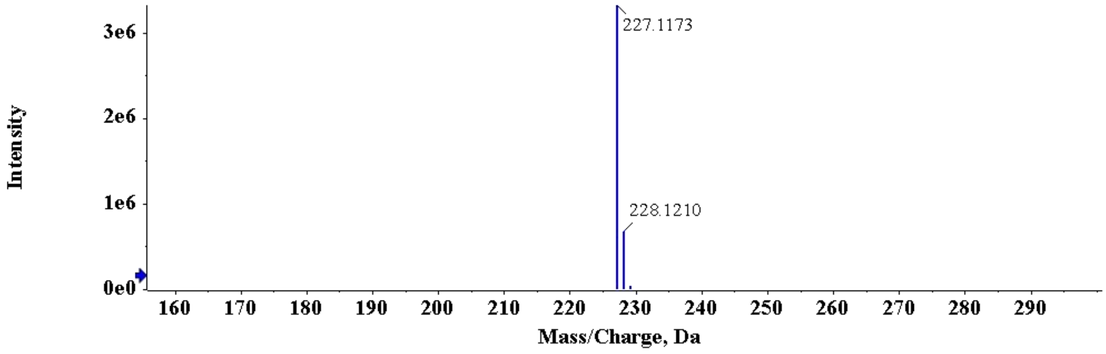

Compound 9a

Spectrum from B-5.wiff (sample 1) - B-5, Experiment 1, +TOF MS (100 - 1000) from 0.096 min

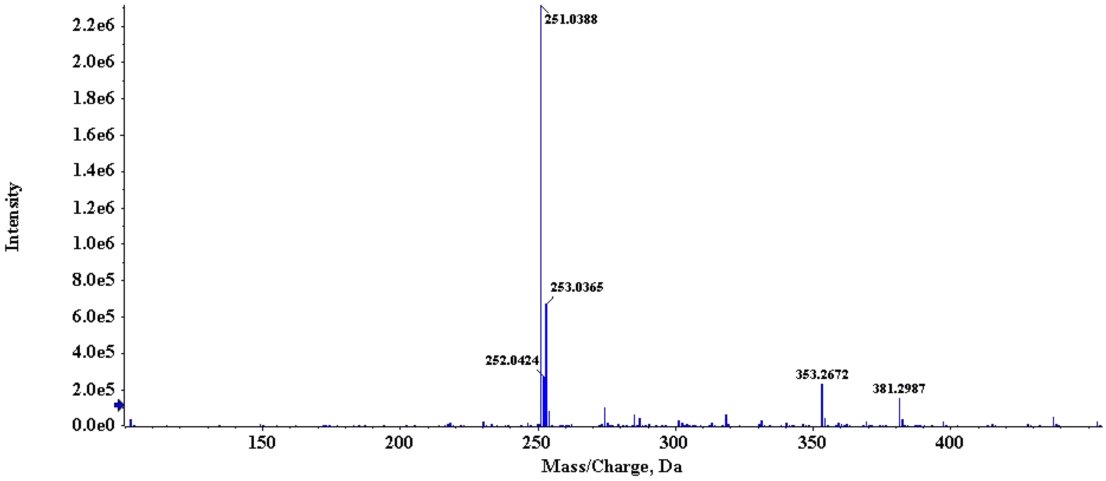

Compound 9b

Spectrum from B-6.wiff (sample 1) - B-6, Experiment 1, +TOF MS (100 - 1000) from 0.095 min

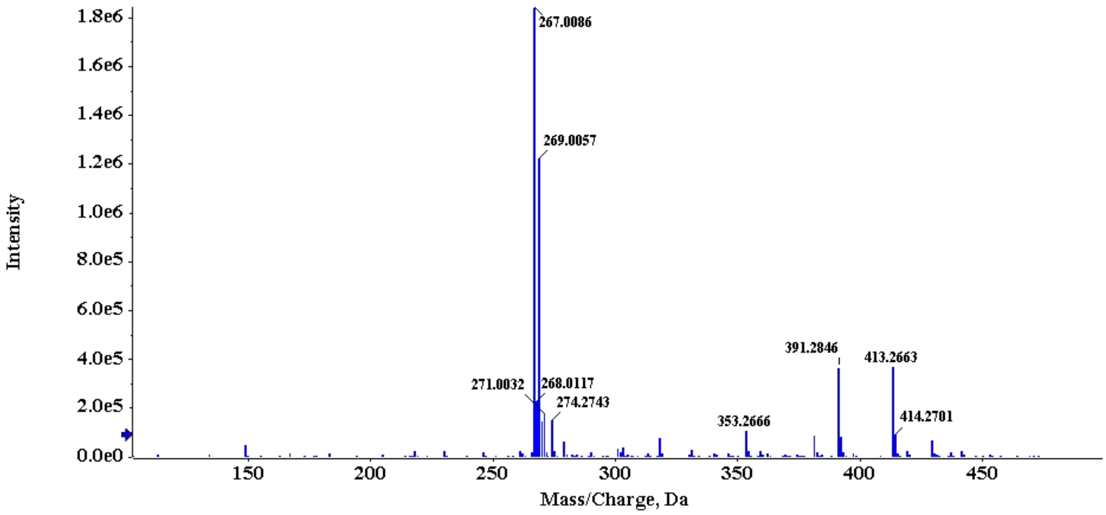

Compound 9c

Spectrum from B-7.wiff (sample 1) - B-7, Experiment 1, +TOF MS (100 - 1000) from 0.092 min

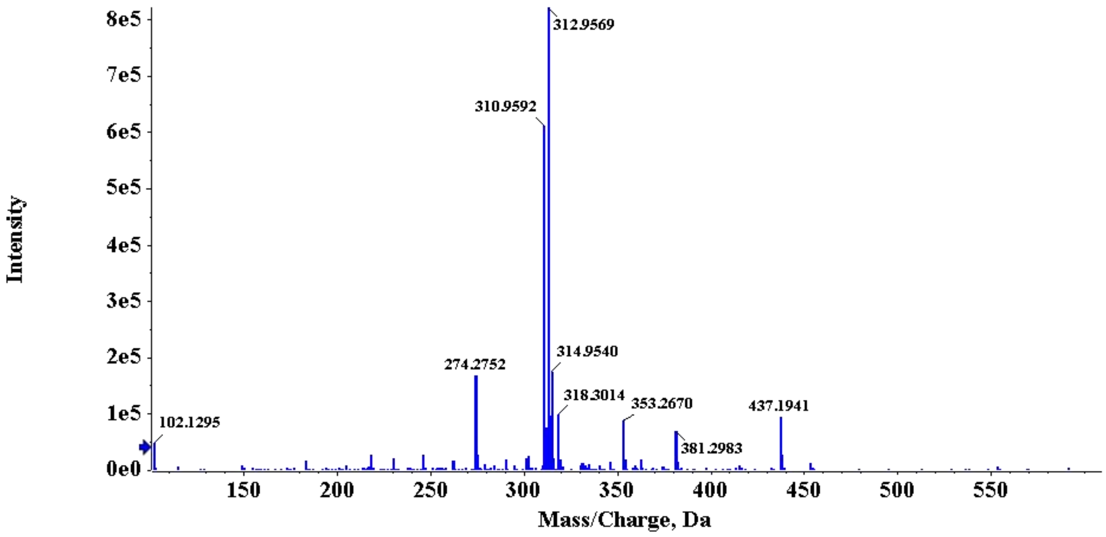

Compound 9d

Spectrum from 25.wiff (sample 1) - Sample025, Experiment 1, +TOF MS (50 - 800) from 0.103 to 0.114 min

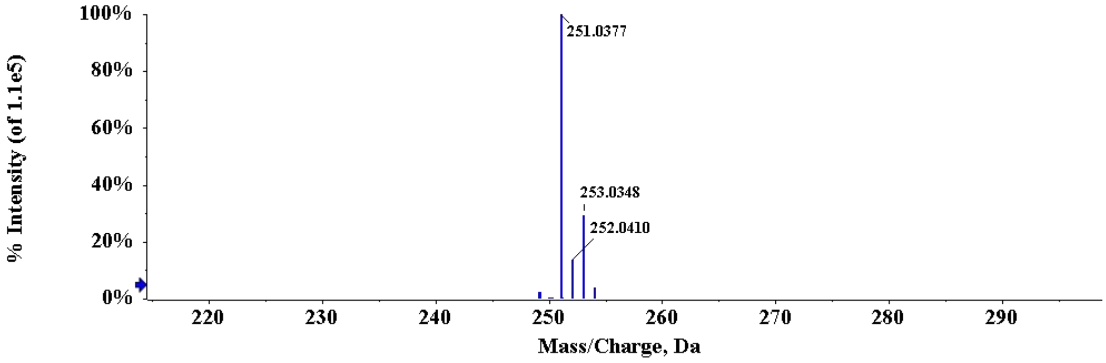

Compound 9e

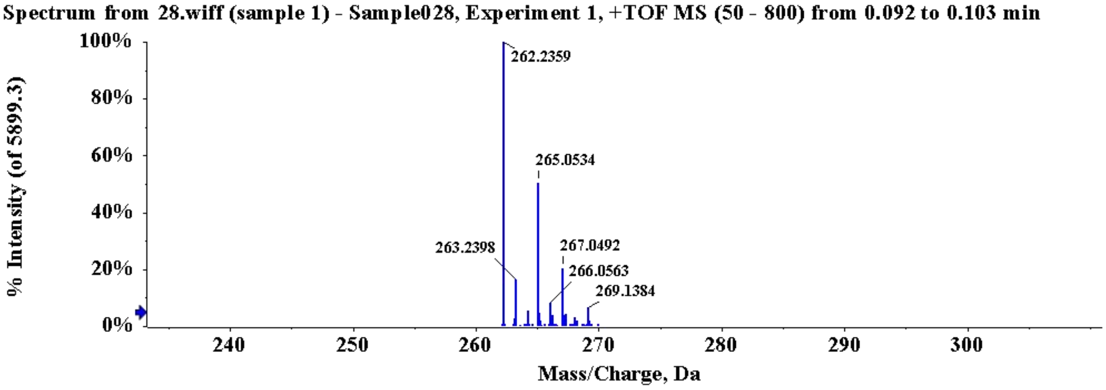

Compound 9f

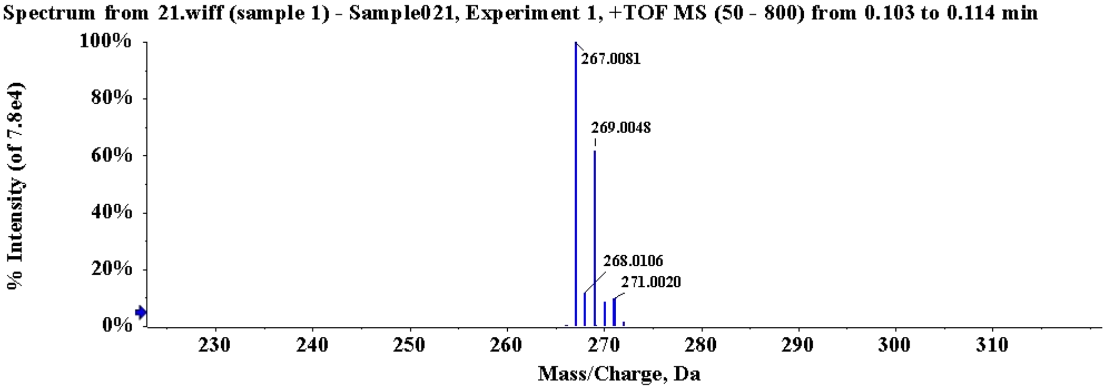

Compound 9g

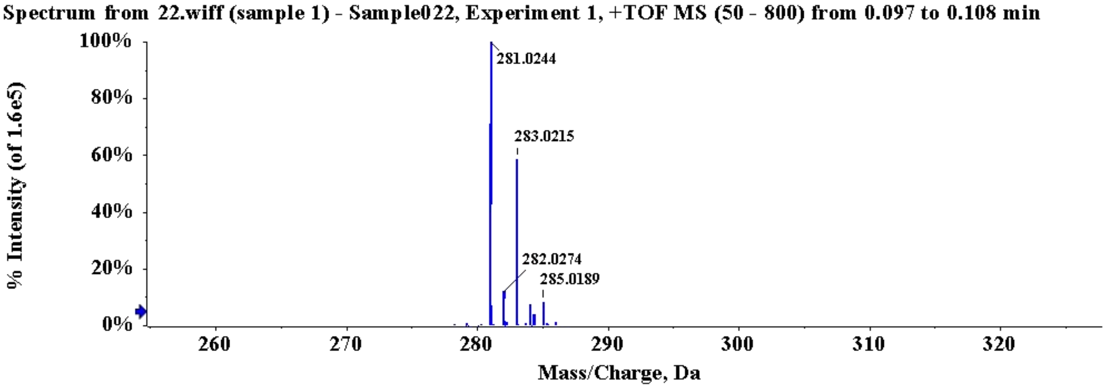

Compound 9h

Spectrum from 07.wiff (sample 1) - Sample007, Experiment 1, +TOF MS (100 - 800) from 0.102 to 0.113 min

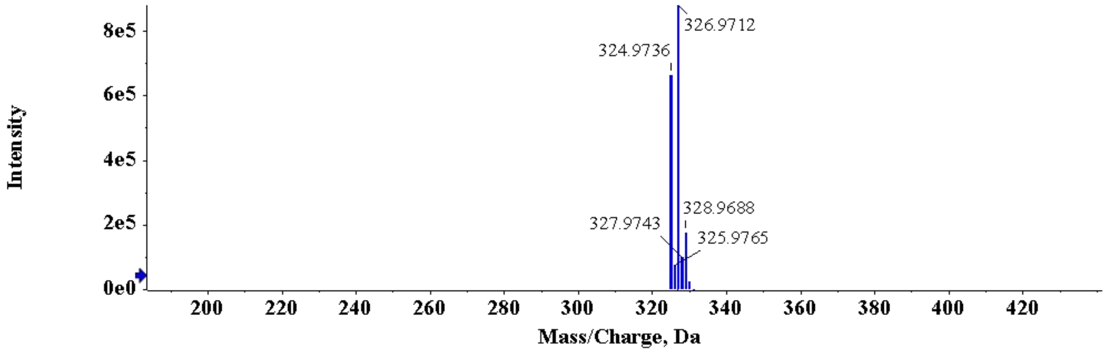

Compound 9i

Spectrum from 04.wiff (sample 1) - Sample004, +TOF MS (90 - 1000) from 2.381 to 2.389 min

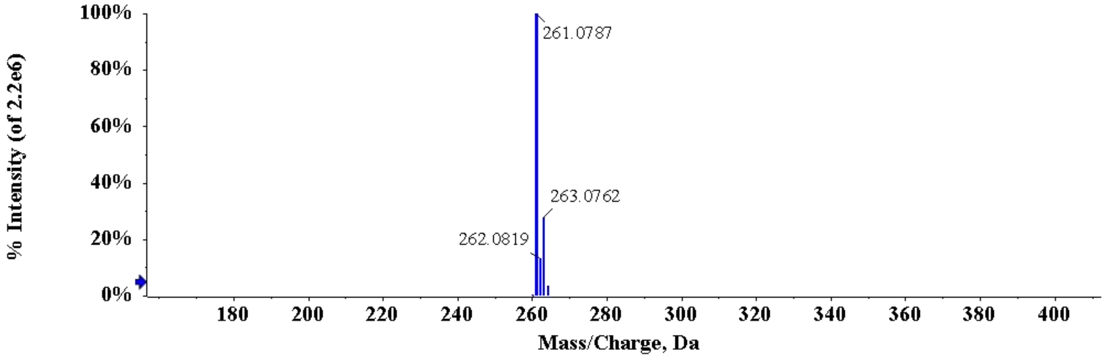

Compound 10a

Spectrum from B-9.wiff (sample 1) - B-9, Experiment 1, +TOF MS (100 - 1000) from 0.088 min, Recalibrated

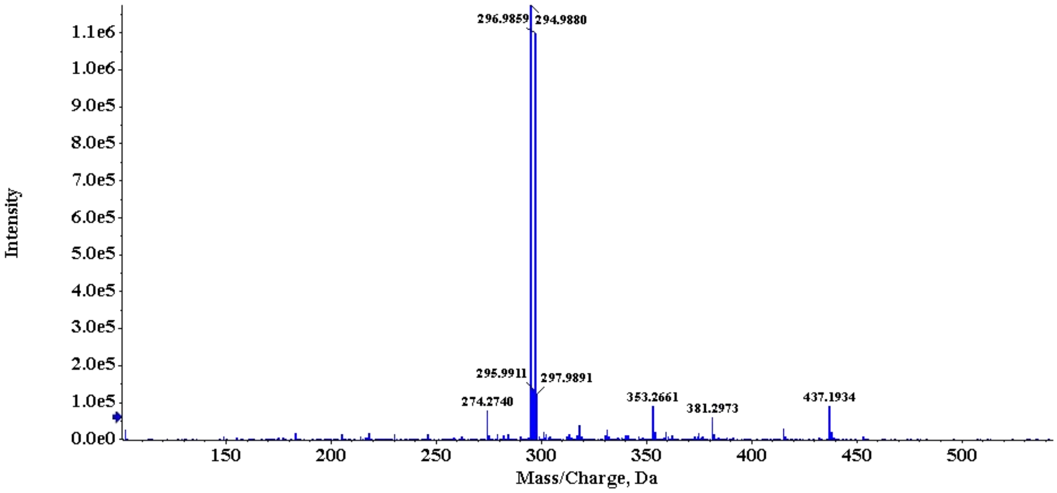

Compound 10b

Spectrum from B-10.wiff (sample 1) - B-10, Experiment 1, +TOF MS (100 - 1000) from 0.096 min, Recalibrated

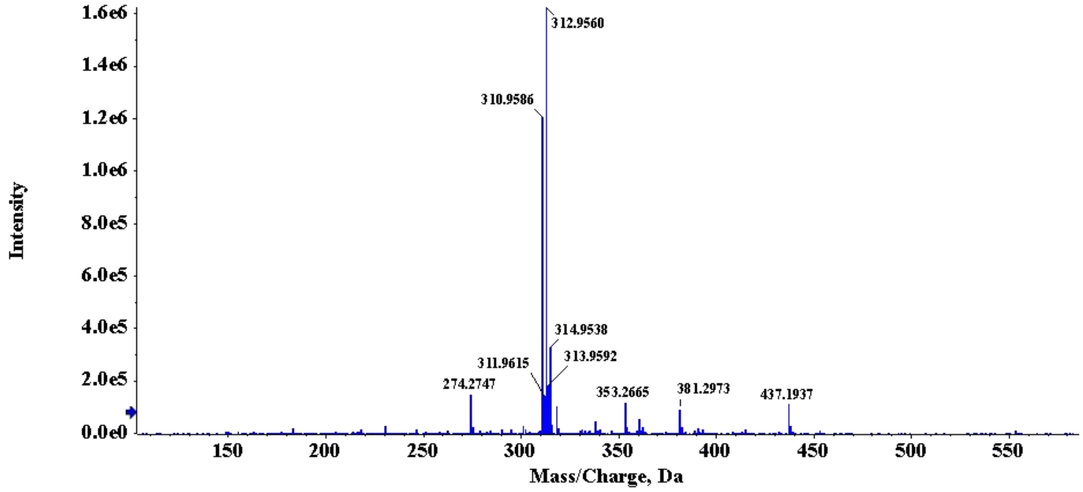

Compound 10c

Spectrum from B-11.wiff (sample 1) - B-11, Experiment 1, +TOF MS (100 - 1000) from 0.096 min, Recalibrated

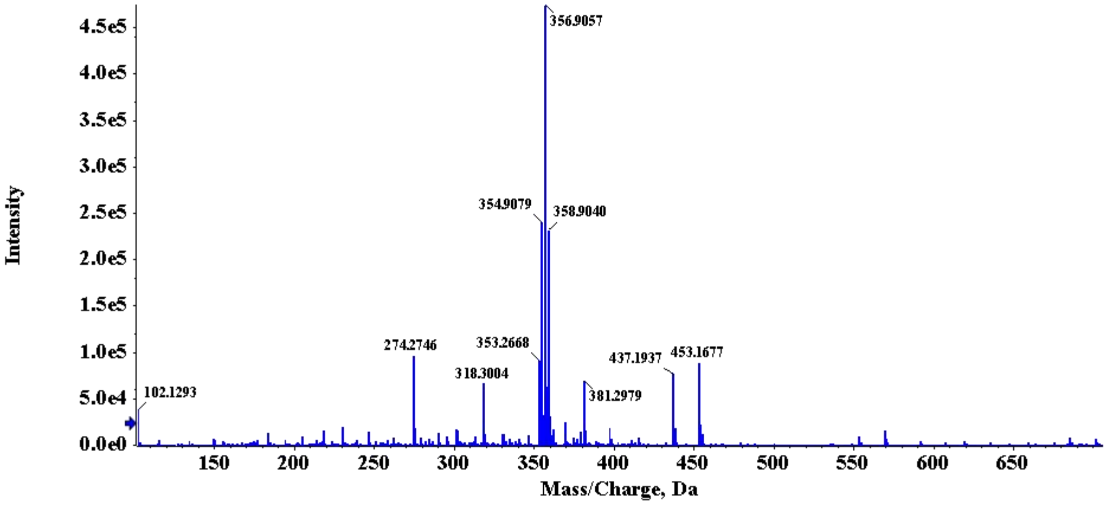

Compound 10d

Spectrum from B-12.wiff (sample 1) - B-12, Experiment 1, +TOF MS (100 - 1000) from 0.108 min

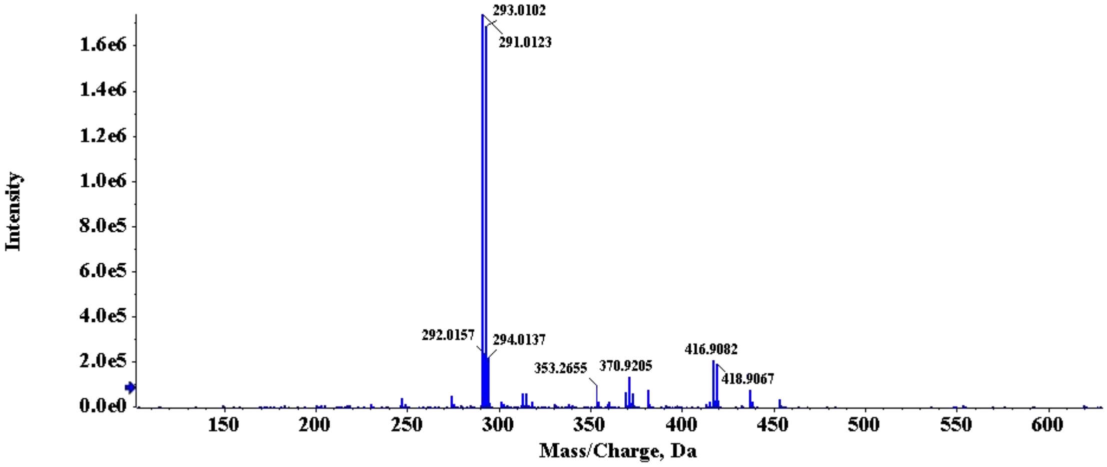

Compound 10e

Spectrum from 02.wiff (sample 1) - Sample002, +TOF MS (90 - 1000) from 2.194 to 2.202 min

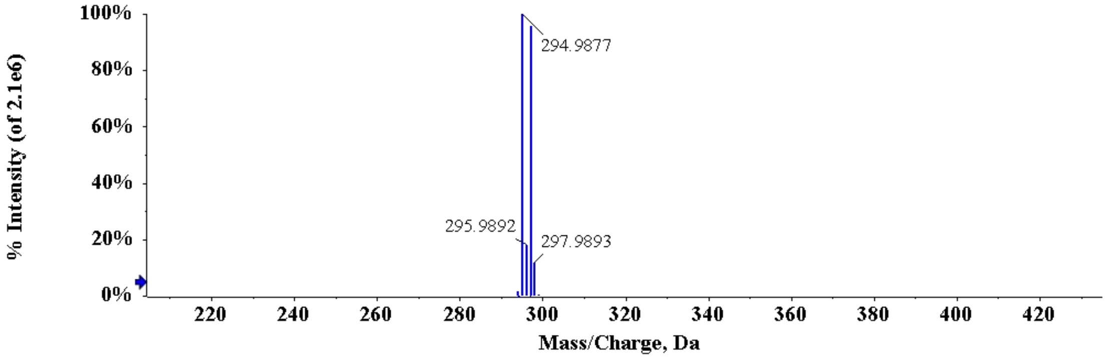

Compound 10f

Spectrum from 05.wiff (sample 1) - Sample005, Experiment 1, +TOF MS (100 - 800) from 0.096 to 0.107 min

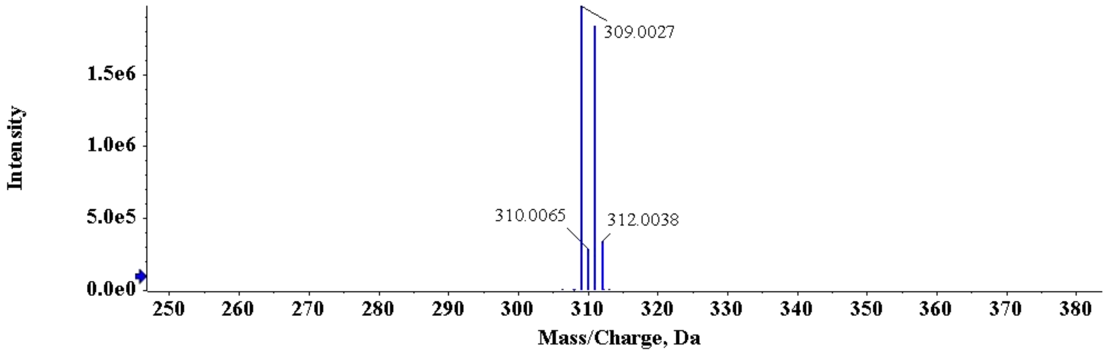

Compound 10g

Spectrum from 29.wiff (sample 1) - Sample029, Experiment 1, +TOF MS (50 - 800) from 0.097 to 0.108 min

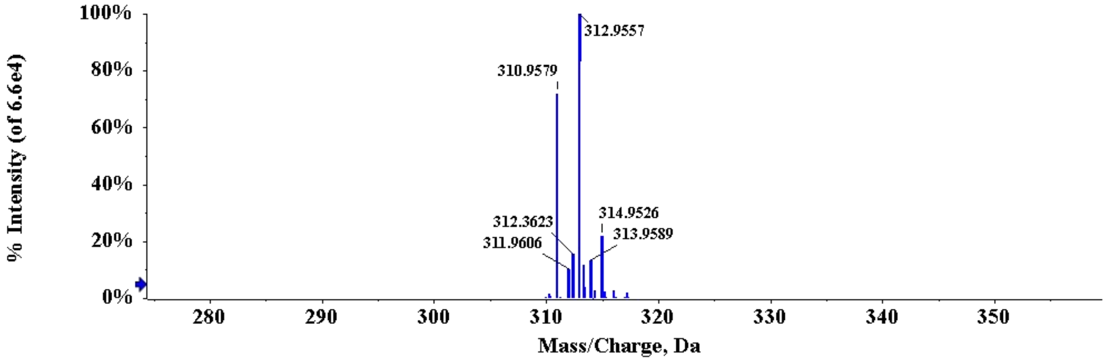

Compound 10h

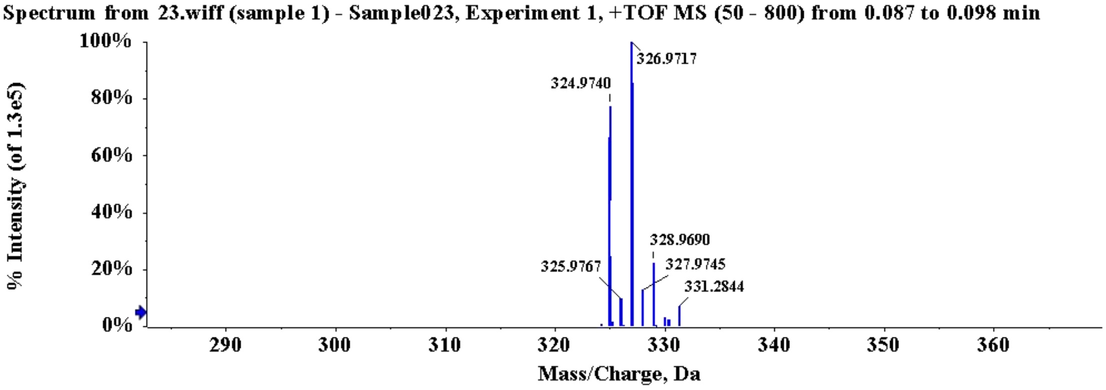

Compound 10i

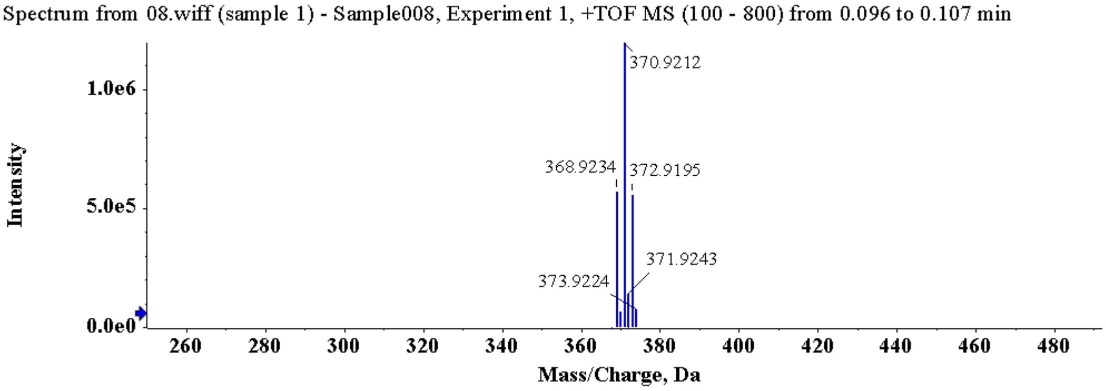

Supplement: Supplementary file 1 [file marinedrugs-20-00740-s001.zip › marinedrugs-2054963-supplementary.pdf]
